# Synthesis of Azide-Labeled β-Lactosylceramide Analogs Containing Different Lipid Chains as Useful Glycosphingolipid Probes †

**DOI:** 10.3390/molecules30132667

**Published:** 2025-06-20

**Authors:** Basant Mohamed, Rajendra Rohokale, Xin Yan, Amany M. Ghanim, Nermine A. Osman, Hanan A. Abdel-Fattah, Zhongwu Guo

**Affiliations:** 1 Department of Chemistry, University of Florida, Gainesville, FL 32611, USA; bmhusin@zu.edu.eg (B.M.); rajendra.rohokal@chem.ufl.edu (R.R.); x.yan@chem.ufl.edu (X.Y.); 2 Department of Pharmaceutical Organic Chemistry, Faculty of Pharmacy, Zagazig University, Zagazig 44519, Egypt; ammettwalli@pharmacy.zu.edu.eg (A.M.G.); namohamd@pharmacy.zu.edu.eg (N.A.O.); hahamied@pharmacy.zu.edu.eg (H.A.A.-F.); 3 UF Health Cancer Center, University of Florida, Gainesville, FL 32611, USA

**Keywords:** carbohydrates, glycolipids, glycosphingolipids, β-Lactosylceramide, lipids, lactose, azide, synthesis

## Abstract

β-Lactosylceramide (β-LacCer) is not only a key intermediate in the biosynthesis of complex glycosphingolipids (GSLs) but also an important regulator of many biological processes. To facilitate the investigation of β-LacCer and other GSLs, a series of β-LacCer analogs with an azido group at the 6-*C*-position of the D-galactose in lactose and varied forms of the ceramide moiety were synthesized from commercially available lactose in sixteen linear steps by a versatile and diversity-oriented strategy, which engaged lipid remodeling and glycan functionalization at the final stage. These azide-labeled β-LacCer analogs are flexible and universal platforms that are suitable for further functionalization with other molecular tags via straightforward and biocompatible click chemistry, thereby paving the way for their application to various biological studies.

## 1. Introduction

Glycosphingolipids (GSLs) are ubiquitous and abundant glycolipids in the cell membrane [1] that are composed of a hydrophilic glycan head and a lipophilic ceramide (Cer) tail coupled together by a glycosidic bond [2,3]. The amphiphilic property of GSLs enables them to adhere to the cell membrane outer leaflet and traffic through the lipid bilayer [4,5]. Inside the cell membrane, GSLs tend to self-aggregate in specific microdomains of the lipid rafts, where signaling molecules are localized and enriched [6,7]. As a result, GSLs play an important role in numerous biophysiological processes, such as cellular recognition and signaling, cell differentiation and proliferation, etc. [8]. GSL dysregulation is also associated with many human diseases, including cancer and Alzheimer’s disease [9,10]. For example, a number of GSLs have been identified as tumor-associated carbohydrate antigens (TACAs) and utilized as molecular targets for the development of therapeutic cancer vaccines [9,11].

**Figure 1:**
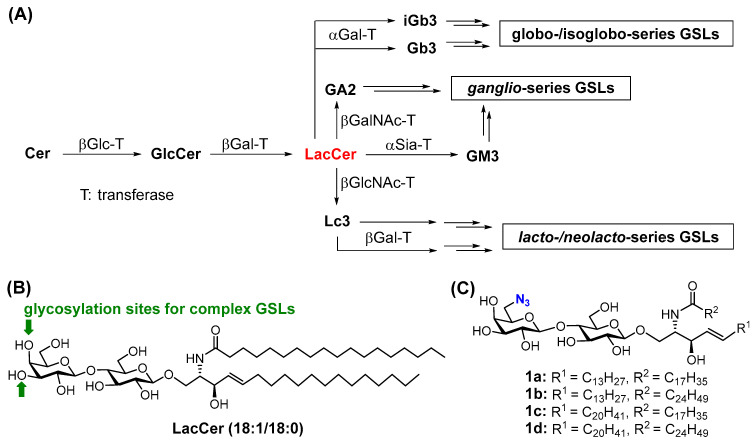
(**A**) The biosynthetic pathways for *globo*-, *isoglobo*-, *ganglio*-, *lacto*-, and *neolecto*-series GSLs, utilizing β-LacCer as the key intermediate, and some key enzymes involved, and structures of (**B**) a representative β-LacCer with the most common (18:1/18:0) lipid form found in mammals and (**C**) designed β-LacCer analogs with different lipid forms as useful probes to study β-LacCer and other GSLs.

Natural GSLs are structurally diverse, with potential structural variations in both the glycan and the Cer moiety [1]. β-Lactosylceramide (β-LacCer) is a simple GSL having the D-glucose (Glc) residue of lactose, a disaccharide, β-linked to the Cer moiety. In mammals, it is the key intermediate during the biosynthesis of the majority of complex GSLs, e.g., *globo*-, *isoglobo*-, *ganglio*-, *lacto*-, and *neolecto*-series GSLs (Figure 1A) [12,13]. In the meantime, it has been demonstrated that β-LacCer is involved in the human central nervous system, immune system, and diseases like cancer, inflammation, and neurodegeneration [14,15,16]. For instance, β-LacCer overexpression is found in leukemia, renal cancer, and cholangiocarcinoma [17], and LacCer accumulation is also related to atherosclerosis and aberrant autophagy [18,19]. Thus, β-LacCer is identified as a drug target [20]. Additionally, similar to other GSLs, the lipids in β-LacCer usually contain 16–20 carbons (C16–20) (such as C18, Figure 1B), while the forms of β-LacCer with longer (>C24) lipid chains are also found in neutrophils, which become molecular targets for the treatment of immunological disorders [21,22,23].

However, detailed investigation of β-LacCer, especially within complex matrices like the lipid membrane, is challenging due to the lack of fluorophores and other visible functionalities in its structure. To address the problem, it is necessary to functionalize β-LacCer with molecular tags (e.g., affinity and fluorescent tags) to facilitate various modern analytical technologies. In this context, we designed a series of β-LacCer analogs **1a**–**d** (Figure 1C) containing an azido group and diverse lipid chains and synthesized them by a diversity-oriented strategy. These β-LacCer analogs should be useful molecular tools.

## 2. Results and Discussion

In the designed β-LacCer probes **1a**–**d** (Figure 1C), we planned to attach an azido group to the 6-*C*-position of D-galactose (Gal) in the lactose moiety for several reasons. First, the azido group is a flexible platform to facilitate further functionalization of **1a**–**d** with various molecular labels through straightforward and biocompatible click chemistry [24,25]. Next, the azido group is small—not significantly larger than a hydroxyl group, and it is expected to have a minimal impact on the properties of β-LacCer. Consequently, if **1a**–**d** are used as biosynthetic precursors for metabolic glycoengineering, they are likely to be acceptable by enzymes. Moreover, the 6-*C*-position of the Gal residue in lactose is distinct, because it is a primary carbon at the non-reducing end and is probably the least sterically hindered position, making it relatively efficient and straightforward to functionalize this position and subsequently modify the synthetic probes. The designed probes also contain different forms of lipids, which will facilitate the investigation of how lipid structures of β-LacCer influence its chemical, biophysical, and biological properties.

**Scheme 1:**
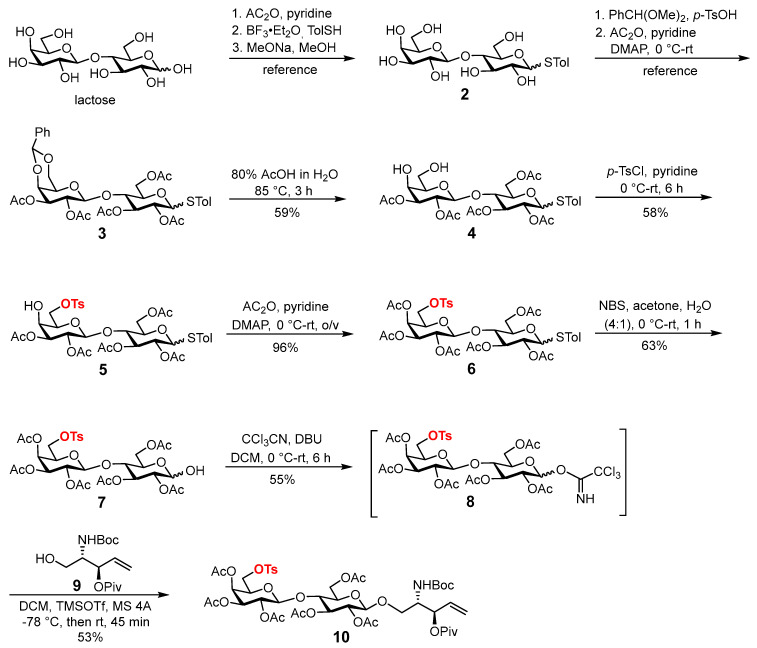
Preparation of **10**—the key and common building block for the synthesis of all designed probes in the present work.

Our synthesis of the target molecules **1a**–**d** (Scheme 1) started with commercially available and inexpensive lactose. It was first converted into the *O*-acetyl and 4′,6′-*O*-benzylidene protected glycoside of *p*-toluenethiol (TolSH) **3** as an α,β-isomeric mixture via a series of well-established reactions reported in the literature [26,27,28,29]. Next, the 4′,6′-*O*-benzylidene group in **3** was selectively removed upon treatment with 80% acetic acid in water at 85 °C to afford diol **4** as an anomeric mixture. Selective tosylation of the 6′-OH group in **4** was achieved by its reaction with *p*-toluenesulfonylchloride (*p*-TsCl) and 4-dimethylaminopyridine (DMAP). The regioselective attack of *p*-TsCl to the 6′-OH group is probably because this primary hydroxyl group is less sterically hindered than the secondary 4′-OH group in **4**. The equivalence and concentration of *p*-TsCl, along with the order to add reagents, are also substantial in the control of this regioselectivity. The formation of **5** was confirmed by the downfield shifts of its 6′-H NMR signals, compared to those of **4** (from δ 4.06 and 3.72 to 4.41 and 4.07 ppm), and the appearance of ^1^H signals of an additional toluene moiety at δ 7.40–7.35 and 2.47 ppm, respectively, in the ^1^H NMR spectrum of **5**. The free hydroxyl group remaining in **5** was then acetylated using acetic anhydride, pyridine, and DMAP. To reveal the reducing-end anomeric position for glycosidation, the resultant **6** was applied to oxidative hydrolysis with *N*-bromosuccinimide (NBS) in water and acetone (1:4) to afford hemiacetal **7** (63%) as an anomeric mixture, together with unreacted **6** (34%). Next, **7** was converted into imidate **8** as a glycosyl donor upon reacting with trichloroacetonitrile catalyzed by 1,8-diazabicyclo-[5.4.0]-undec-7-ene (DBU). After brief purification, the reactive glycosyl imidate **8** was immediately submitted to the glycosylation reaction with Cer precursor **9** [30,31], utilizing trimethylsilyltrifluoromethanesulfonate (TMSOTf) as the promotor. This reaction produced **10** as the key synthetic intermediate in excellent stereoselectivity, and the β-configuration of its newly formed glycosidic bond is confirmed by the large coupling constant (*J* = 7.8 Hz) of its anomeric proton at δ 4.43 in the ^1^H NMR spectrum. The common intermediate **10** was then used for diversity-oriented synthesis of all β-LacCer probes **1a**–**d**.

**Scheme 2:**
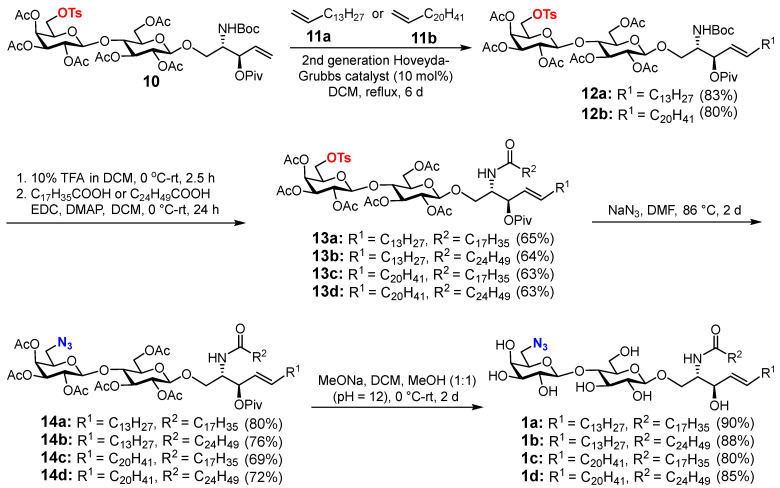
Assembly of the designed β-LacCer probes **1a**–**d**.

The final steps for **1a**–**d** synthesis include lipid remodeling, glycan functionalization, and global deprotection, as outlined in Scheme 2. Olefin cross-metathesis of pentadecene **11a** or docosene **11b** with **10** in the presence of the 2nd generation Hoveyda−Grubbs catalyst [32] allowed on-site generation of the sphingosine moiety of varied chain lengths in very good yields (80–83%), although the reaction was rather slow, taking six days to complete [31]. The *E*-configuration of the C=C bond in **12a** and **12b** was verified by the olefinic ^1^H-^1^H coupling constant (*J* = 14.6–14.8 Hz) in their ^1^H NMR spectra. To attach the *N*-fatty acyl chain, the *tert*-butoxycarbonyl (Boc) group in **12a,b** was selectively removed with 10% trifluoroacetic acid (TFA) in dichloromethane (DCM) to afford the intermediate free amines, which were directly subjected to *N*-acylation employing stearic acid or pentacosanoic acid, with *N*-(3-dimethylaminopropyl)-*N′*-ethylcarbodiimide hydrochloride (EDC) and DMAP as the condensation reagents. These reactions gave 6′-*O*-tosylated β-LacCer derivatives **13a**–**d** containing different lipid chains in good overall yields (63–65%, two steps). Subsequently, nucleophilic substitution of the tosylate in **13a**–**d** with sodium azide in dimethylformamide (DMF) proceeded smoothly to provide fully protected LacCer analogs **14a**–**d** in high yields (69–80%). Eventually, all *O*-acyl groups in **14a**–**d** were removed with sodium methoxide in methanol and DCM (1:1) to produce the synthetic targets **1a**–**d** in 80–90% yields. The final products, as well as all new intermediates involved in these syntheses, have been fully characterized with NMR and high-resolution mass spectrometry (HR-MS) data (see data and Figures in Supporting Information).

## 3. Experimental Section

### 3.1. General Methods

Chemicals, reagents, and solvents were commercial and used without further purification. Molecular sieves 4 Å (MS 4Å) were flame-dried under a high vacuum and used after being cooled to room temperature (rt) under an N_2_ atmosphere. Thin-layer chromatography (TLC) was performed on silica gel 60 Å F254 plates, with detection utilizing a UV lamp at a wavelength of 254 nm followed by charring with 10% (*v*/*v*) H_2_SO_4_ in ethanol or ninhydrin and anisaldehyde staining. Flash column chromatography was performed using silica gel 60 (230–400 mesh). NMR spectra were acquired on a 400, 500, or 600 MHz spectrometer, with chemical shifts (δ) reported in ppm as referenced to internal tetramethylsilane (TMS) (^1^H NMR: δ 0.00 ppm), CDCl_3_ (^1^H NMR: δ 7.26 ppm; ^13^C{^1^H} NMR: δ 77.2 ppm), or CD_3_OD (^1^H NMR: δ 3.31 ppm; ^13^C{^1^H} NMR: δ 49.0 ppm). Coupling constants (*J*) were reported in Hz. Peak, and coupling constant assignments were based upon ^1^H NMR, ^1^H–^1^H COSY, and ^1^H–^13^C{^1^H} HSQC experiments. HR-MS spectra were recorded on the XEVO-G2-XS Q-TOF-ESI instrument. An aluminum heating block was used for heating reaction mixtures. Intermediates **2**, **3**, and **9** were prepared according to the reported protocols, and their ^1^H NMR spectra were the same as those in the literature [26,27,28,29].

### 3.2. p-Methylphenyl 2,3-di-O-acetyl-β-D-galactopyranosyl-(1→4)-2,3,6-tri-O-acetyl-1-thio-β-D-glucopyranoside (**4**)

A solution of **3** (51.5 g, 68.96 mmol) in 80% aqueous AcOH was heated at 85 °C using an aluminum block for 3 h. After the reaction was complete, as indicated by the consumption of all starting material on TLC, the solvent was removed under reduced pressure followed by co-evaporation with toluene three times. Thereafter, ethyl acetate (EtOAc) was added, and the solution was washed with NaHCO_3_, water, and brine multiple times. The organic layer was dried over MgSO_4_, filtered, and concentrated under vacuum. The crude product was purified using silica gel column chromatography to afford **4** (59%, 26.8 g) as a white solid. TLC: R_f_ = 0.61 (EtOAc:Hex 80:20). ^1^H NMR (500 MHz, CDCl_3_): δ 7.35 (d, *J* = 8.0 Hz, 2H), 7.09 (d, *J* = 8.0 Hz, 2H), 5.31–5.02 (m, 2H), 4.95–4.79 (m, 2H), 4.61 (d, *J* = 10.1 Hz, 1H, anomeric), 4.53 (dd, *J* = 11.7, 1.9 Hz, 1H), 4.46 (d, *J* = 7.9 Hz, 1H, anomeric), 4.08 (dt, *J* = 11.9, 8.5 Hz, 2H), 3.93–3.72 (m, 3H), 3.68–3.48 (m, 2H), 3.45–3.30 (m, 1H), 2.79 (dd, *J* = 7.1, 4.7 Hz, 1H), 2.33 (s, 3H), 2.09 (s, 3H), 2.07 (s, 3H), 2.05 (s, 6H, 2 × CH_3_), 2.02 (s, 3H). ^13^C NMR (126 MHz, CDCl_3_): δ 170.8, 170.6, 170.4, 169.71, 169.7, 138.7, 133.7, 129.7, 127.8, 101.1, 85.6, 76.7, 76.3, 74.5, 73.6, 70.4, 70.3, 69.8, 67.8, 62.4, 62.1, 21.3, 21.01, 21.0, 20.9, 20.8. HR-ESI-MS *m*/*z*: [M + H_2_O + H]^+^ Calcd for C_29_H_41_O_16_S 677.2115; Found 677.2106.

### 3.3. p-Methylphenyl 2,3-di-O-acetyl-6-O-(p-toluenesulfonyl)-β-D-galactopyranosyl-(1→4)-2,3,6-tri-O-acetyl-1-thio-β-D-glucopyranoside (**5**)

To a solution of **4** (20.5 g, 31.12 mmol) dissolved in pyridine (93.2 mL) was added *p*-TsCl (5.93 g, 31.12 mmol) portionwise under an N_2_ atmosphere in an ice bath. The mixture was stirred at rt for 6 h, at which point the reaction did not show further progress. Next, MeOH was added dropwise to the reaction mixture, and the solvent was removed under vacuo. The residue was extracted with EtOAc and washed twice with brine. The organic layer was dried over MgSO_4_ and concentrated under reduced pressure. Purification of the product by silica gel column chromatography afforded **5** (58%, 14.67 g) as a white waxy solid. TLC: R_f_ = 0.95 (EtOAc:Hex 80:20). ^1^H NMR (400 MHz, CDCl_3_): δ 7.78 (d, *J* = 8.3 Hz, 2H), 7.40–7.35 (m, 4H), 7.10 (d, *J* = 8.0 Hz, 2H), 5.15 (t, *J* = 9.1 Hz, 1H), 5.09 (dd, *J* = 10.2, 7.9 Hz, 1H), 4.92–4.86 (m, 1H), 4.83 (d, *J* = 9.4 Hz, 1H), 4.58 (d, *J* = 10.1 Hz, 1H, anomeric), 4.50 (dd, *J* = 11.9, 1.9 Hz, 1H), 4.41 (d, *J* = 7.9 Hz, 1H, anomeric), 4.24 (dd, *J* = 10.4, 6.5 Hz, 1H), 4.12–4.05 (m, 2H), 4.03 (t, *J* = 4.4 Hz, 1H), 3.76 (t, *J* = 6.4 Hz, 1H), 3.68 (t, *J* = 9.5 Hz, 1H), 3.58 (ddd, *J* = 10.0, 5.3, 1.9 Hz, 1H), 2.47 (s, 3H), 2.33 (s, 3H), 2.10 (s, 3H), 2.08 (s, 3H), 2.06 (s, 3H), 2.01 (s, 3H), 1.90 (s, 3H). ^13^C NMR (101 MHz, CDCl_3_): δ 170.5, 170.2, 170.1, 169.6, 169.5, 145.5, 138.7, 133.7, 132.6, 130.2, 129.7, 128.0, 127.9, 100.8, 85.6, 76.7, 75.9, 74.0, 73.2, 72.3, 70.3, 69.5, 67.1, 66.5, 62.2, 21.8, 21.3, 20.92, 20.91, 20.9, 20.8, 20.7. HR-ESI-MS *m*/*z*: [M + NH_4_]^+^ Calcd for C_36_H_48_NO_17_S_2_ 830.2364; Found 830.2354

### 3.4. p-Methylphenyl 2,3,4-tri-O-acetyl-6-O-(p-toluenesulfonyl)-β-D-galactopyranosyl-(1→4)-2,3,6-tri-O-acetyl-1-thio-β-D-glucopyranoside (**6**)

After **5** (14.5 g, 17.84 mmol) was dissolved in pyridine (90 mL), Ac_2_O (2.53 mL, 26.76 mmol) and DMAP (0.4 g, 3.57 mmol) were added at 0 °C under an N_2_ atmosphere. The mixture was stirred at rt overnight. After the complete consumption of **5** as indicated by TLC, MeOH was added dropwise to quench the reaction. The solvent was evaporated under vacuo. The resulting residue was extracted with EtOAc, and the organic layer was washed three times with H_2_O and brine, dried over MgSO_4_, and finally, concentrated under vaccum. The residue was purified by silica gel column chromatography to afford **6** (96%, 14.6 g) as a white waxy solid. TLC: R_f_ = 0.66 (DCM:MeOH 95:5). ^1^H NMR (400 MHz, CDCl_3_): δ 7.75 (d, *J* = 8.3 Hz, 2H), 7.36 (dd, *J* = 8.1, 1.7 Hz, 4H), 7.10 (d, *J* = 7.9 Hz, 2H), 5.32 (dd, *J* = 3.4, 0.8 Hz, 1H), 5.18 (t, *J* = 9.1 Hz, 1H), 5.04 (dd, *J* = 10.4, 7.9 Hz, 1H), 4.91 (dd, *J* = 10.4, 3.4 Hz, 1H), 4.83 (t, *J* = 9.6 Hz, 1H), 4.59 (d, *J* = 10.1 Hz, 1H, anomeric), 4.50 (dd, *J* = 11.9, 2.0 Hz, 1H), 4.44 (d, *J* = 7.9 Hz, 1H, anomeric), 4.08–3.95 (m, 3H), 3.88 (dd, *J* = 7.5, 6.5 Hz, 1H), 3.70 (t, *J* = 9.5 Hz, 1H), 3.59 (ddd, *J* = 10.0, 5.2, 1.9 Hz, 1H), 2.46 (s, 3H), 2.33 (s, 3H), 2.10 (s, 3H), 2.09 (s, 3H), 2.04 (s, 3H), 2.01 (s, 3H), 1.95 (s, 3H), 1.94 (s, 3H). ^13^C NMR (101 MHz, CDCl_3_): δ 170.4, 170.01, 170.0, 169.8, 169.6, 169.1, 145.6, 138.8, 133.8, 132.3, 130.2, 129.8, 128.1, 127.8, 100.9, 85.6, 76.7, 76.1, 74.0, 70.91, 70.9, 70.4, 69.1, 66.6, 65.5, 62.1, 21.8, 21.3, 21.01, 21.0, 20.9, 20.7, 20.61, 20.6. HR-ESI-MS *m*/*z*: [M + HCOO]^−^ Calcd for C_39_H_47_O_20_S_2_ 899.2103; Found 899.2074.

### 3.5. 2,3,4-Tri-O-acetyl-6-O-(p-toluenesulfonyl)-β-D-galactopyranosyl-(1→4)-2,3,6-tri-O-acetyl-D-glucopyranose (**7**)

To a solution of **6** (8.0 g, 9.36 mmol) dissolved in acetone (160 mL) and H_2_O (40 mL) was added NBS (3.33 g, 18.72 mmol) at 0 °C. The mixture was stirred at rt for 1 h, while the reaction was monitored by TLC. Once no further progress was observed with TLC, the reaction was quenched by adding solutions of Na_2_S_2_O_3_ and NaHCO_3_ dropwise. Acetone was removed under vacuum and then saturated aq. NaHCO_3_ solution was added to the residue. The mixture was extracted with DCM three times. The organic layers were combined, dried over MgSO_4_, and concentrated under vacuum. The residue was purified by silica gel column chromatography to afford **7** (63%, 4.40 g) as a white solid, along with recovery of **6** (34%, 2.70 g). TLC: R_f_ = 0.25 (EtOAc:Hex 60:40). ^1^H NMR (400 MHz, CDCl_3_): δ 7.77 (d, *J* = 8.3 Hz, 2H), 7.37 (d, *J* = 8.4 Hz, 2H), 5.54 (t, *J* = 9.7 Hz, 1H), 5.38 (t, *J* = 3.4 Hz, 1H), 5.32 (d, *J* = 2.5 Hz, 1H), 5.09 (dd, *J* = 10.4, 7.9 Hz, 1H), 4.92 (dd, *J* = 10.4, 3.4 Hz, 1H), 4.83 (dd, *J* = 10.1, 3.4 Hz, 1H, anomeric), 4.53 (d, *J* = 7.9 Hz, 1H, anomeric), 4.48 (ddd, *J* = 10.4, 7.3, 2.3 Hz, 2H), 4.23 (ddd, *J* = 10.1, 4.1, 1.9 Hz, 1H), 4.12 (dd, *J* = 12.0, 4.1 Hz, 1H), 4.07 (dd, *J* = 10.6, 4.2 Hz, 2H), 3.96 (t, *J* = 3.3 Hz, 1H), 3.94–3.90 (m, 1H), 3.79 (t, *J* = 9.7 Hz, 1H), 2.13 (s, 3H), 2.09 (s, 3H), 2.06 (s, 3H), 2.03 (s, 3H), 2.01 (s, 3H), 1.94 (s, 3H). ^13^C NMR (101 MHz, CDCl_3_): δ 170.7, 170.4, 170.1, 169.8, 169.2, 169.1, 132.3, 130.2, 129.8, 128.2, 100.6, 90.3, 77.4, 76.3, 71.6, 71.0, 69.3, 69.1, 68.3, 66.8, 66.0, 62.0, 21.8, 21.1, 21.0, 20.9, 20.8, 20.7, 20.6. HR-ESI-MS *m*/*z*: [M-H_2_O + H]^+^ Calcd for C_31_H_39_O_18_S 731.1857; Found: 731.1833.

### 3.6. 2,3,4-Tri-O-acetyl-6-O-(p-toluenesulfonyl)-β-D-galactopyranosyl-(1→4)-2,3,6-tri-O-acetyl-D-glucopyranosyl trichloroimidate (**8**)

To a solution of **7** (990 mg, 1.322 mmol) in dry DCM (9.5 mL) were added DBU (0.132 mmol, 20 µL) and trichloroacetonitrile (2.12 mL, 21.15 mmol) dropwise at 0 °C under an N_2_ atmosphere. The mixture was stirred at rt for 3 h. After the reaction was completed as indicated by TLC, the solvent was removed under reduced pressure, and the residue was purified by flash column chromatography using Et_3_N-deactivated silica to give **8** (55%, 649 mg). TLC: R_f_ = 0.45 (EtOAc:Hex 50:50). ^1^H NMR (400 MHz, CDCl_3_): δ 8.66 (s, 1H, NH), 7.77 (d, *J* = 8.2 Hz, 2H), 7.38 (d, *J* = 8.1 Hz, 2H), 6.49 (d, *J* = 3.7 Hz, 1H, anomeric), 5.55 (t, *J* = 9.6 Hz, 1H), 5.33 (d, *J* = 3.3 Hz, 1H), 5.13–5.08 (m, 1H), 5.05 (dd, *J* = 10.2, 3.9 Hz, 1H), 4.93 (dd, *J* = 10.4, 3.4 Hz, 1H), 4.51 (d, *J* = 8.0 Hz, 1H, anomeric), 4.49–4.44 (m, 1H), 4.14–4.05 (m, 1H), 3.99 (dd, *J* = 10.0, 6.6 Hz, 1H), 3.95–3.81 (m, 1H), 2.11 (s, 3H), 2.06 (s, 3H), 2.03 (s, 6H, 2 × CH_3_), 2.02 (s, 3H), 1.95 (s, 3H). ^13^C NMR (101 MHz, CDCl_3_): δ 170.4, 170.2, 170.1, 170.0, 169.5, 169.2, 161.1, 145.7, 132.3, 130.2, 128.2, 101.3, 93.0, 77.4, 76.0, 71.1, 71.0, 70.2, 69.7, 69.1, 66.7, 65.6, 61.6, 21.8, 21.2, 21.05, 21.0, 20.8, 20.7, 20.6.

### 3.7. (2S,3R)-2-(tert-Butoxycarbonyl)amino-3-pivaloyloxy-pent-4-en-1-yl 2,3,4-tri-O-acetyl-6-O-(p-toluenesulfonyl)-β-D-galactopyranosyl-(1→4)-2,3,6-tri-O-acetyl-D-glucopyranoside (**10**)

The mixture of glycosyl donor **8** (600 mg, 0.671 mmol), acceptor **9** (168 mg, 0.559 mmol), and flame-dried MS 4Å (1.5 g) in dry DCM (12 mL) was stirred at rt for 15 min under an N_2_ atmosphere and then cooled to -78 °C. Thereafter, TMSOTf (4.0 µL, 22.4 µmol) was added dropwise followed by stirring at the same temperature (−78 °C) for 15 min. The mixture was gradually warmed to rt and stirred for another 45 min. When TLS indicated the complete consumption of **9**, the reaction was quenched with Et_3_N at 0 °C, and the mixture was filtered through a Celite pad. The filtrate was washed with saturated NaHCO_3_solution, and the organic layer was dried over MgSO_4_ and concentrated under vacuo. Silica gel column chromatography of the residue afforded **10** (53% yield, 306 mg) as a white solid. TLC: R_f_ = 0.50 (EtOAc:Hex 50:50).^1^H NMR (600 MHz, CDCl_3_): δ 7.77 (d, *J* = 8.3 Hz, 2H), 7.38 (d, *J* = 8.1 Hz, 2H), 5.83–5.73 (m, 1H, =CH), 5.32 (dd, *J* = 11.2, 5.1 Hz, 2H), 5.29 (d, *J* = 1.2 Hz, 1H), 5.26 (d, *J* = 10.7 Hz, 1H), 5.17 (t, *J* = 9.3 Hz, 1H), 5.06 (dd, *J* = 10.3, 7.9 Hz, 1H), 4.92 (dd, *J* = 10.4, 3.4 Hz, 1H), 4.88 (dd, *J* = 9.4, 8.0 Hz, 1H), 4.70 (d, *J* = 8.9 Hz, 1H, -NH), 4.46 (d, *J* = 7.8 Hz, 1H, anomeric), 4.45–4.44 (m, 1H), 4.43 (d, *J* = 7.8 Hz, 1H, anomeric), 4.08–3.97 (m, 4H), 3.94 (dd, *J* = 10.0, 3.4 Hz, 1H), 3.90 (t, *J* = 6.5 Hz, 1H), 3.77 (t, *J* = 9.5 Hz, 1H), 3.63–3.57 (m, 1H), 3.51 (dd, *J* = 10.1, 4.9 Hz, 1H), 2.46 (s, 1H), 2.11 (s, 1H), 2.06 (s, 1H), 2.04 (s, 1H), 2.02 (s, 1H), 1.99 (s, 1H), 1.94 (s, 1H), 1.42 (s, 9H), 1.19 (s, 9H). ^13^C NMR (151 MHz, CDCl_3_): δ 177.1, 170.5, 170.01, 170.0, 169.9, 169.8, 169.7, 169.2, 155.3, 145.7, 132.4, 130.2, 128.2, 118.7, 100.91, 100.9, 79.8, 76.1, 73.3, 72.8, 72.6, 71.8, 70.91, 70.9, 69.1, 68.5, 66.7, 65.6, 62.1, 60.5, 52.2, 39.0, 28.5, 27.2, 21.8, 21.2, 21.0, 20.9, 20.71, 20.7, 20.6. HR-ESI-MS *m*/*z*: [M + HCOO]^−^ Calcd for C_47_H_66_NO_25_S 1076.3645; Found 1076.3657.

### 3.8. (2S,3R,E)-2-(tert-Butoxycarbonyl)amino-3-pivaloyloxy-octadec-4-en-1-yl 2,3,4-tri-O-acetyl-6-O-(p-toluenesulfonyl)-β-D-galactopyranosyl-(1→4)-2,3,6-tri-O-acetyl-D-glucopyranoside (**12a**)

To an N_2_-bubbled mixture of **10** (150 mg, 0.145 mmol) and 1-pentadecene **11a** (183.5 mg, 0.237 mL, 0.872 mmol) in dry DCM (28 mL) was added 2nd generation Hoveyda−Grubbs catalyst (9 mg, 10 mol%). The reaction mixture was refluxed at 40 °C for 6 d. Each day, another batch of **11a** (183.5 mg, 0.237 mL, 0.873 mmol) and the catalyst (4.53 mg, 5 mol%) was added to the mixture. Upon the completion of reaction, DMSO (2 drops) was added, and the mixture was stirred at rt for 40 min. The solvent was removed under vacuo, and the residue was purified by silica gel column chromatography to give **12a** (83%, 146.5 mg) as a brownish solid. TLC: R_f_ = 0.83 (EtOAc:Hex 80:20). ^1^H NMR (400 MHz, CDCl_3_): δ 7.78 (d, *J* = 8.3 Hz, 2H), 7.39 (d, *J* = 8.0 Hz, 2H), 5.75 (dt, *J* = 14.6, 7.6 Hz, 1H, =CH-), 5.40–5.30 (m, 2H, =CH- and 4′-H), 5.18 (dd, *J* = 17.4, 8.2 Hz, 2H), 5.06 (dd, *J* = 10.4, 7.9 Hz, 1H), 4.90 (ddd, *J* = 17.3, 9.9, 5.7 Hz, 2H), 4.67 (d, *J* = 9.2 Hz, 1H, NH), 4.46 (d, *J* = 7.8 Hz, 1H, anomeric), 4.43 (d, *J* = 7.7 Hz, 1H, anomeric), 4.10–3.96 (m, 4H), 3.91 (dd, *J* = 13.5, 7.8 Hz, 2H), 3.77 (t, *J* = 9.4 Hz, 1H), 3.59 (ddd, *J* = 9.8, 4.7, 1.7 Hz, 1H), 3.49 (dd, *J* = 9.7, 4.4 Hz, 1H), 2.47 (s, 3H), 2.11 (s, 3H), 2.07 (s, 3H), 2.06 (s, 3H), 2.03 (s, 3H), 1.99 (s, 3H), 1.95 (s, 3H), 1.42 (s, 9H), 1.30–1.21 (m, 24H), 1.17 (s, 9H), 0.88 (t, *J* = 6.8 Hz, 3H). ^13^C NMR (101 MHz, CDCl_3_): δ 177.0, 171.3, 170.5, 170.01, 170.0, 169.9, 169.8, 169.2, 145.6, 137.0, 132.4, 130.2, 128.2, 124.6, 100.91, 100.9, 77.4, 76.1, 73.3, 72.71, 72.7, 71.8, 70.9, 69.1, 68.61, 68.6, 66.6, 65.5, 62.1, 60.5, 52.3, 38.9, 32.5, 32.1, 29.81, 29.8, 29.7, 29.5, 29.3, 29.1, 28.5, 27.2, 22.8, 21.8, 21.2, 21.0, 20.9, 20.8, 20.71, 20.7, 20.6, 14.31, 14.3. HR-ESI-MS *m*/*z*: [M + HCOO]^−^ Calcd for C_60_H_92_NO_25_S 1258.5680; found: 1258.5686

### 3.9. (2S,3R,E)-2-(tert-Butoxycarbonyl)amino-3-pivaloyloxy-pentacos-4-en-1-yl 2,3,4-tri-O-acetyl-6-O-(p-toluenesulfonyl)-β-D-galactopyranosyl-(1→4)-2,3,6-tri-O-acetyl-D-glucopyranoside (**12b**)

Compound **12b** (80%, 152.6 mg) was prepared as a blackish brown solid from **11b** by the same method and conditions utilized to prepare **12a**. TLC: R_f_ = 0.92 (EtOAc:Hex 80:20). ^1^H NMR (600 MHz, CDCl_3_): δ 7.78 (d, *J* = 8.3 Hz, 2H), 7.38 (d, *J* = 8.1 Hz, 2H), 5.75 (dt, *J* = 14.6, 7.6 Hz, 1H, =CH-), 5.40–5.30 (m, 2H, =CH- and 4′-H), 5.18 (dd, *J* = 17.3, 8.1 Hz, 2H), 5.06 (dd, *J* = 10.4, 7.9 Hz, 1H), 4.89 (ddd, *J* = 17.3, 9.9, 5.7 Hz, 2H), 4.66 (d, *J* = 8.9 Hz, 1H, NH), 4.46 (d, *J* = 7.8 Hz, 1H, anomeric), 4.45–4.44 (m, 1H), 4.43 (d, *J* = 7.6 Hz, 1H, anomeric), 4.05 (dd, *J* = 11.1, 5.0 Hz, 2H), 3.99 (dd, *J* = 10.2, 6.6 Hz, 2H), 3.91 (dd, *J* = 13.4, 7.5 Hz, 2H), 3.77 (t, *J* = 9.4 Hz, 1H), 3.59 (ddd, *J* = 9.6, 4.6, 1.5 Hz, 1H), 3.48 (dd, *J* = 9.7, 4.4 Hz, 1H), 2.47 (s, 3H), 2.10 (s, 3H), 2.06 (s, 6H), 2.02 (s, 3H), 1.99 (s, 3H), 1.94 (s, 3H), 1.42 (s, 9H), 1.36–1.20 (m, 38H), 1.17 (s, 9H), 0.88 (t, *J* = 6.8 Hz, 3H). ^13^C NMR (151 MHz, CDCl_3_): δ 177.0, 170.5, 170.01, 170.0, 169.9, 169.81, 169.8, 169.2, 145.6, 137.0, 132.4, 130.2, 128.1, 124.6, 100.91, 100.9, 76.1, 73.3, 72.71, 72.7, 71.8, 70.9, 69.0, 68.61, 68.6, 66.6, 65.5, 62.1, 52.3, 38.9, 38.4, 35.9, 32.5, 32.1, 29.82, 29.81, 29.8, 29.6, 29.5, 29.3, 29.1, 28.5, 27.21, 27.2, 22.8, 21.8, 21.0, 20.9, 20.8, 20.7, 20.61, 20.6, 14.3. HR-ESI-MS *m*/*z*: [M + CH_3_OH–H]^−^ Calcd for C_67_H_108_NO_24_S 1342.6982; Found: 1342.7032.

### 3.10. (2S,3R,E)-2-Octadecanamido-3-pivaloyloxy-octadec-4-en-1-yl 2,3,4-tri-O-acetyl-6-O-(p-toluenesulfonyl)-β-D-galactopyranosyl-(1→4)-2,3,6-tri-O-acetyl-D-glucopyranoside (**13a**)

To a stirred solution of **12a** (59 mg, 48.58 µmol) in dry DCM (10 mL) was added TFA (1.0 mL) dropwise over 5 min in an ice bath. After the ice bath was removed, the mixture was stirred at rt for 2.5 h. Toluene was added, and the organic layer was washed with NaHCO_3_ solution three times, dried over Na_2_SO_4_, and filtered. The filtrate was concentrated under reduced pressure, affording the free amine derivative. To the solution of stearic acid (20.2 mg, 70.9 µmol) dissolved in DCM (8.7 mL) in an ice-bath was added EDC (13.6 mg, 70.9 µmol) under an N_2_ atmosphere. After the mixture was stirred for 15 min, the above-obtained free amine (39.5 mg, 35.45 µmol) in DCM (4.3 mL) was added dropwise followed by the addition of DMAP (0.8 mg, 7.1 µmol). The reaction mixture was stirred at rt for 24 h. NaHCO_3_ solution was added dropwise to the mixture at 0 °C, and the aqueous layer was extracted with DCM three times. The combined organic layer was concentrated under vacuo, and the residue was purified using silica gel column chromatography to afford **13a** (65%, 43.6 mg) as an off-white solid. TLC: R_f_ = 0.55 (EtOAc:Hex 50:50). ^1^H NMR (600 MHz, CDCl_3_): δ 7.78 (d, *J* = 8.3 Hz, 2H), 7.38 (d, *J* = 8.1 Hz, 2H), 5.75 (dt, *J* = 14.8, 7.4 Hz, 1H, =CH-), 5.61 (d, *J* = 9.3 Hz, 1H, −NHCO−), 5.36–5.33 (m, 2H, =CH- and 4′-H), 5.23 (t, *J* = 7.1 Hz, 1H), 5.19 (t, *J* = 9.3 Hz, 1H), 5.06 (dd, *J* = 10.3, 8.0 Hz, 1H), 4.92 (dd, *J* = 10.4, 3.4 Hz, 1H), 4.86 (dd, *J* = 9.4, 7.9 Hz, 1H), 4.47 (d, *J* = 7.9 Hz, 1H, anomeric), 4.45–4.44 (m, 1H), 4.43 (d, *J* = 7.8 Hz, 1H, anomeric), 4.33 (ddd, *J* = 11.3, 8.7, 4.1 Hz, 1H), 4.06 (ddd, *J* = 16.9, 11.1, 5.6 Hz, 1H), 3.99 (dd, *J* = 10.2, 6.5 Hz, 1H), 3.90 (dd, *J* = 12.6, 5.6 Hz, 1H), 3.78 (t, *J* = 9.5 Hz, 1H), 3.60 (ddd, *J* = 9.9, 4.7, 2.0 Hz, 1H), 3.52 (dd, *J* = 10.0, 4.4 Hz, 1H), 2.47 (s, 3H), 2.11 (s, 3H), 2.06 (s, 3H), 2.04 (s, 3H), 2.03 (s, 3H), 2.00 (s, 3H), 1.95 (s, 3H), 1.59–1.56 (m, 2H), 1.25 (s, 56H), 1.17 (s, 9H), 0.88 (t, *J* = 7.0 Hz, 6H, 2 × Me). ^13^C NMR (151 MHz, CDCl_3_): δ 177.1, 172.7, 170.5, 170.01, 170.0, 169.81, 169.8, 169.2, 145.7, 137.1, 132.4, 130.2, 128.2, 124.9, 100.9, 100.6, 76.0, 73.2, 72.8, 72.5, 72.0, 71.0, 70.9, 69.1, 67.8, 66.7, 65.6, 62.0, 50.6, 38.9, 37.0, 32.5, 32.1, 29.9, 29.8, 29.71, 29.7, 29.6, 29.5, 29.4, 29.2, 27.2, 25.9, 22.9, 21.8, 21.0, 20.91, 20.9, 20.8, 20.7, 20.6, 14.3. HR-ESI-MS *m*/*z*: [M + Na]^+^ Calcd for C_72_H_117_NNaO_22_S: 1402.7686; Found: 1402.7698.

### 3.11. (2S,3R,E)-2-Pentacosanamido-3-pivaloyloxy-octadec-4-en-1-yl 2,3,4-tri-O-acetyl-6-O-(p-toluenesulfonyl)-β-D-galactopyranosyl-(1→4)-2,3,6-tri-O-acetyl-D-glucopyranoside (**13b**)

Compound **13b** (64%, 44.4 mg) was prepared as a white solid from **12b** (57 mg, 46.9 µmol) by the same method and conditions employed to prepare **13a**. TLC: R*_f_* = 0.5 (EtOAc:Hex 50:50). ^1^H NMR (600 MHz, CDCl_3_): δ 7.78 (d, *J* = 8.3 Hz, 2H), 7.38 (d, *J* = 8.1 Hz, 2H), 5.75 (dt, *J* = 14.7, 7.3 Hz, 1H, =CH-), 5.61 (d, *J* = 9.2 Hz, 1H, -NHCO-), 5.36–5.33 (m, 2H, =CH- and 4′-H), 5.23 (t, *J* = 7.1 Hz, 1H), 5.18 (t, *J* = 9.3 Hz, 1H), 5.06 (dd, *J* = 10.3, 8.0 Hz, 1H), 4.92 (dd, *J* = 10.4, 3.4 Hz, 1H), 4.86 (dd, *J* = 9.4, 7.9 Hz, 1H), 4.47 (d, *J* = 7.9 Hz, 1H, anomeric), 4.45 (d, *J* = 1.6 Hz, 1H), 4.43 (d, *J* = 7.8 Hz, 1H, anomeric), 4.33 (ddd, *J* = 11.3, 8.8, 4.1 Hz, 1H), 4.06 (ddd, *J* = 16.8, 11.1, 5.6 Hz, 2H), 3.99 (dd, *J* = 10.2, 6.6 Hz, 1H), 3.90 (dd, *J* = 12.0, 5.5 Hz, 2H), 3.78 (t, *J* = 9.5 Hz, 1H), 3.60 (ddd, *J* = 9.8, 4.7, 1.9 Hz, 1H), 3.52 (dd, *J* = 10.0, 4.4 Hz, 1H), 2.47 (s, 3H), 2.11 (s, 3H), 2.06 (s, 3H), 2.04 (s, 3H), 2.03 (s, 3H), 1.99 (s, 3H), 1.95 (s, 3H), 1.59–1.56 (m, 2H), 1.32–1.22 (m, 66H), 1.17 (s, 9H), 0.88 (t, *J* = 7.0 Hz, 6H, 2 × Me). ^13^C NMR (151 MHz, CDCl_3_): δ 177.1, 172.7, 170.5, 170.01, 170.0, 169.81, 169.8, 169.2, 145.6, 137.1, 132.4, 130.2, 128.2, 124.9, 100.9, 100.6, 76.0, 73.2, 72.8, 72.5, 72.0, 71.0, 70.9, 69.1, 67.8, 66.7, 65.6, 62.1, 50.6, 38.9, 37.0, 32.5, 32.1, 29.91, 29.9, 29.8, 29.71, 29.7, 29.6, 29.51, 29.5, 29.4, 29.2, 27.2, 25.9, 22.9, 21.8, 21.0, 20.91, 20.9, 20.8, 20.7, 20.6, 14.3. HR-ESI-MS *m*/*z*: [M + HCOO]^−^ Calcd for C_80_H_132_NO_24_S 1522.8860; found: 1522.8896.

### 3.12. (2S,3R,E)-2-Octadecanamido-3-pivaloyloxy-pentacos-4-en-1-yl 2,3,4-tri-O-acetyl-6-O-(p-toluenesulfonyl)-β-D-galactopyranosyl-(1→4)-2,3,6-tri-O-acetyl-D-glucopyranoside (**13c**)

Compound **13c** (63%, 36.2 mg) as a white solid was prepared from **12c** (51 mg, 38.9 µmol) by the same method and conditions employed to prepare **13a**. TLC: R_f_ = 0.5 (EtOAc:Hex 50:50). ^1^H NMR (600 MHz, CDCl_3_): δ 7.78 (d, *J* = 8.3 Hz, 2H), 7.38 (d, *J* = 8.0 Hz, 2H), 5.75 (dt, *J* = 14.7, 7.3 Hz, 1H, =CH-), 5.61 (d, *J* = 9.2 Hz, 1H, -NHCO-), 5.36–5.33 (m, 2H, =CH- and 4′-H), 5.23 (t, *J* = 7.2 Hz, 1H), 5.18 (t, *J* = 9.3 Hz, 1H), 5.06 (dd, *J* = 10.4, 7.9 Hz, 1H), 4.92 (dd, *J* = 10.4, 3.4 Hz, 1H), 4.86 (dd, *J* = 9.4, 7.9 Hz, 1H), 4.47 (d, *J* = 7.9 Hz, 1H, anomeric), 4.45 (d, *J* = 1.6 Hz, 1H), 4.43 (d, *J* = 7.8 Hz, 1H, anomeric), 4.33 (ddd, *J* = 11.3, 8.8, 4.2 Hz, 1H), 4.06 (ddd, *J* = 16.9, 11.1, 5.6 Hz, 2H), 3.99 (dd, *J* = 10.2, 6.6 Hz, 1H), 3.93–3.88 (m, 2H), 3.78 (t, *J* = 9.5 Hz, 1H), 3.60 (ddd, *J* = 9.9, 4.7, 2.0 Hz, 1H), 3.52 (dd, *J* = 10.0, 4.4 Hz, 1H), 2.47 (s, 3H), 2.11 (s, 3H), 2.06 (s, 3H), 2.04 (s, 3H), 2.03 (s, 3H), 1.99 (s, 3H), 1.95 (s, 3H), 1.57 (b, 2H), 1.35–1.22 (m, 70H), 1.17 (s, 9H), 0.88 (t, *J* = 7.0 Hz, 6H, 2 × Me). ^13^C NMR (151 MHz, CDCl_3_): δ 177.1, 172.7, 170.5, 170.01, 170.0, 169.81, 169.8, 169.2, 145.6, 137.2, 132.4, 130.2, 128.2, 124.9, 100.9, 100.6, 76.0, 73.1, 72.8, 72.5, 72.0, 71.0, 70.9, 69.1, 67.8, 66.7, 65.6, 62.0, 50.6, 38.9, 37.0, 32.5, 32.1, 29.9, 29.8, 29.71, 29.7, 29.6, 29.5, 29.4, 29.2, 27.2, 25.9, 22.9, 21.8, 21.0, 20.91, 20.9, 20.8, 20.7, 20.6, 14.3. HR-ESI-MS *m*/*z*: [M + CH_3_OH-H]^−^ Calcd for C_80_H_134_NO_23_S 1508.9067; found: 1508.9098.

### 3.13. (2S,3R,E)-2-Pentacosanamido-3-pivaloyloxy-pentacos-4-en-1-yl 2,3,4-tri-O-acetyl-6-O-(p-toluenesulfonyl)-β-D-galactopyranosyl-(1→4)-2,3,6-tri-O-acetyl-D-glucopyranoside (**13d**)

Compound **13d** (63%, 37.8 mg) was prepared as a white solid from **12d** (50 mg, 38.1 µmol) by the same method and conditions employed to prepare **13a**. TLC: R_f_ = 0.5 (EtOAc:Hex 50:50). ^1^H NMR (600 MHz, CDCl_3_): δ 7.77 (d, *J* = 8.3 Hz, 2H), 7.38 (d, *J* = 8.1 Hz, 2H), 5.75 (dt, *J* = 14.8, 7.3 Hz, 1H, =CH-), 5.61 (d, *J* = 9.2 Hz, 1H, -NHCO-), 5.36–5.33 (m, 2H, =CH- and 4′-H), 5.23 (t, *J* = 7.1 Hz, 1H), 5.18 (t, *J* = 9.3 Hz, 1H), 5.06 (dd, *J* = 10.3, 8.0 Hz, 1H), 4.92 (dd, *J* = 10.4, 3.4 Hz, 1H), 4.86 (dd, *J* = 9.4, 7.9 Hz, 1H), 4.47 (d, *J* = 7.9 Hz, 1H, anomeric), 4.45 (d, J = 1.4 Hz, 1H), 4.43 (d, *J* = 7.8 Hz, 1H, anomeric), 4.33 (ddd, *J* = 11.3, 8.7, 4.1 Hz, 1H), 4.05 (ddd, *J* = 16.9, 11.1, 5.6 Hz, 2H), 3.99 (dd, *J* = 10.2, 6.5 Hz, 1H), 3.90 (dd, *J* = 11.9, 5.5 Hz, 2H), 3.78 (t, *J* = 9.5 Hz, 1H), 3.60 (ddd, *J* = 9.9, 4.7, 2.0 Hz, 1H), 3.52 (dd, *J* = 10.0, 4.4 Hz, 1H), 2.47 (s, 3H), 2.11 (s, 3H), 2.06 (s, 3H), 2.04 (s, 3H), 2.03 (s, 3H), 1.99 (s, 3H), 1.95 (s, 3H), 1.64 (dd, *J* = 14.7, 7.2 Hz, 2H), 1.59–1.56 (m, 2H), 1.34–1.23 (m, 80H), 1.17 (s, 9H), 0.88 (t, *J* = 7.0 Hz, 6H, 2 × Me). ^13^C NMR (151 MHz, CDCl_3_): δ 177.1, 172.7, 170.5, 170.01, 170.0, 169.81, 169.8, 169.2, 145.6, 137.2, 132.4, 130.2, 128.2, 124.9, 100.9, 100.6, 76.0, 73.1, 72.8, 72.5, 72.0, 71.0, 70.9, 69.1, 67.8, 66.7, 65.6, 62.0, 50.6, 38.9, 37.0, 32.5, 32.1, 29.91, 29.9, 29.8, 29.71, 29.7, 29.6, 29.5, 29.4, 29.2, 27.2, 25.9, 22.9, 21.8, 21.0, 20.91, 20.9, 20.8, 20.7, 20.6, 14.3. HR-ESI-MS *m*/*z*: [M-H]^−^ Calcd for C_86_H_144_NO_22_S 1574.9901; found: 1574.9834.

### 3.14. (2S,3R,E)-2-Octadecanamido-3-pivaloyloxy-octadec-4-en-1-yl 2,3,4-tri-O-acetyl-6-azido-6-deoxy-β-D-galactopyranosyl-(1→4)-2,3,6-tri-O-acetyl-D-glucopyranoside (**14a**)

To a solution of **13a** (30 mg, 21.7 µmol) in dry DMF (6.0 mL) was added NaN_3_ (28 mg, 434.5 µmol), and the mixture was heated at 86 °C for 2 d. When the starting material **13a** disappeared in TLC, the reaction was allowed to cool to rt. The mixture was diluted with EtOAc and cooled at 0 °C. After cold water was added, the mixture was extracted with EtOAc three times. The combined organic layers were washed with brine, filtered, dried over Na_2_SO_4_, and condensed under vacuum. The residue was purified through silica gel column chromatography to afford **14a** as a white solid (80%, 21.8 mg). TLC: R_f_ = 0.45 (EtOAc:Hex 40:60). ^1^H NMR (600 MHz, CDCl_3_): δ 5.75 (dt, *J* = 14.8, 7.4 Hz, 1H, =CH-), 5.62 (d, *J* = 9.2 Hz, 1H, −NHCO−), 5.38–5.30 (m, 2H, =CH- and 4′-H), 5.25–5.17 (m, 2H), 5.09 (dd, *J* = 10.4, 7.9 Hz, 1H), 4.96 (dd, *J* = 10.4, 3.5 Hz, 1H), 4.88 (dd, *J* = 9.5, 7.8 Hz, 1H), 4.49–4.44 (m, 1H), 4.50 (d, *J* = 7.9 Hz, 1H, anomeric), 4.42 (d, *J* = 7.7 Hz, 1H, anomeric), 4.32 (ddd, *J* = 11.4, 8.6, 4.1 Hz, 1H), 4.05 (dd, *J* = 12.0, 4.9 Hz, 1H), 3.90 (dd, *J* = 10.0, 3.7 Hz, 1H), 3.85 (t, *J* = 9.5 Hz, 1H), 3.72 (t, *J* = 6.7 Hz, 1H), 3.60 (ddd, *J* = 9.9, 4.7, 2.0 Hz, 1H), 3.52 (dd, *J* = 10.0, 4.4 Hz, 1H), 3.47 (dd, *J* = 12.8, 7.3 Hz, 1H), 3.26 (dd, *J* = 12.8, 5.6 Hz, 1H), 2.17 (s, 3H), 2.12 (s, 3H), 2.07 (s, 3H), 2.04 (s, 3H), 2.04 (s, 3H), 1.97 (s, 3H), 1.59–1.56 (b, 2H), 1.35–1.22 (m, 56H), 1.17 (s, 9H), 0.88 (t, *J* = 7.0 Hz, 6H, 2 × Me). ^13^C NMR (151 MHz, CDCl_3_): δ 177.1, 172.7, 170.5, 170.2, 170.1, 169.81, 169.8, 169.2, 137.2, 125.0, 100.8, 100.7, 75.4, 73.2, 72.9, 72.5, 72.4, 72.0, 71.1, 69.3, 69.2, 67.8, 67.6, 62.0, 50.6, 50.4, 38.9, 37.0, 32.5, 32.1, 29.9, 29.8, 29.71, 29.7, 29.6, 29.5, 29.4, 29.2, 27.2, 25.9, 22.9, 21.1, 21.0, 20.9, 20.8, 20.7, 14.3. HR-ESI-MS *m*/*z*: [M + HCOO]^−^ Calcd for C_66_H_111_N_4_O_21_: 1295.7741; Found: 1295.7753.

### 3.15. (2S,3R,E)-2-Pentacosanamido-3-pivaloyloxy-octadec-4-en-1-yl 2,3,4-tri-O-acetyl-6-azido-6-deoxy-β-D-galactopyranosyl-(1→4)-2,3,6-tri-O-acetyl-D-glucopyranoside (**14b**)

Compound **14b** (76%, 20.8 mg) was prepared as a white solid from **13b** (30 mg, 20.3 µmol) by the same method and conditions employed to prepare **14a**. TLC: R_f_ = 0.65 (EtOAc:Hex 40:60). ^1^H NMR (600 MHz, CDCl_3_): δ 5.75 (dt, *J* = 14.7, 7.4 Hz, 1H, =CH-), 5.61 (d, *J* = 9.2 Hz, 1H, -NHCO-), 5.37–5.31 (m, 2H, =CH- and 4′-H), 5.21 (dt, *J* = 15.5, 8.3 Hz, 2H), 5.09 (dd, *J* = 10.4, 7.9 Hz, 1H), 4.96 (dd, *J* = 10.4, 3.5 Hz, 1H), 4.88 (dd, *J* = 9.5, 7.8 Hz, 1H), 4.51 (d, *J* = 7.8 Hz, 1H, anomeric), 4.48 (d, *J* = 1.7 Hz, 1H), 4.42 (d, *J* = 7.7 Hz, 1H, anomeric), 4.32 (ddd, *J* = 11.4, 8.4, 4.1 Hz, 1H), 4.05 (dd, *J* = 12.0, 4.9 Hz, 1H), 3.91 (dd, *J* = 9.9, 3.7 Hz, 1H), 3.85 (t, *J* = 9.5 Hz, 1H), 3.72 (t, *J* = 6.6 Hz, 1H), 3.60 (ddd, *J* = 9.9, 4.7, 2.0 Hz, 1H), 3.52 (dd, *J* = 10.0, 4.4 Hz, 1H), 3.47 (dd, *J* = 12.8, 7.3 Hz, 1H), 3.26 (dd, *J* = 12.8, 5.6 Hz, 1H), 2.17 (s, 3H), 2.12 (s, 3H), 2.07 (s, 3H), 2.04 (s, 3H), 2.04 (s, 3H), 1.97 (s, 3H), 1.59–1.56 (b, 2H), 1.37–1.21 (m, 70H), 1.17 (s, 9H), 0.88 (t, *J* = 7.0 Hz, 6H, 2 × Me). ^13^C NMR (151 MHz, CDCl_3_): δ 177.1, 172.7, 170.4, 170.2, 170.1, 169.81, 169.8, 169.2, 137.2, 125.0, 100.8, 100.7, 75.4, 73.2, 72.9, 72.5, 72.4, 72.0, 71.1, 69.2, 67.8, 67.6, 62.4, 62.2, 50.6, 50.4, 38.9, 37.0, 32.5, 32.1, 29.9, 29.8, 29.71, 29.7, 29.6, 29.5, 29.4, 29.2, 27.2, 25.9, 22.9, 21.1, 21.0, 20.9, 20.8, 20.7, 14.3. HR-ESI-MS *m*/*z*: [M + CH_3_OH-H]^-^ Calcd for C_73_H_127_N_4_O_20_ 1379.9044; Found: 1379.9058.

### 3.16. (2S,3R,E)-2-Octadecanamido-3-pivaloyloxy-pentacos-4-en-1-yl 2,3,4-tri-O-acetyl-6-azido-6-deoxy-β-D-galactopyranosyl-(1→4)-2,3,6-tri-O-acetyl-D-glucopyranoside (**14c**)

Compound **14c** (69%, 18.9 mg) was prepared as a white solid from **13c** (30 mg, 20.3 µmol) by the same method and conditions employed to prepare **14a**. TLC: R_f_ = 0.45 (EtOAc:Hex 40:60). ^1^H NMR (600 MHz, CDCl_3_) δ 5.75 (dt, *J* = 14.7, 7.4 Hz, 1H, =CH-), 5.62 (d, *J* = 9.3 Hz, 1H, -NHCO-), 5.35–5.32 (m, 2H, =CH- and 4′-H), 5.24–5.16 (m, 2H), 5.09 (dd, *J* = 10.4, 7.9 Hz, 1H), 4.96 (dd, *J* = 10.4, 3.5 Hz, 1H), 4.87 (dd, *J* = 9.5, 7.8 Hz, 1H), 4.50 (d, *J* = 7.8 Hz, 1H, anomeric), 4.48 (d, *J* = 1.8 Hz, 1H), 4.42 (d, *J* = 7.7 Hz, 1H, anomeric), 4.32 (ddd, *J* = 11.4, 8.4, 4.1 Hz, 1H), 4.05 (dd, *J* = 12.0, 4.9 Hz, 1H), 3.90 (dd, *J* = 10.0, 3.7 Hz, 1H), 3.85 (t, *J* = 9.5 Hz, 1H), 3.72 (t, *J* = 6.6 Hz, 1H), 3.60 (ddd, *J* = 9.8, 4.7, 2.0 Hz, 1H), 3.51 (dd, *J* = 10.0, 4.4 Hz, 1H), 3.46 (dd, *J* = 12.8, 7.3 Hz, 1H), 3.26 (dd, *J* = 12.8, 5.6 Hz, 1H), 2.16 (s, 3H), 2.11 (s, 3H), 2.07 (s, 3H), 2.04 (s, 6H), 1.97 (s, 3H), 1.59–1.56 (b, 2H), 1.34–1.23 (m, 70H), 1.17 (s, 9H), 0.88 (t, *J* = 7.0 Hz, 6H, 2 × Me). ^13^C NMR (151 MHz, CDCl_3_): δ 177.1, 172.7, 170.4, 170.2, 170.1, 169.81, 169.8, 169.2, 137.2, 125.0, 100.71, 100.7, 75.4, 73.2, 72.9, 72.5, 72.4, 71.9, 71.1, 69.2, 67.8, 67.6, 62.1, 62.0, 50.6, 50.4, 38.9, 37.0, 32.5, 32.1, 29.9, 29.81, 29.8, 29.71, 29.7, 29.6, 29.5, 29.4, 29.2, 27.2, 25.9, 22.9, 21.1, 21.0, 20.9, 20.8, 20.7, 14.3. HR-ESI-MS *m*/*z*: [M + HCOO]^−^ Calcd for C_73_H_125_N_4_O_21_ 1393.8837; Found: 1393.8878.

### 3.17. (2S,3R,E)-2-Pentacosanamido-3-pivaloyloxy-pentacos-4-en-1-yl 2,3,4-tri-O-acetyl-6-azido-6-deoxy-β-D-galactopyranosyl-(1→4)-2,3,6-tri-O-acetyl-D-glucopyranoside (**14d**)

Compound **14d** (72%, 18.5 mg) was prepared as a white solid from **13d** (28 mg, 17.8 µmol) by the same method and conditions employed to prepare **14a**. TLC: R_f_ = 0.66 (EtOAc:Hex 40:60). ^1^H NMR (600 MHz, CDCl_3_): δ 5.75 (dt, *J* = 14.7, 7.4 Hz, 1H, =CH-), 5.61 (d, *J* = 9.2 Hz, 1H, -NHCO-), 5.36–5.32 (m, 2H, =CH- and 4′-H), 5.24–5.17 (m, 2H), 5.09 (dd, *J* = 10.4, 7.9 Hz, 1H), 4.96 (dd, *J* = 10.4, 3.5 Hz, 1H), 4.87 (dd, *J* = 9.5, 7.8 Hz, 1H), 4.50 (d, *J* = 7.9 Hz, 1H, anomeric), 4.48 (d, *J* = 1.7 Hz, 1H), 4.42 (d, *J* = 7.7 Hz, 1H, anomeric), 4.32 (ddd, *J* = 11.4, 8.2, 4.1 Hz, 1H), 4.05 (dd, *J* = 12.0, 4.9 Hz, 1H), 3.90 (dd, *J* = 9.9, 3.7 Hz, 1H), 3.85 (t, *J* = 9.5 Hz, 1H), 3.72 (t, *J* = 6.5 Hz, 1H), 3.60 (ddd, *J* = 9.8, 4.7, 2.0 Hz, 1H), 3.51 (dd, *J* = 10.0, 4.4 Hz, 1H), 3.47 (dd, *J* = 12.8, 7.3 Hz, 1H), 3.26 (dd, *J* = 12.8, 5.7 Hz, 1H), 2.17 (s, 3H), 2.12 (s, 3H), 2.07 (s, 3H), 2.04 (s, 6H, 2 × CH_3_), 1.97 (s, 3H), 1.59–1.56 (b, 2H), 1.32–1.23 (m, 84H), 1.17 (s, 9H), 0.88 (t, *J* = 7.0 Hz, 6H, 2 × Me). ^13^C NMR (151 MHz, CDCl_3_): δ 177.1, 172.7, 170.4, 170.2, 170.1, 169.81, 169.8, 169.2, 137.2, 125.0, 100.8, 100.7, 75.4, 73.2, 72.9, 72.5, 72.4, 72.0, 71.1, 69.2, 67.8, 67.6, 63.2, 62.0, 50.6, 50.4, 38.9, 37.0, 32.5, 32.1, 29.9, 29.89, 29.8, 29.71, 29.7, 29.6, 29.5, 29.4, 29.2, 27.2, 25.9, 22.9, 21.1, 20.91, 20.9, 20.8, 20.7, 14.3. HR-ESI-MS *m*/*z*: [M + CH_3_OH-H]^−^ Calcd for C_80_H_141_N_4_O_20_ 1478.0139; Found: 1478.0077.

### 3.18. (2S,3R,E)-2-Octadecanamido-3-hydroxy-octadec-4-en-1-yl 6-azido-6-deoxy-β-D-galactopyranosyl-(1→4)-β-D-glucopyranoside (**1a**)

To a solution of **14a** (19.5 mg, 15.6 µmol) in a mixture of dry DCM and MeOH (1:1, 2 mL) was added NaOMe in MeOH (5 M, 43.6 µL, 0.2 mmol) dropwise at 0 °C. The ice bath was removed, and the mixture was stirred at rt under N_2_ for 2 d. After the starting material was completely consumed as indicated by TLC, Amberlyst H^+^ resin was added with stirring to neutralize the reaction mixture. The solvent was removed under vacuum, and the residue was subjected to silica gel column chromatography to afford **1a** as an off-white solid (90%, 12.8 mg). TLC: R_f_ = 0.51 (DCM:MeOH 85:15). ^1^H NMR (600 MHz, CDCl_3_:CD_3_OD 2:1): δ 5.65 (dt, *J* = 15.0, 7.4 Hz, 1H, =CH-), 5.41 (dd, *J* = 15.0, 7.6 Hz, 1H, =CH-), 4.32 (d, *J* = 7.8 Hz, 1H anomeric), 4.25 (d, *J* = 7.8 Hz, 1H anomeric), 4.17 (dd, *J* = 10.0, 3.9 Hz, 1H), 4.05 (t, *J* = 7.6 Hz, 1H), 3.96–3.93 (m, 1H), 3.82 (d, *J* = 3.0 Hz, 2H), 3.77 (d, *J* = 2.9 Hz, 1H), 3.65–3.61 (m, 2H), 3.60–3.58 (m, 1H), 3.56–3.52 (m, 4H), 3.50 (dd, *J* = 9.8, 3.0 Hz, 2H), 3.47 (dd, *J* = 9.7, 3.2 Hz, 1H), 3.38–3.35 (m, 1H), 2.13 (t, *J* = 7.7 Hz, 2H), 2.00–1.96 (m, 2H), 1.56–1.52 (m, 2H), 1.34–1.30 (m, 2H), 1.28–1.20 (m, 48H), 0.84 (t, *J* = 7.0 Hz, 6H, 2 × Me). ^13^C NMR (151 MHz, CDCl_3_:CD_3_OD 2:1): δ 175.0, 134.8, 129.7, 104.3, 103.3, 80.4, 75.3, 75.2, 74.0, 73.8, 73.7, 72.4, 71.3, 69.2, 69.0, 61.2, 53.6, 51.6, 36.9, 32.8, 32.3, 30.12, 30.11, 30.1, 30.03, 30.02, 30.01, 30.0, 29.9, 29.8, 29.72, 29.71, 29.7, 26.4, 23.0, 14.3. HR-ESI-MS *m*/*z*: [M + HCOO]^−^ Calcd for C_49_H_91_N_4_O_14_ 959.6532; Found: 959.6563.

### 3.19. (2S,3R,E)-2-Pentacosanamido-3-(hydroxy)-octadec-4-en-1-yl 6-azido-6-deoxy-β-D-galactopyranosyl-(1→4)-β-D-glucopyranoside (**1b**)

Compound **1b** (88%, 11.3 mg) was prepared as a white solid from **14b** (17 mg, 12.6 µmol) by the same method and conditions utilized to prepare **1a**. TLC: R_f_ = 0.66 (DCM:MeOH 85:15). ^1^H NMR (600 MHz, CDCl_3_:CD_3_OD 2:1): δ 5.63 (dt, *J* = 14.9, 7.2 Hz, 1H, =CH-), 5.40 (dd, *J* = 14.9, 7.5 Hz, 1H, =CH-), 4.30 (d, *J* = 7.8 Hz, 1H anomeric), 4.23 (d, *J* = 7.8 Hz, 1H anomeric), 4.14 (dd, *J* = 10.1, 3.9 Hz, 1H), 4.04 (t, *J* = 7.4 Hz, 1H), 3.80 (d, *J* = 3.0 Hz, 2H), 3.76 (d, *J* = 3.0 Hz, 1H), 3.61–3.59 (m, 2H), 3.58–3.56 (m, 4H), 3.54–3.51 (m, 4H), 3.49 (dd, *J* = 10.4, 1.9 Hz, 1H), 3.46 (dd, *J* = 9.7, 3.2 Hz, 1H), 2.11 (t, *J* = 7.7 Hz, 2H), 1.98–1.94 (m, 2H), 1.54–1.50 (m, 2H), 1.31–1.28 (m, 2H), 1.26–1.19 (m, 62H), 0.83 (t, *J* = 7.0 Hz, 6H, 2 × Me). ^13^C NMR (151 MHz, CDCl_3_:CD_3_OD 2:1): δ 174.6, 134.6, 129.2, 104.0, 103.0, 80.2, 75.0, 74.9, 73.7, 73.4, 72.3, 71.0, 68.8, 68.7, 63.6, 61.0, 53.5, 53.3, 51.3, 36.6, 32.5, 32.0, 29.82, 29.81, 29.8, 29.71, 29.7, 29.6, 29.52, 29.51, 29.5, 29.4, 26.0, 22.8, 14.1. HR-ESI-MS *m*/*z*: [M + HCOO]^−^ Calcd for C_56_H_105_N_4_O_14_ 1057.7628; Found: 1057.7659.

### 3.20. (2S,3R,E)-2-Octadecanamido-3-(hydroxy)-pentacos-4-en-1-yl 6-azido-6-deoxy-β-D-galactopyranosyl-(1→4)-β-D-glucopyranoside (**1c**)

Compound **1c** (80%, 10.8 mg) was prepared as an off-white solid from **14c** (18 mg, 13.3 µmol) by the same method and conditions utilized to prepare **1a**. TLC: R_f_ = 0.70 (DCM:MeOH 85:15). ^1^H NMR (600 MHz, CDCl_3_:CD_3_OD 2:1): δ 5.64 (dt, *J* = 14.9, 7.3 Hz, 1H, =CH-), 5.41 (dd, *J* = 14.9, 7.6 Hz, 1H, =CH-), 4.30 (1H, anomeric, overlapped with DHO signal), 4.25 (d, *J* = 7.8 Hz, 1H, anomeric), 4.16 (dd, *J* = 10.0, 3.9 Hz, 1H), 4.05 (t, *J* = 7.7 Hz, 1H), 3.96–3.92 (m, 2H), 3.92–3.89 (m, 3H), 3.82 (d, *J* = 3.3 Hz, 2H), 3.77 (d, *J* = 3.3 Hz, 1H), 3.63–3.58 (m, 2H), 3.55–3.52 (m, 3H), 3.50 (dd, *J* = 10.5, 3.3 Hz, 1H), 3.47 (dd, *J* = 9.7, 3.2 Hz, 1H), 2.15–2.10 (m, 2H), 1.99–1.96 (m, 2H), 1.56–1.52 (m, 2H), 1.34–1.29 (m, 2H), 1.27–1.20 (m, 62H), 0.84 (t, *J* = 7.0 Hz, 6H, 2 × Me). ^13^C NMR (151 MHz, CDCl_3_:CD_3_OD 2:1): δ 174.9, 134.8, 129.6, 104.2, 103.2, 80.4, 75.2, 75.1, 74.0, 73.7, 73.6, 72.4, 71.2, 70.6, 69.1, 61.1, 55.3, 53.5, 51.5, 49.9, 49.6, 36.8, 32.7, 32.2, 30.1, 30.03, 30.02, 30.01, 30.0, 29.91, 29.9, 29.8, 29.73, 29.72, 29.71, 29.7, 29.6, 26.3, 23.0, 14.2. HR-ESI-MS *m*/*z*: [M + HCOO]^−^ Calcd for C_56_H_105_N_4_O_14_ 1057.7628; Found: 1057.7659.

### 3.21. (2S,3R,E)-2-Pentacosanamido-3-(hydroxy)-pentacos-4-en-1-yl 6-azido-6-deoxy-β-D-galactopyranosyl-(1→4)-β-D-glucopyranoside (**1d**)

Compound **1d** (85%, 11.1 mg) was prepared as a white solid from **14d** (17 mg, 11.7 µmol) by the same method and conditions used to prepare **1a**. TLC: R_f_ = 0.75 (DCM:MeOH 85:15). ^1^H NMR (600 MHz, CDCl_3_:CD_3_OD 2:1): δ 5.63 (dt, *J* = 14.9, 7.3 Hz, 1H, =CH-), 5.39 (dd, *J* = 14.9, 7.5 Hz, 1H, =CH-), 4.30 (d, *J* = 7.8 Hz, 1H, anomeric), 4.23 (d, *J* = 7.8 Hz, 1H, anomeric), 4.13 (dd, *J* = 10.0, 4.0 Hz, 1H), 4.04 (t, *J* = 7.4 Hz, 1H), 3.94–3.90 (m, 1H), 3.79 (d, *J* = 2.7 Hz, 3H), 3.61–3.56 (m, 3H), 3.54–3.52 (m, 2H), 3.51 (dd, *J* = 7.8, 3.1 Hz, 2H), 3.48 (dd, *J* = 10.7, 3.2 Hz, 1H), 3.44–3.44 (m, 3H), 2.11 (t, *J* = 7.6 Hz, 2H), 1.94–1.97 (m, 2H), 1.59–1.47 (m, 4H), 1.31–1.16 (m, 76H), 0.82 (t, *J* = 7.0 Hz, 6H). ^13^C NMR (151 MHz, CDCl_3_:CD_3_OD 2:1): δ 177.6, 134.5, 129.0, 103.8, 102.8, 79.9, 74.8, 73.6, 73.21, 73.2, 72.2, 70.8, 68.61, 68.6, 63.5, 60.9, 53.1, 51.1, 36.5, 32.3, 31.9, 29.71, 29.7, 29.61, 29.6, 29.51, 29.5, 29.42, 29.41, 29.4, 29.31, 29.3, 29.21, 29.2, 25.9, 22.6, 14.0. HR-ESI-MS *m*/*z*: [M + HCOO]^−^ Calcd for C_63_H_119_N_4_O_14_ 1155.8723; Found: 1155.87622.1.

## 4. Conclusions

β-LacCer is not only a key intermediate in complex GSL biosynthesis but also an important player in many biological events and diseases. However, in-depth investigation of β-LacCer is hindered by its lack of fluorophores or other useful functionalities. To overcome this problem, we designed and synthesized a series of β-LacCer analogs carrying an azido group in its glycan and varied Cer moieties. In the literature, there are many reports [33,34,35,36,37,38,39,40,41,42,43,44,45,46] (just cite a few) about the synthesis of β-LacCer and its analogs, but none of these reports have described azide-functionalized β-LacCer analogs like **1a**–**d** in this work. To facilitate rapid access to **1a**–**d**, we have employed a diversity-oriented synthetic strategy that is highlighted by the late-stage lipid remodeling for on-site assembly of the Cer moiety. Therefore, all the target molecules **1a**–**d** could be readily derived from the same intermediate **10** within five robust steps. This strategy and its protocols are streamlined so that they will be applicable to the synthesis of various lipid forms of β-LacCer and its analogs. The azide in **1a**–**d** is suitable for further and direct functionalization by means of straightforward and biocompatible click chemistry to attach a diversity of molecular tags, such as affinity and fluorescent tags that enable different modern analytical technologies. Alternatively, the azido group in **1a**–**d** can be reduced to generate a free primary amino group that is suitable for further introduction of molecular labels via chemoselective *N*-acylation. Moreover, additional functionalization of **1a**–**d** through biocompatible click chemistry can be conducted either before or after their introduction to cells/tissues [24,25], thereby expanding their application scopes. The flexibility to introduce fluorophores or other large molecular labels later also makes **1a**–**d** especially valuable. Because having a hydroxyl group substituted with a small azido group will have a minimal impact on the interaction of these probes with target molecules in/on cells and probably make the probes more easily acceptable by enzymes, these probes can better mimic the behaviors and bioactivities of β-LacCer than those containing large functional groups. Consequently, this work not only has established a novel and efficient method for the synthesis of β-LacCer and its analogs but also has provided **1a**–**d** as powerful probes or tools for in-depth investigation of β-LacCer biology, including the influences of its lipid structures on the biological functions and metabolisms of β-LacCer and other GSLs, as well as their related diseases.

## Data Availability

The original contributions presented in this study are included in the article/Supplementary Material. Further inquiries can be directed to the corresponding author.

## Supplementary Materials

The following supporting information can be downloaded at: https://www.mdpi.com/article/10.3390/molecules30132667/s1, the NMR and MS spectra of all new compounds, including **4**, **5**, **6**, **7**, **8** (only NMR), **10**, **12a,b**, **13a**–**d**, **14a**–**d**, and **1a**–**d**.
