# Supplementary material for: Synthesis of Azide-Labeled β-Lactosylceramide Analogs Containing Different Lipid Chains as Useful Glycosphingolipid Probes
Source: Molecules. 2025 Jun 20;30(13):2667. doi: 10.3390/molecules30132667 (PMC12251015; doi:10.3390/molecules30132667)

Supporting Information

Table of Contents

NMR and MS spectra of compound **4**..SI-2 to 4
NMR and MS spectra of compound **5**..SI-5 to 7
NMR and MS spectra of compound **6**..SI-8 to 10
NMR and MS spectra of compound **7**..SI-11 to 13
NMR spectra of compound **8**...SI-14 to 15
NMR and MS spectra of compound **10** ....SI-16 to 20
NMR and MS spectra of compounds**12a,b**..SI-21 to 30
NMR and MS spectra of compounds **13a-d**..SI-31 to 50
NMR and MS spectra of compounds **14a-d**..SI-51 to 70
NMR and MS spectra of compounds **1a-d**..SI-71 to 90

NMR and MS spectra for all new compounds

Figure S1. ^{1}H NMR of compound **4** (500 MHz, $CDCl_3$)

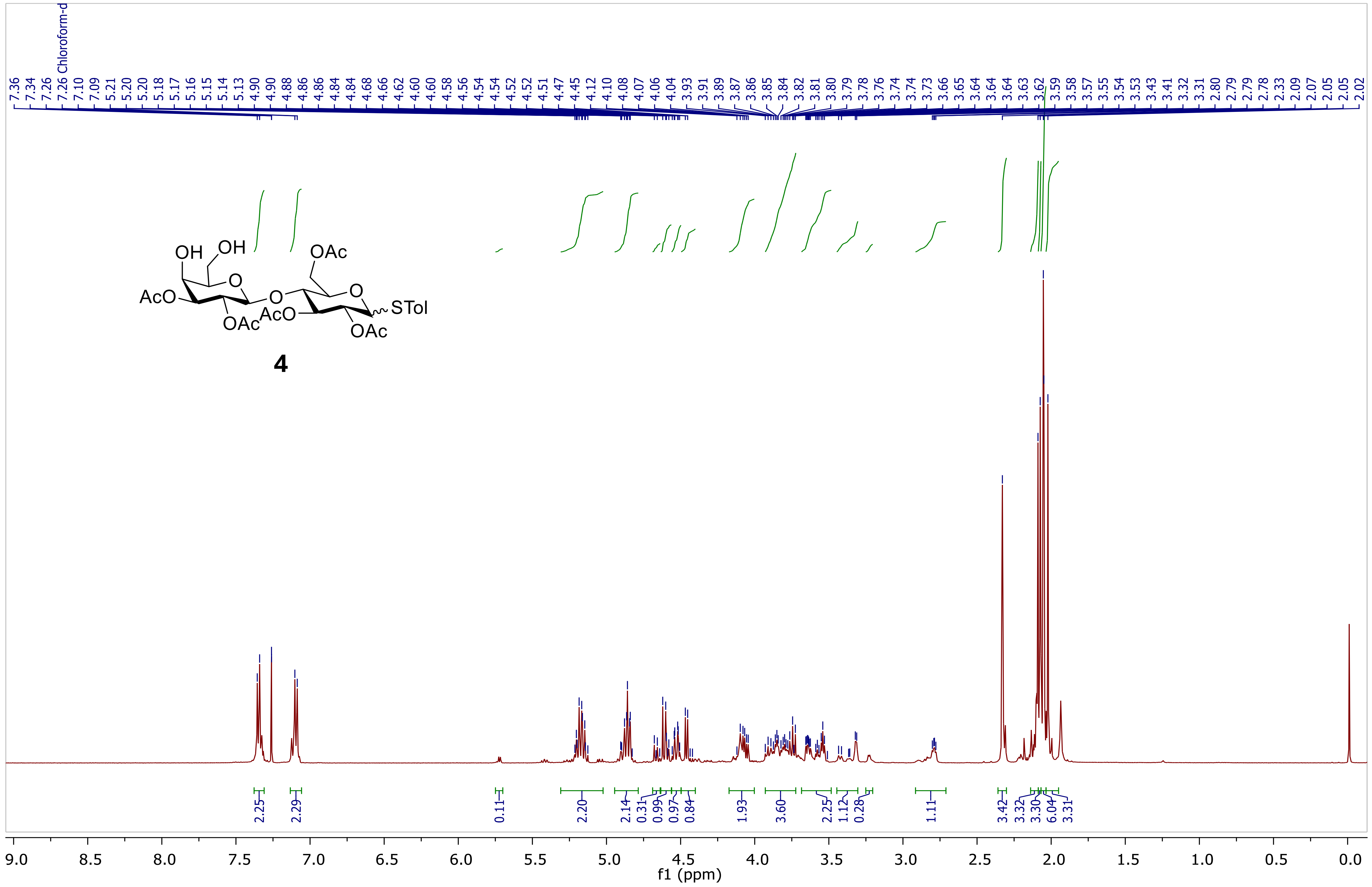

Figure S2. ^{13}C NMR of compound **4** (126 MHz, $CDCl_3$)

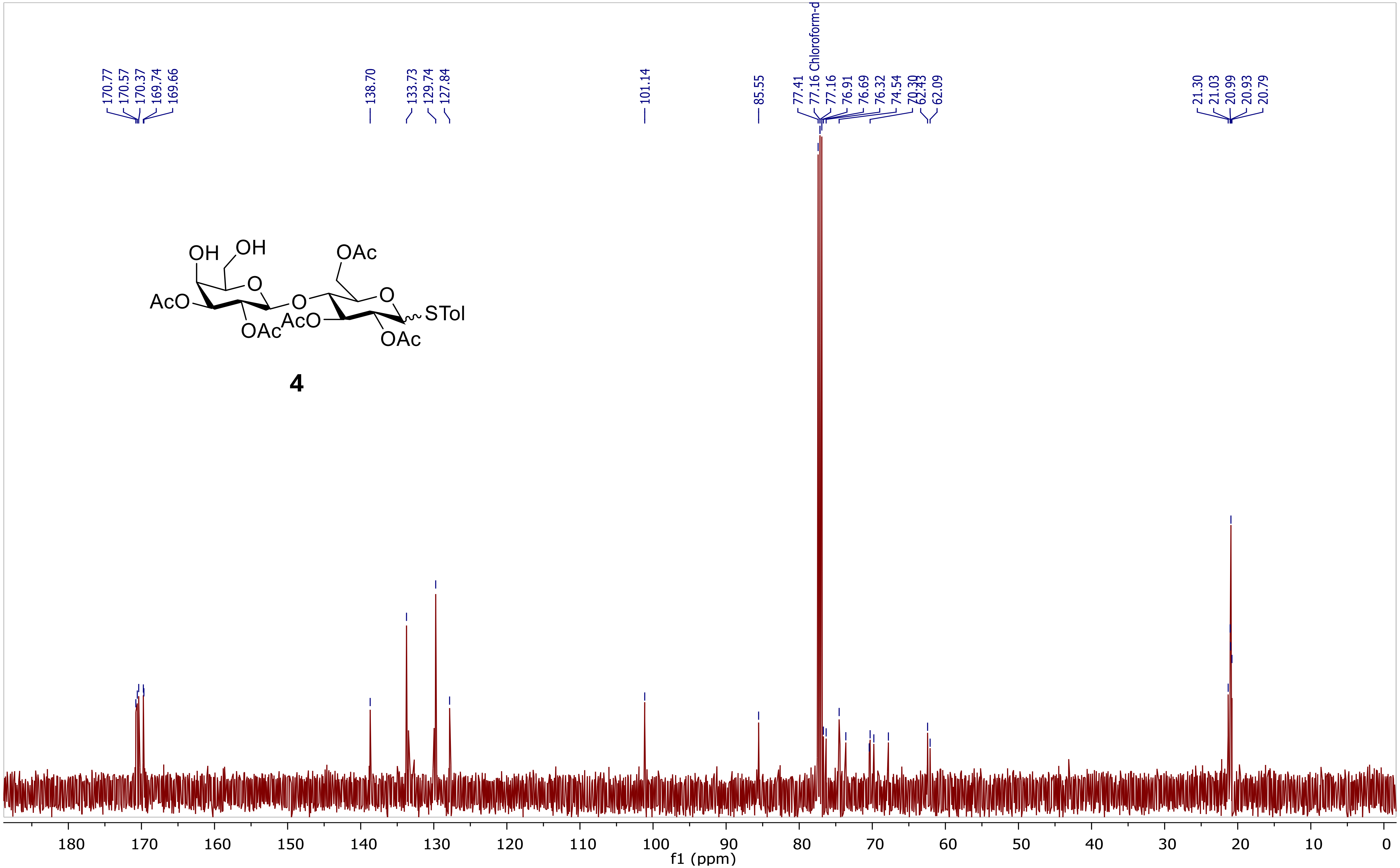

Figure S3. HR ESI-TOF-MS of compound **4**

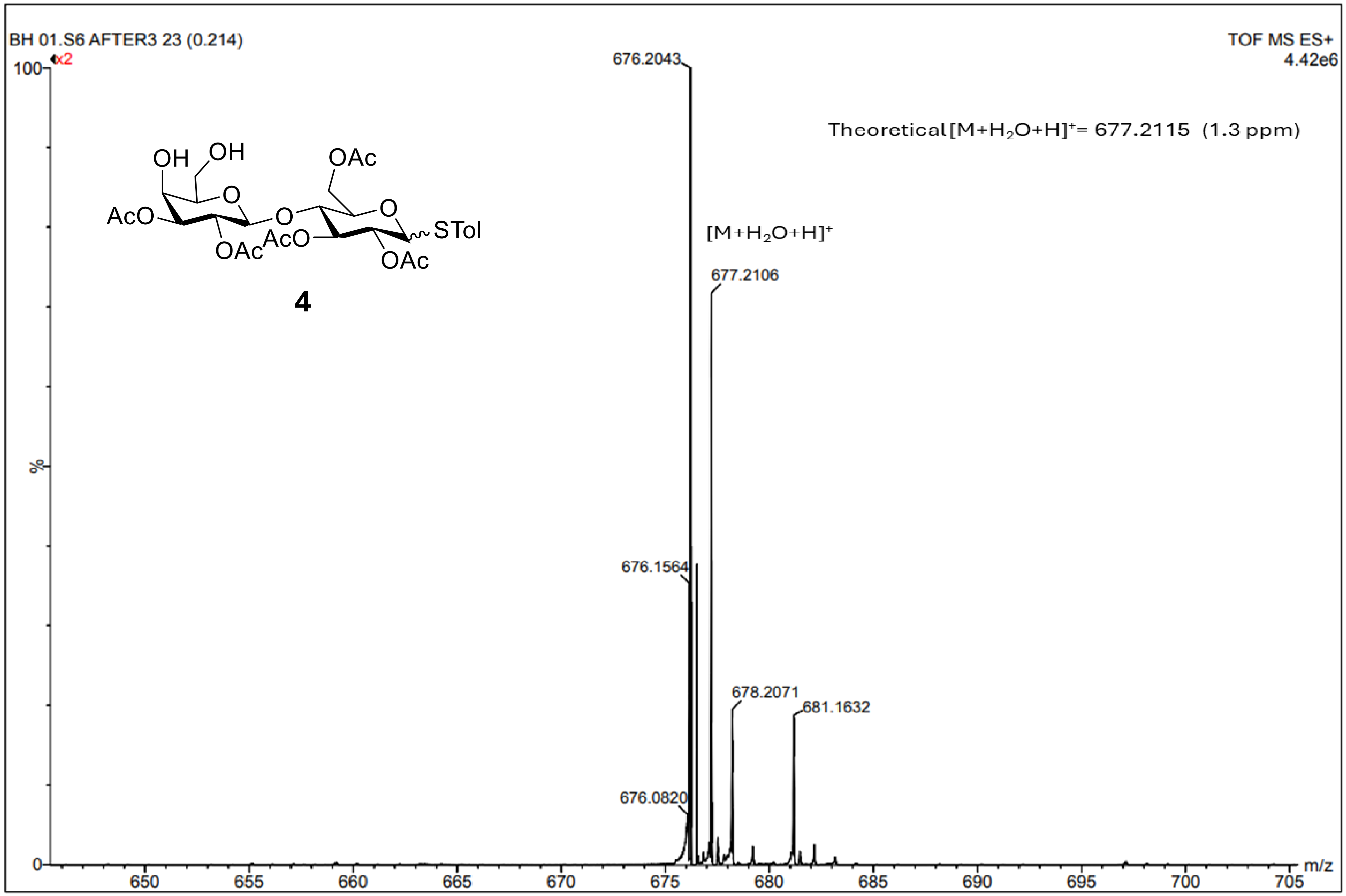

Figure S4. ^{1}H NMR of compound **5** (400 MHz, $CDCl_3$)

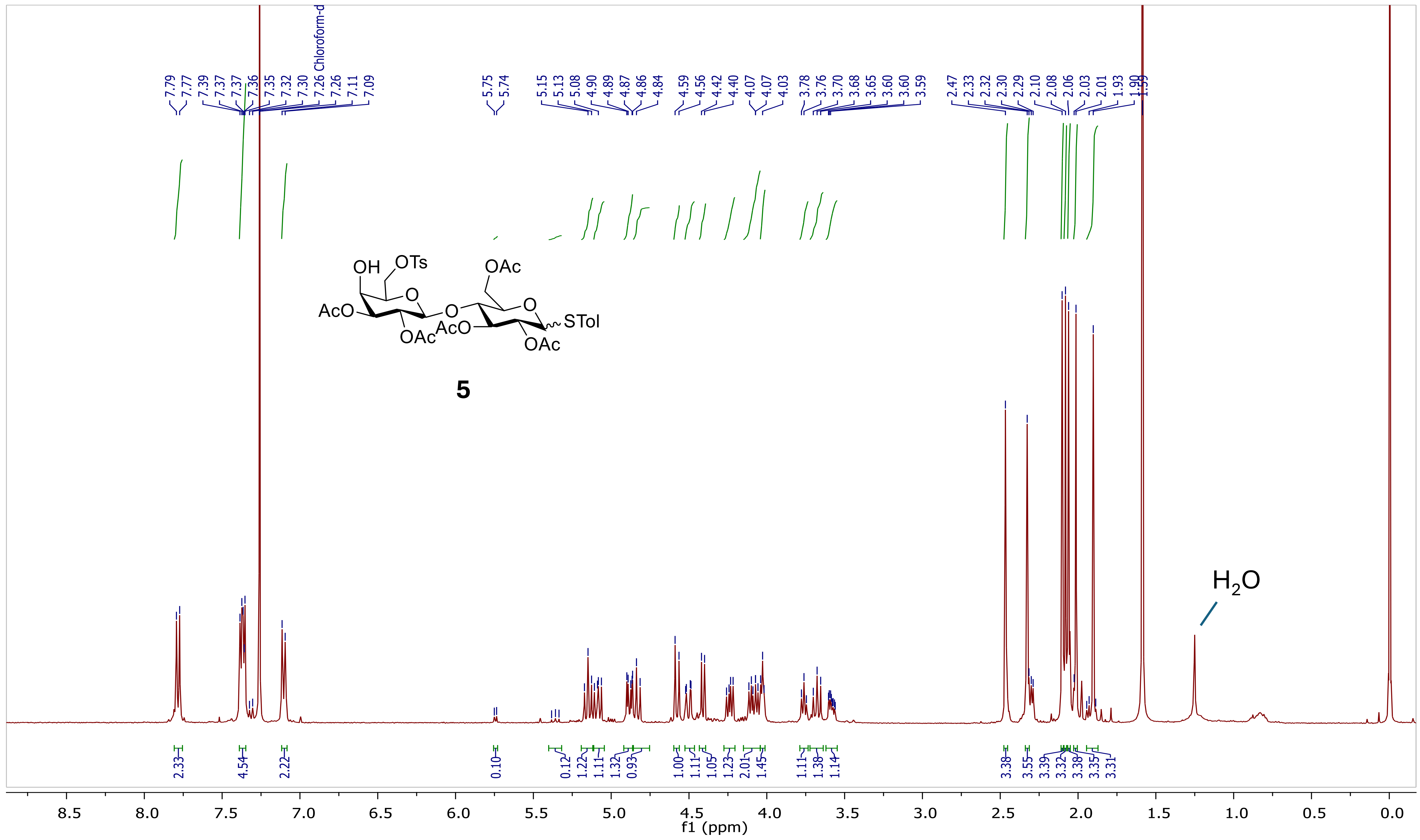

Figure S5. ^{13}C NMR of compound **5** (101 MHz, $CDCl_3$)

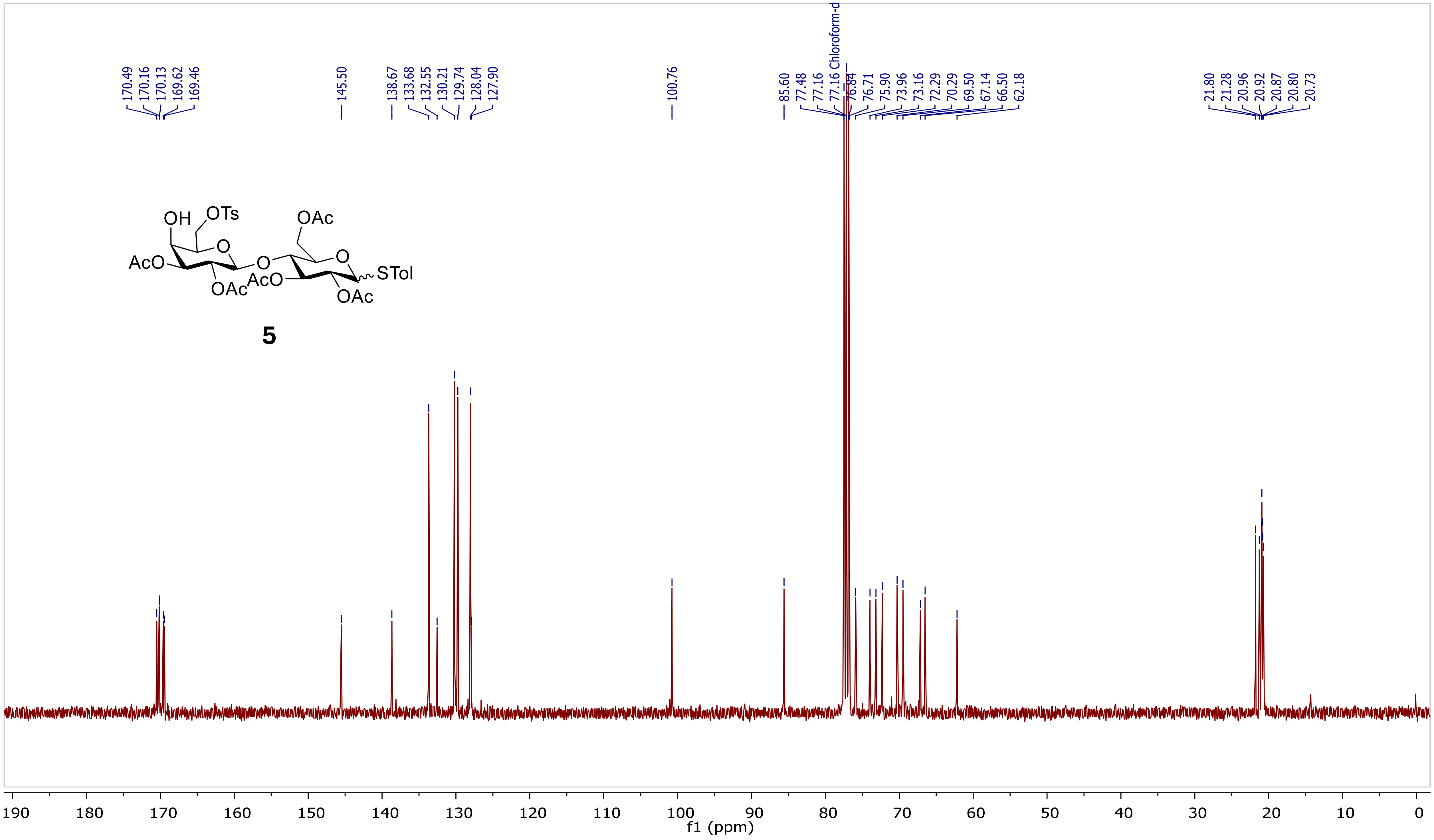

Figure S6. HR ESI-TOF-MS of compound **5**

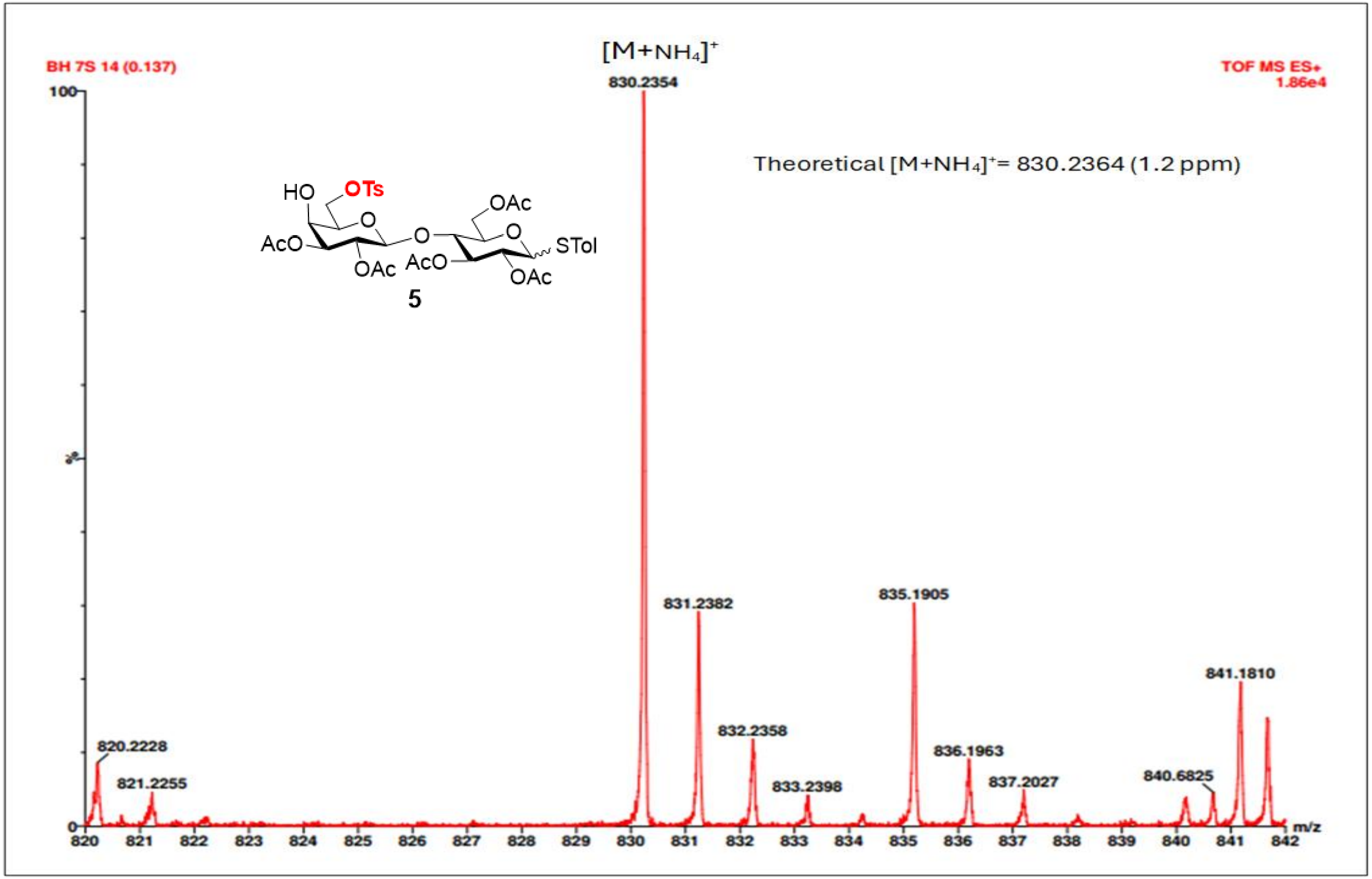

Figure S7. ^{1}H NMR of compound **6** (400 MHz, $CDCl_3$)

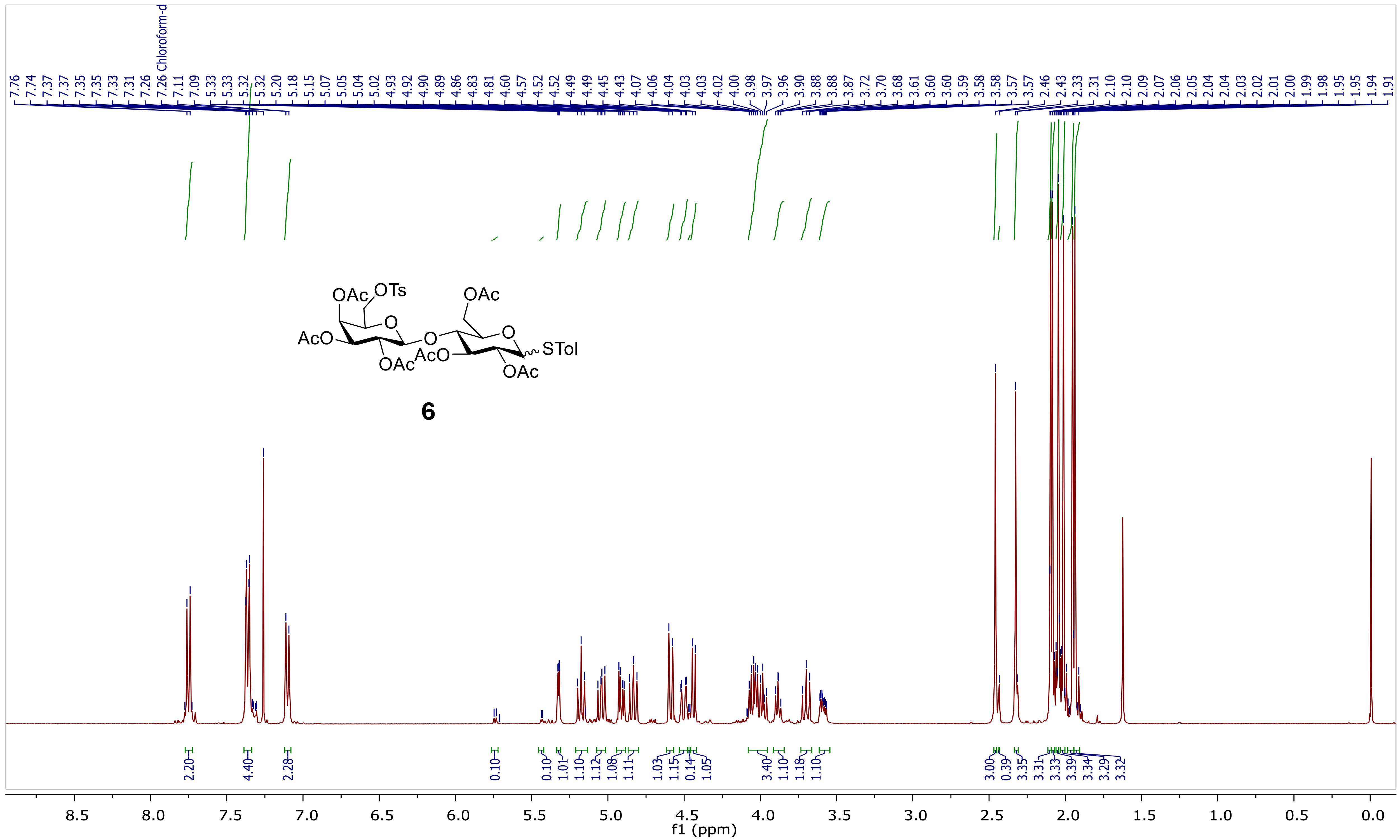

Figure S8. ^{13}C NMR of Compound **6** (101 MHz, $CDCl_3$)

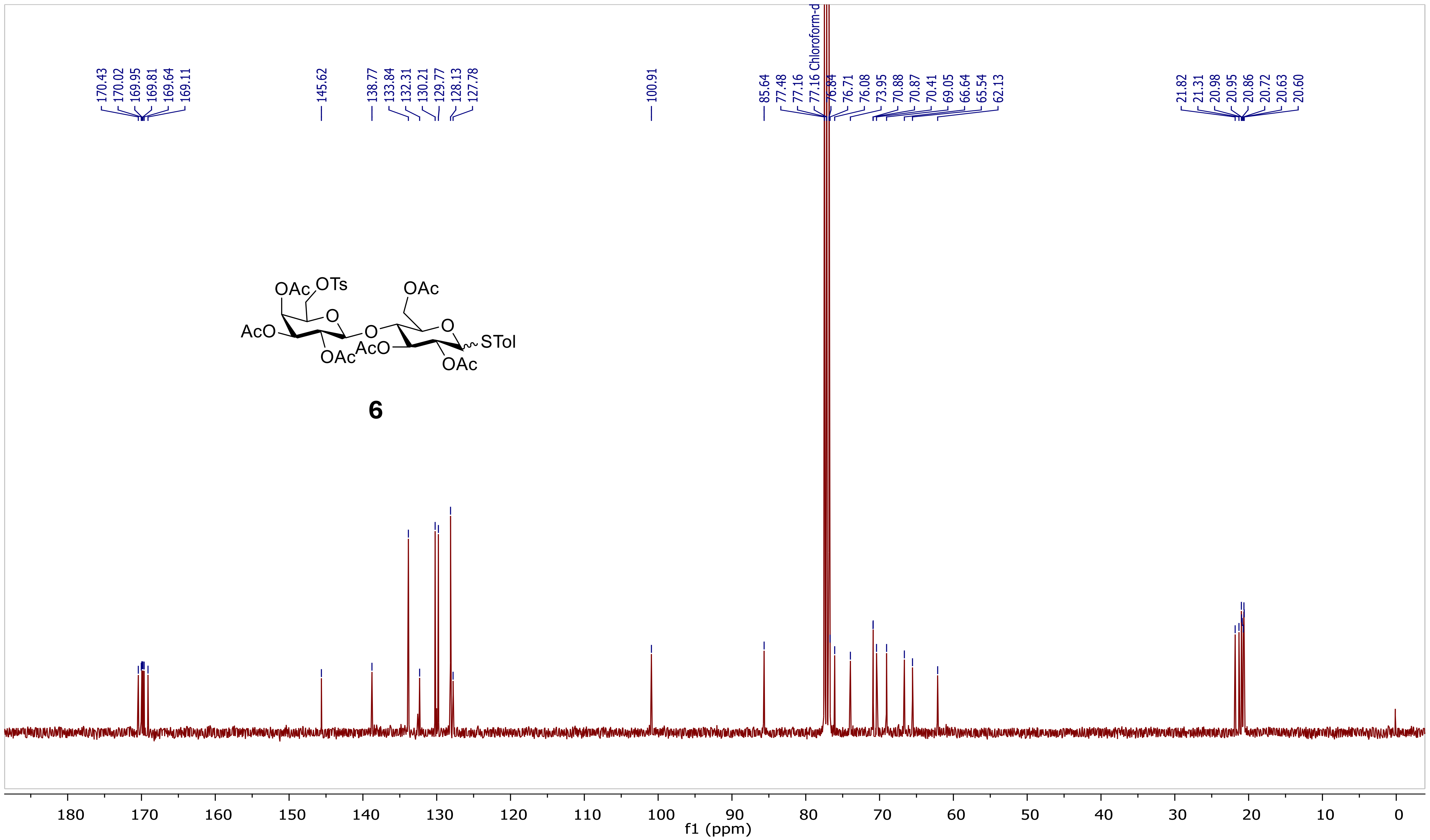

Figure S9. HR ESI-TOF-MS of compound **6**

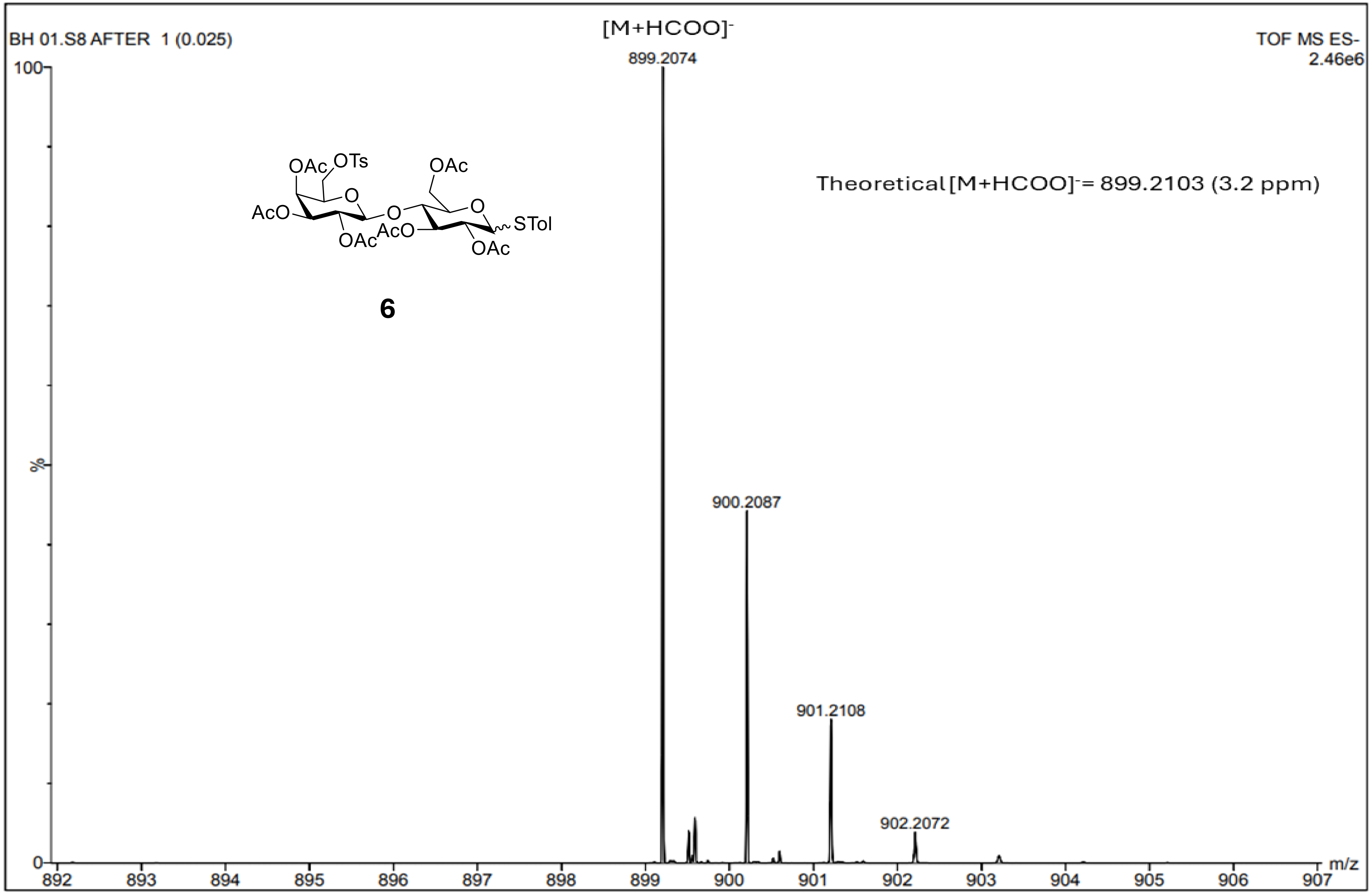

Figure S10. ^{1}H NMR of compound **7** (400 MHz, $CDCl_3$)

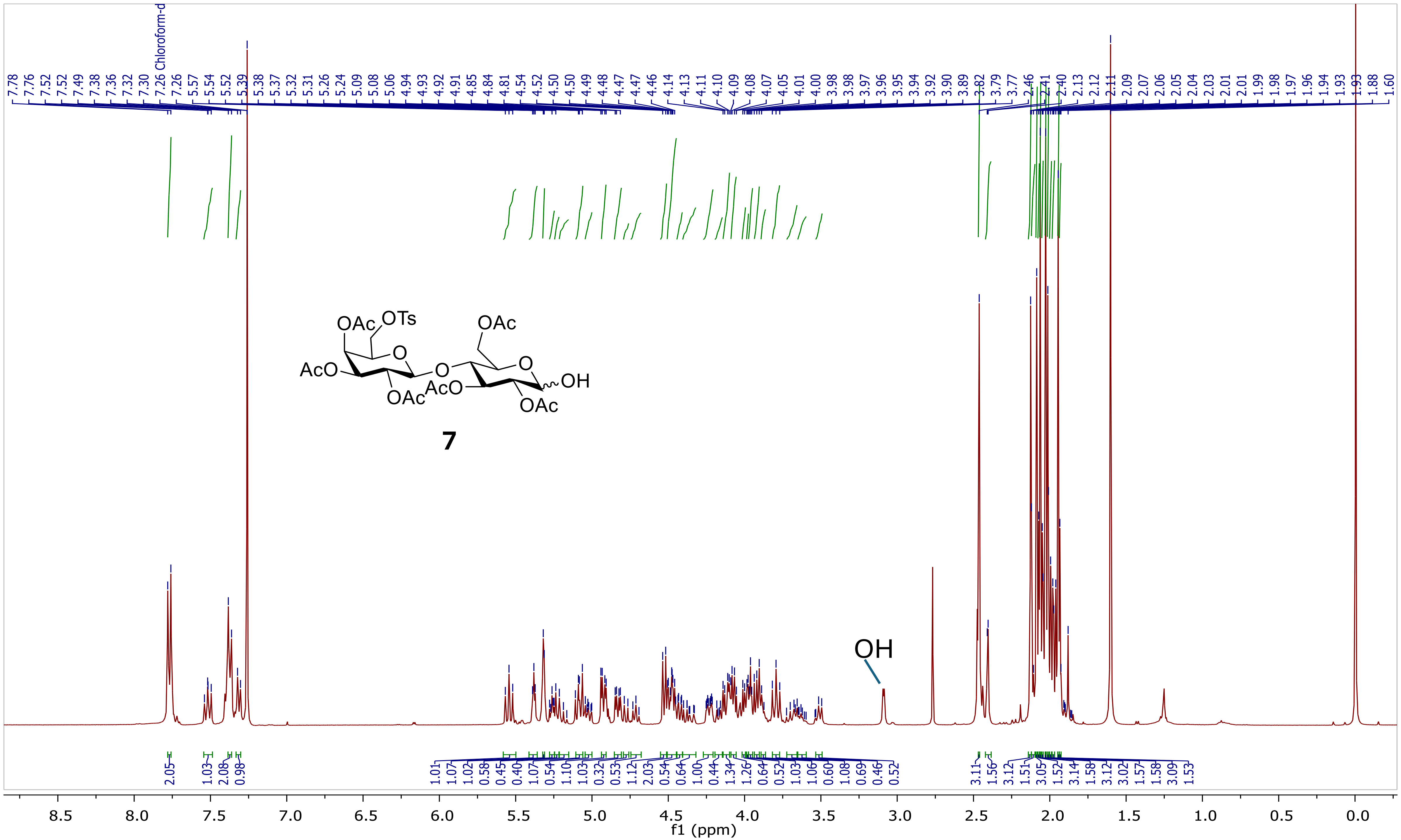

Figure S11. ^{13}C NMR of compound **7** (101 MHz, $CDCl_3$)

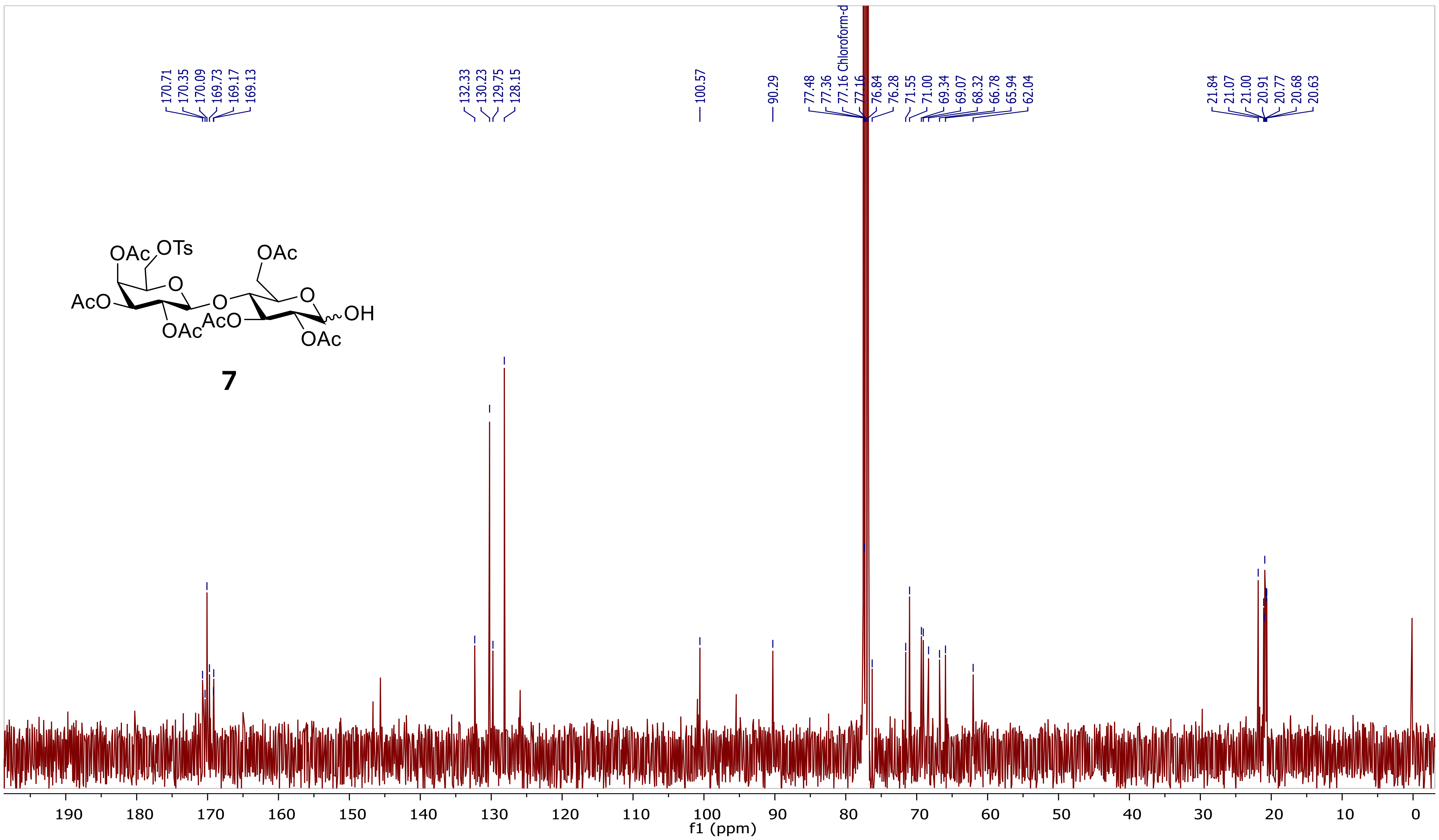

Figure S12. HR ESI-TOF-MS of compound **7**

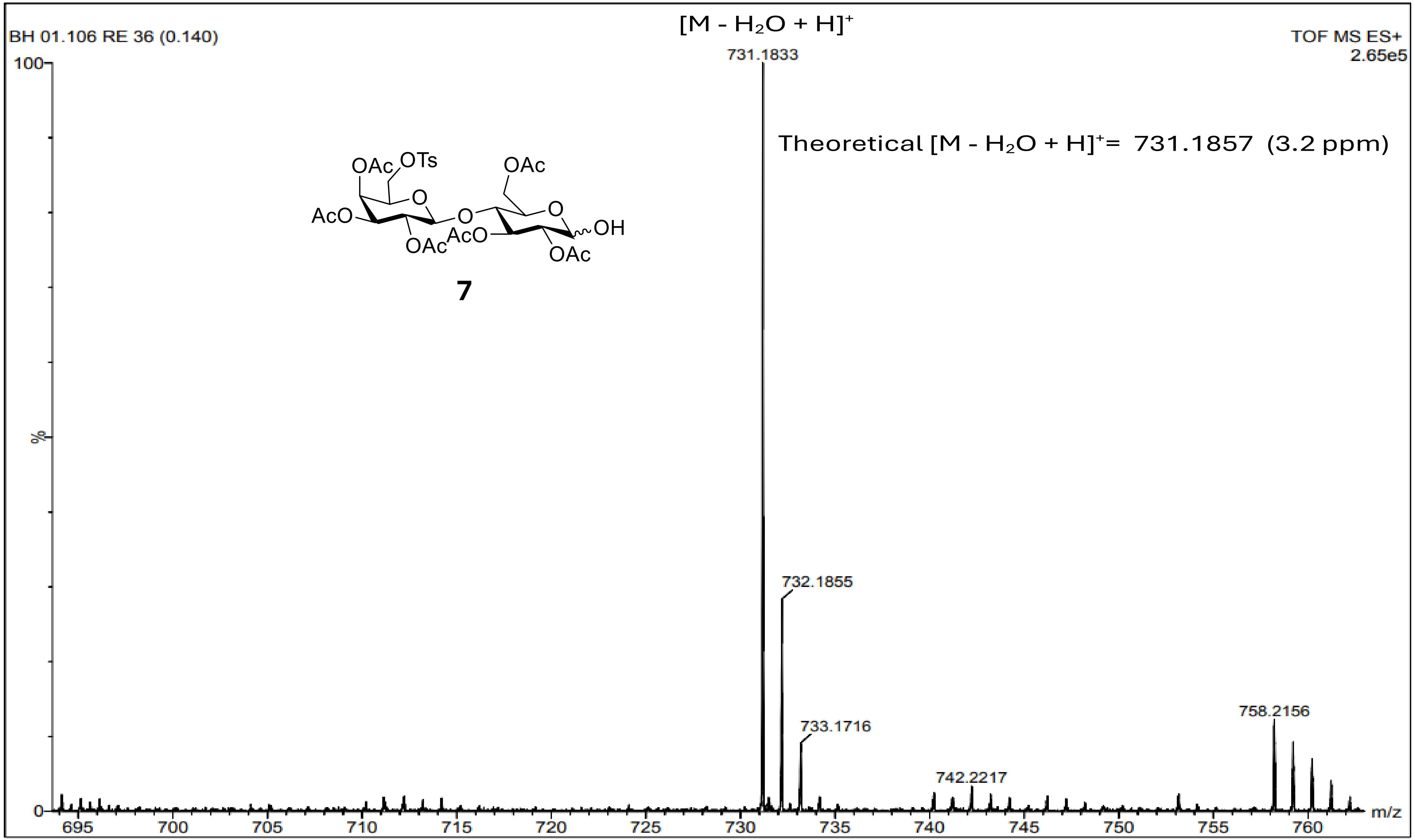

Figure S13. ^{1}H NMR of compound **8** (400 MHz, $CDCl_3$)

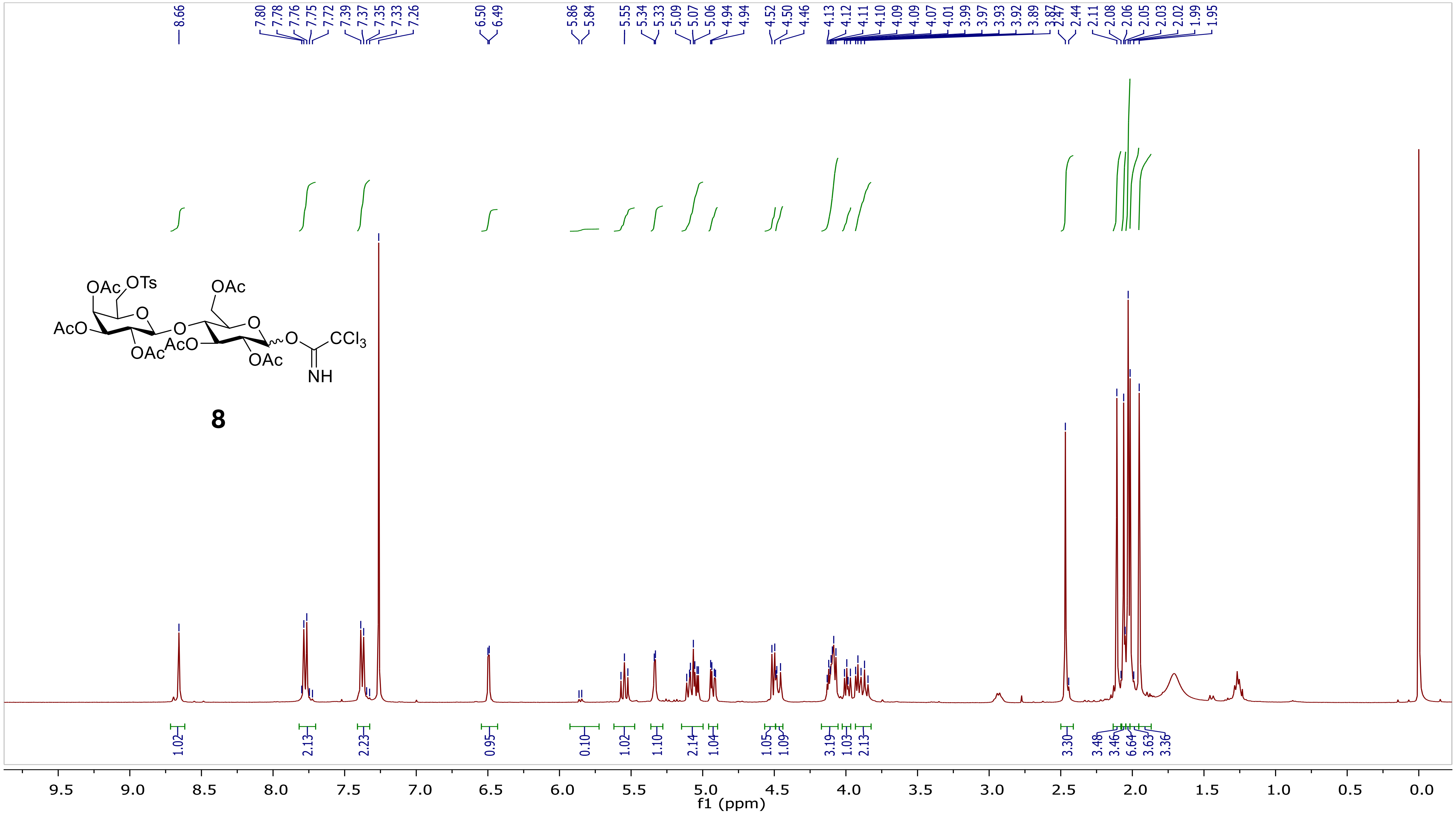

Figure S14. ^{13}C NMR of compound **8** (101 MHz, $CDCl_3$)

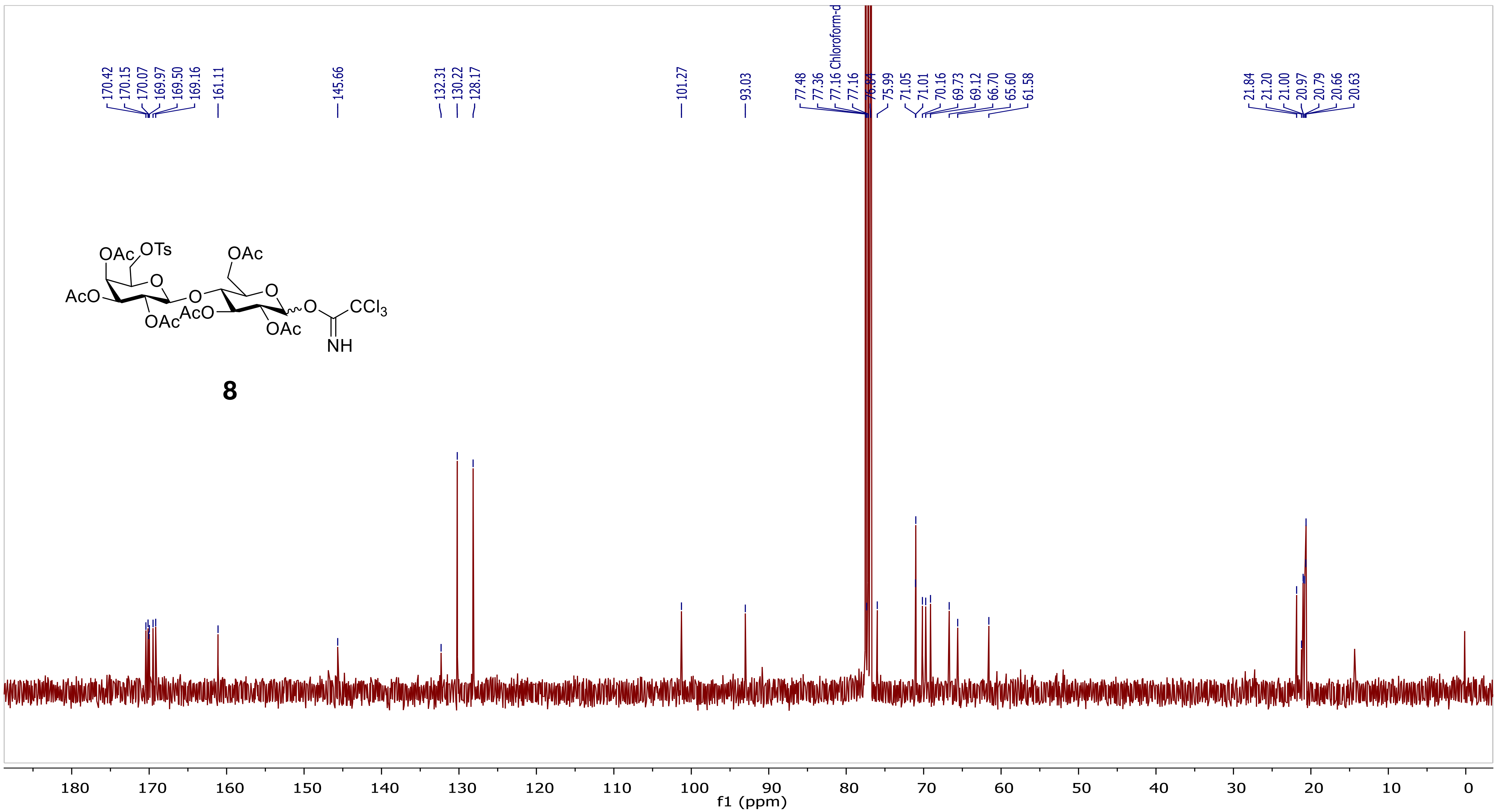

Figure S15. ^{1}H NMR of compound **10** (600 MHz, $CDCl_3$)

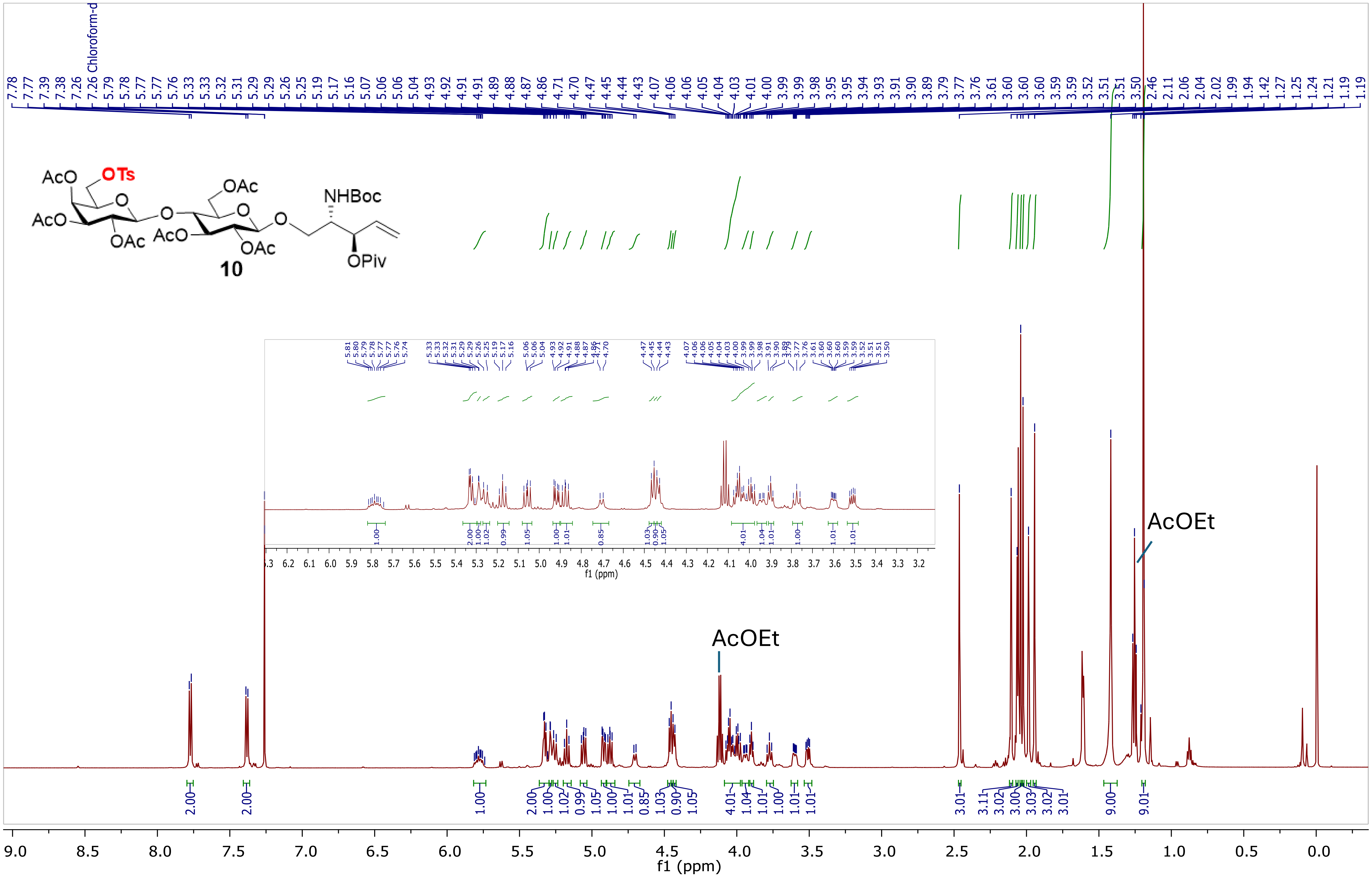

Figure S16. ^{13}C NMR of compound **10** (151 MHz, $CDCl_3$)

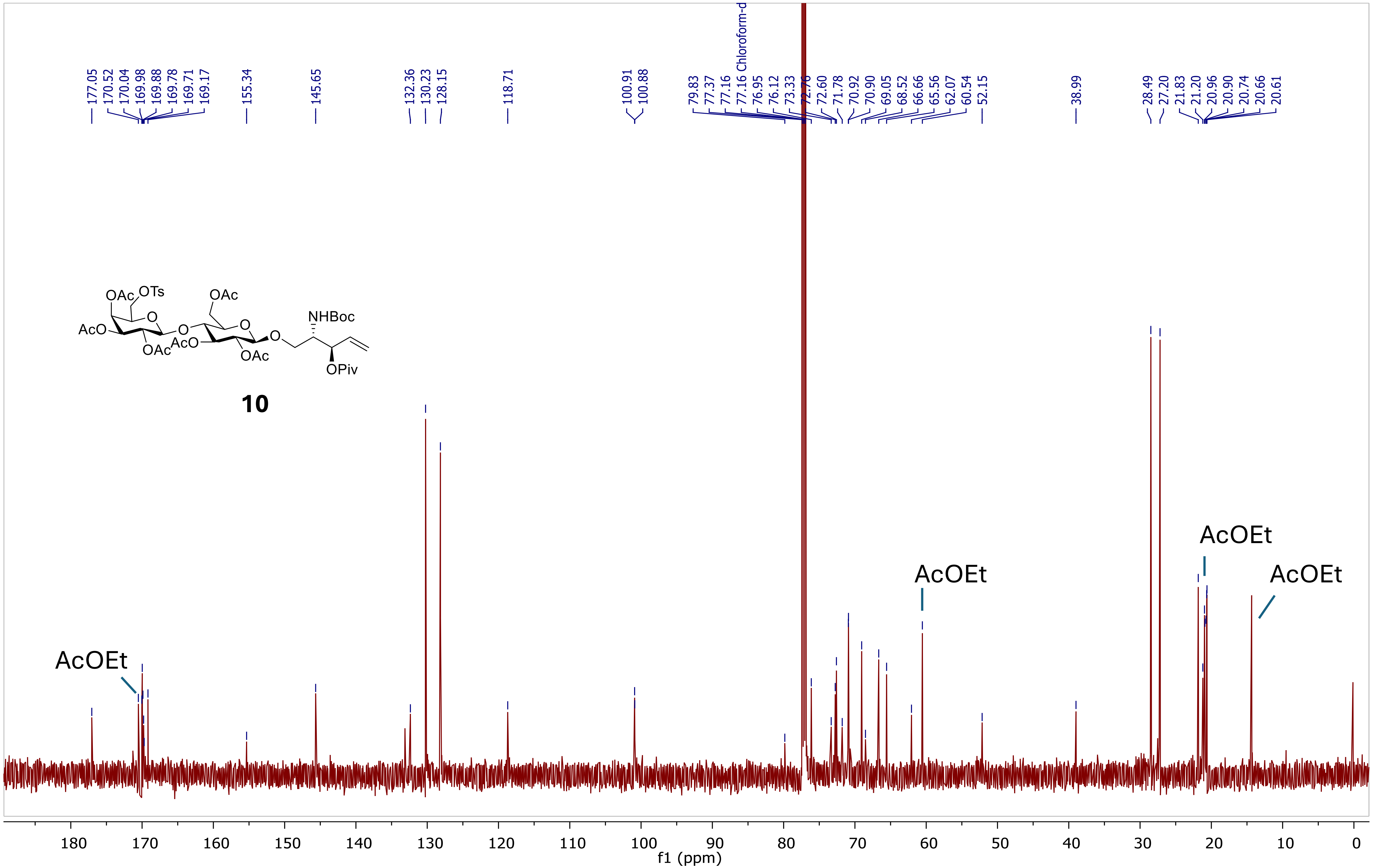

Figure S17. ^{1}H-^{1}H COSY NMR (600 MHz, $CDCl_3$) of compound **10**

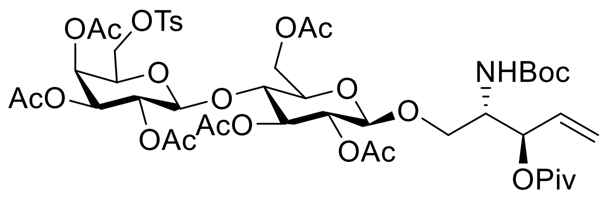

10

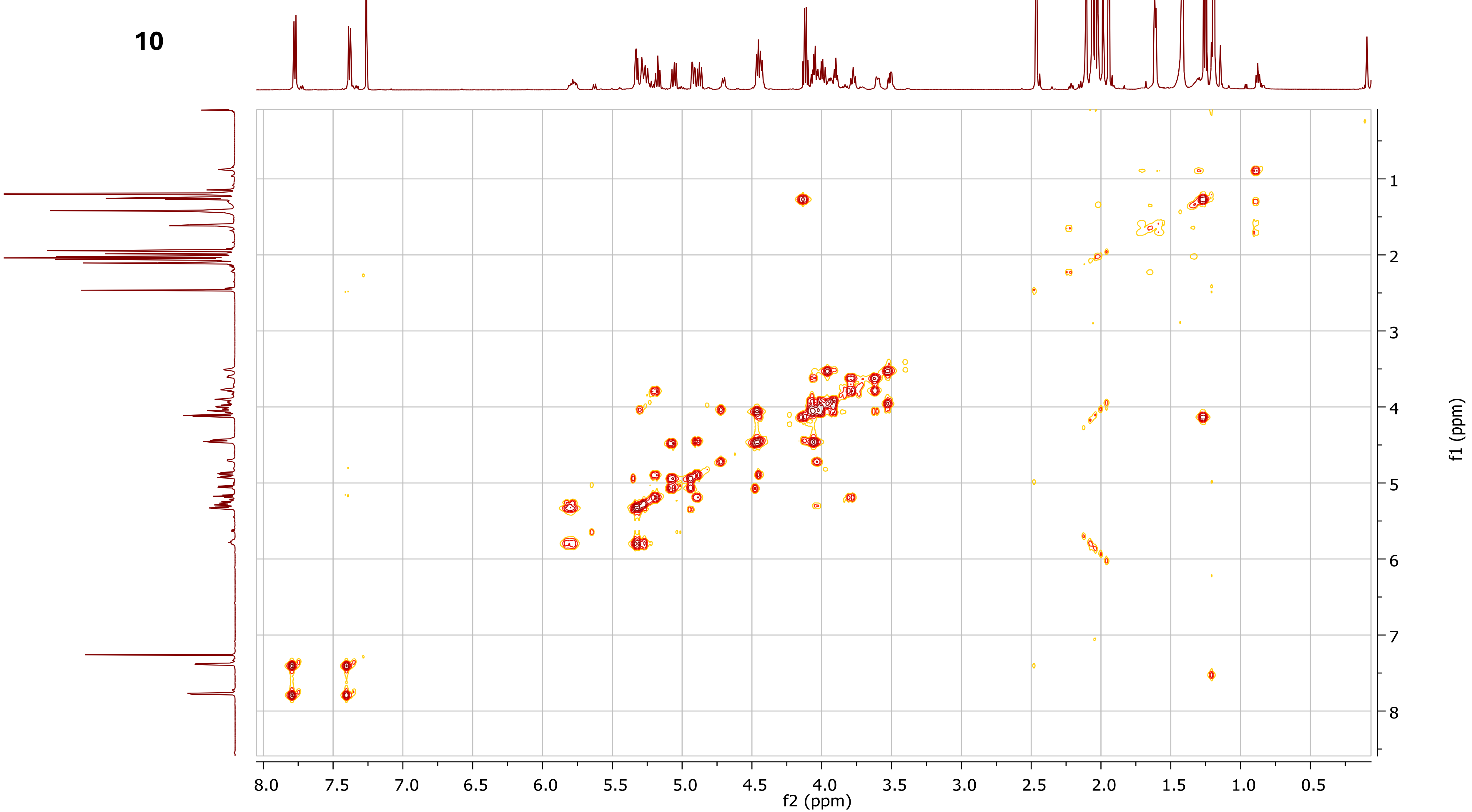

Figure S18. ^{1}H-^{13}C HSQC NMR (600/151 MHz, $CDCl_3$) of compound **10**

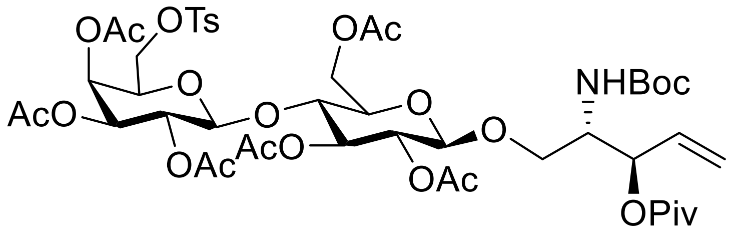

10

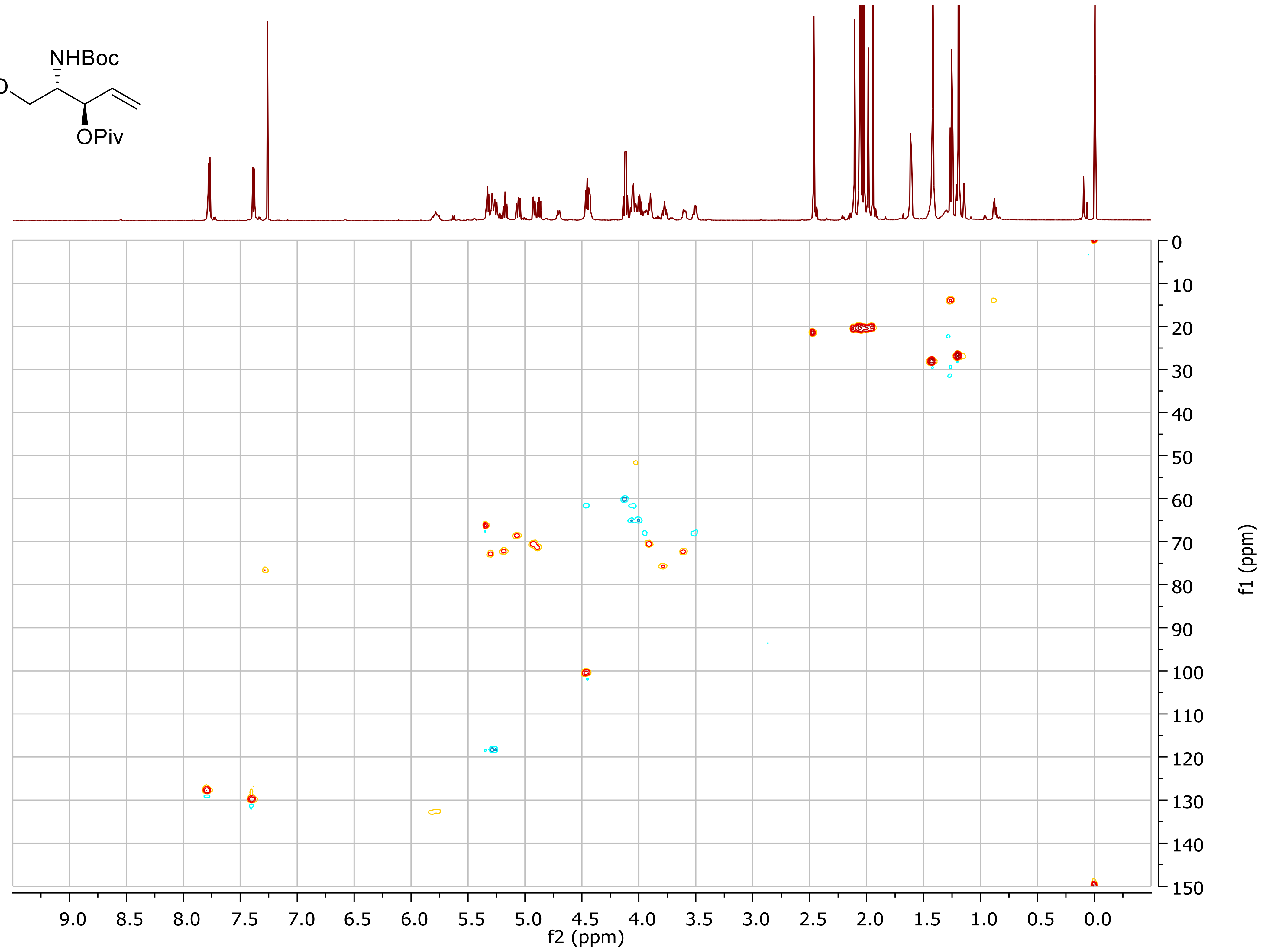

Figure S19. HR ESI-TOF-MS of compound **10**

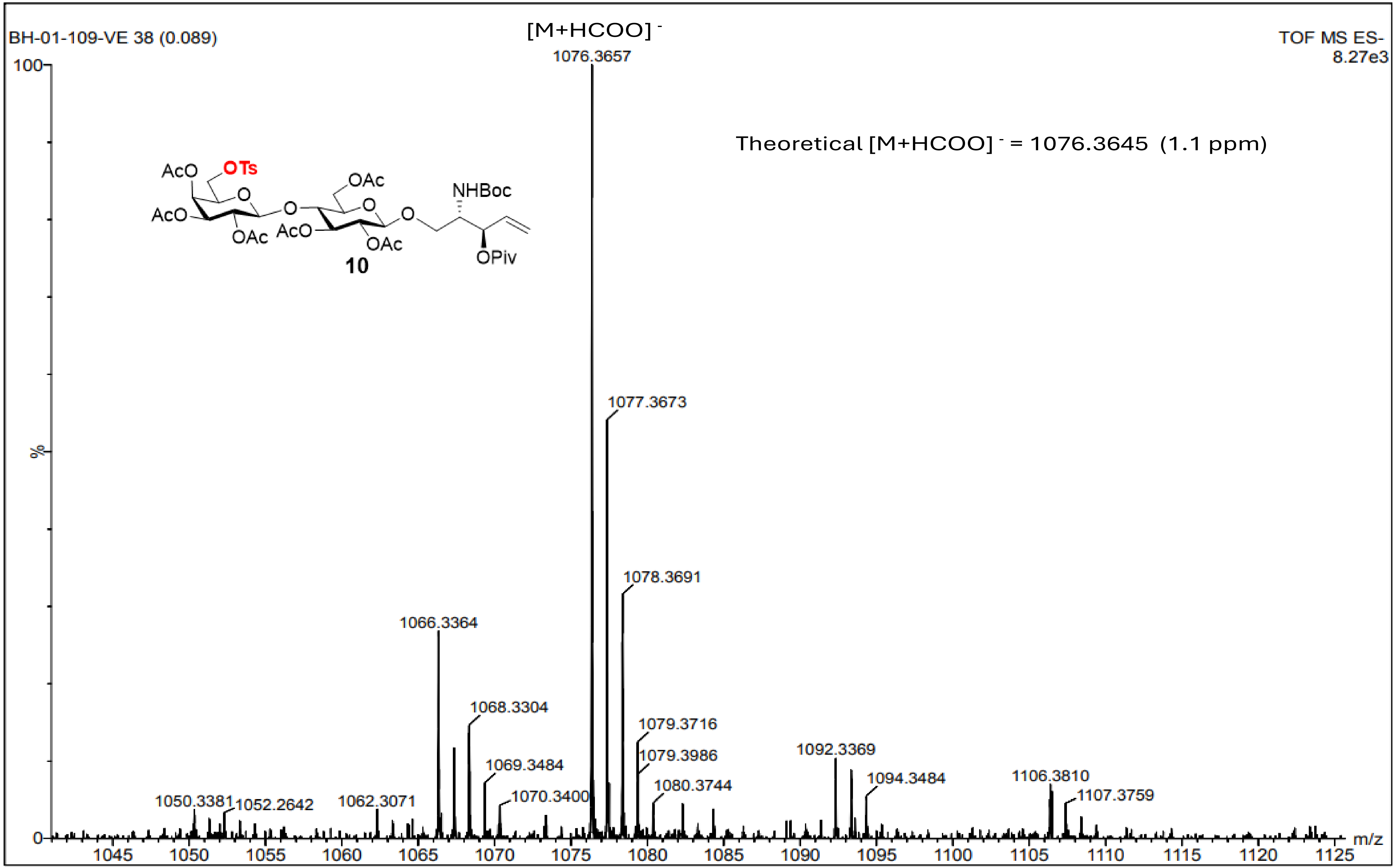

Figure S20. ^{1}H NMR of compound **12a** (400 MHz, $CDCl_3$)

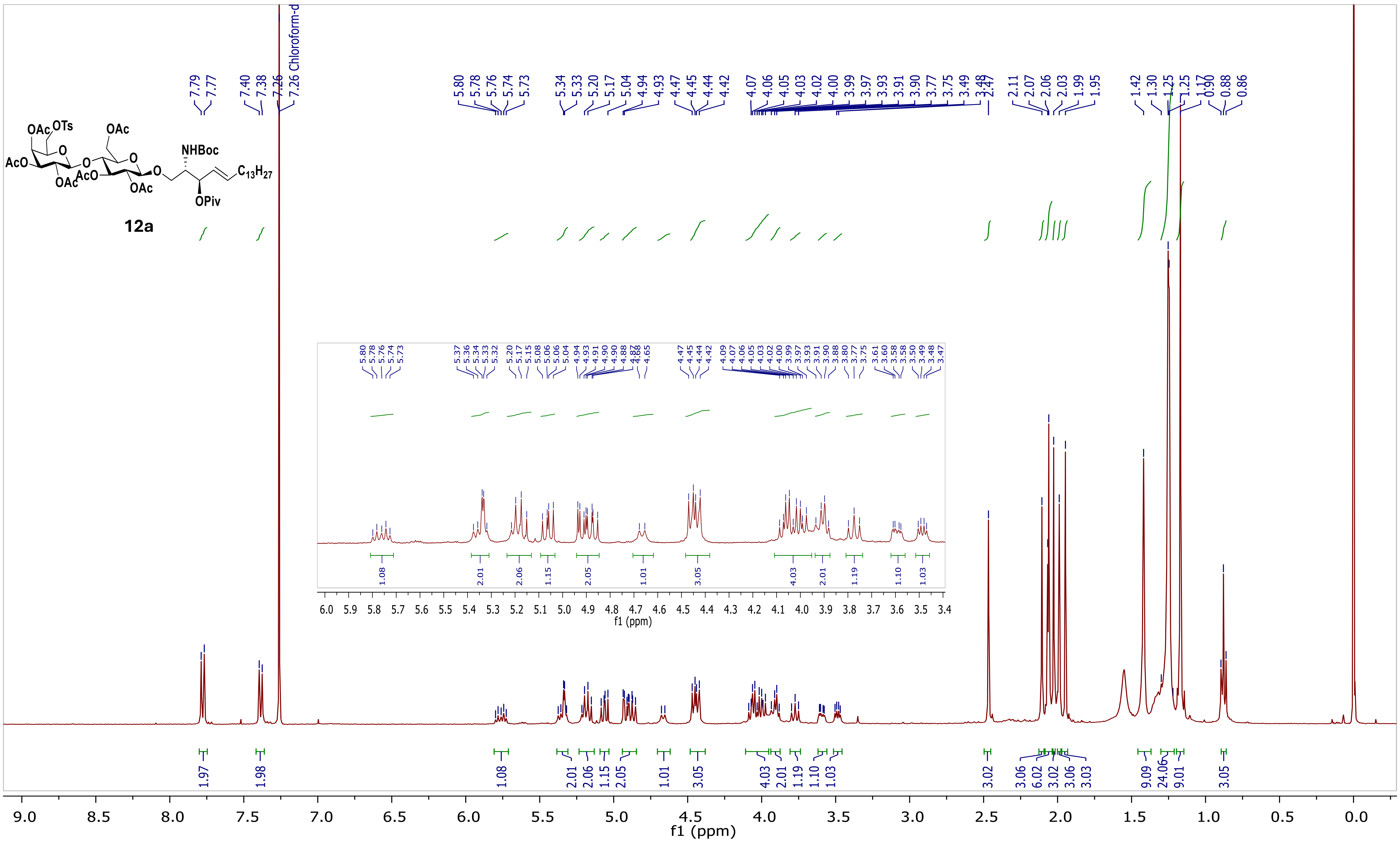

Figure S21. ^{13}C NMR of compound **12a** (101 MHz, $CDCl_3$)

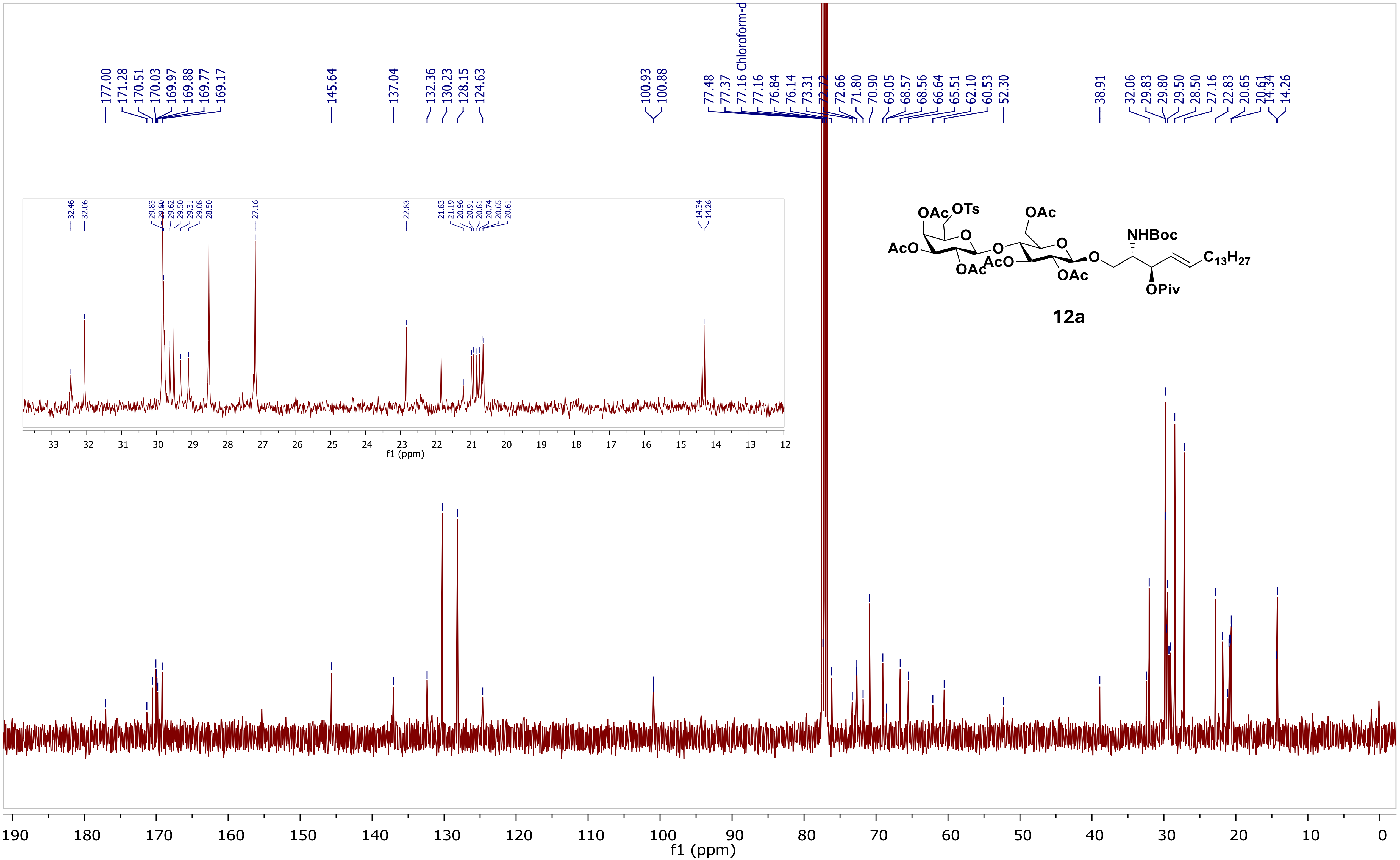

Figure S22. ^1H-^1H COSY NMR (400 MHz, $CDCl_3$) of compound **12a**

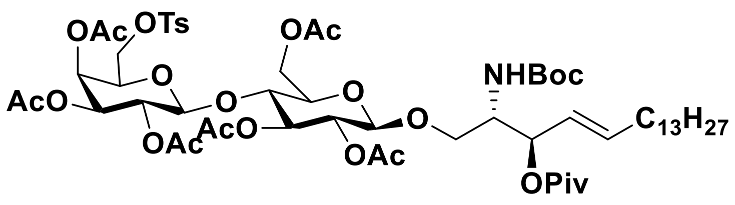

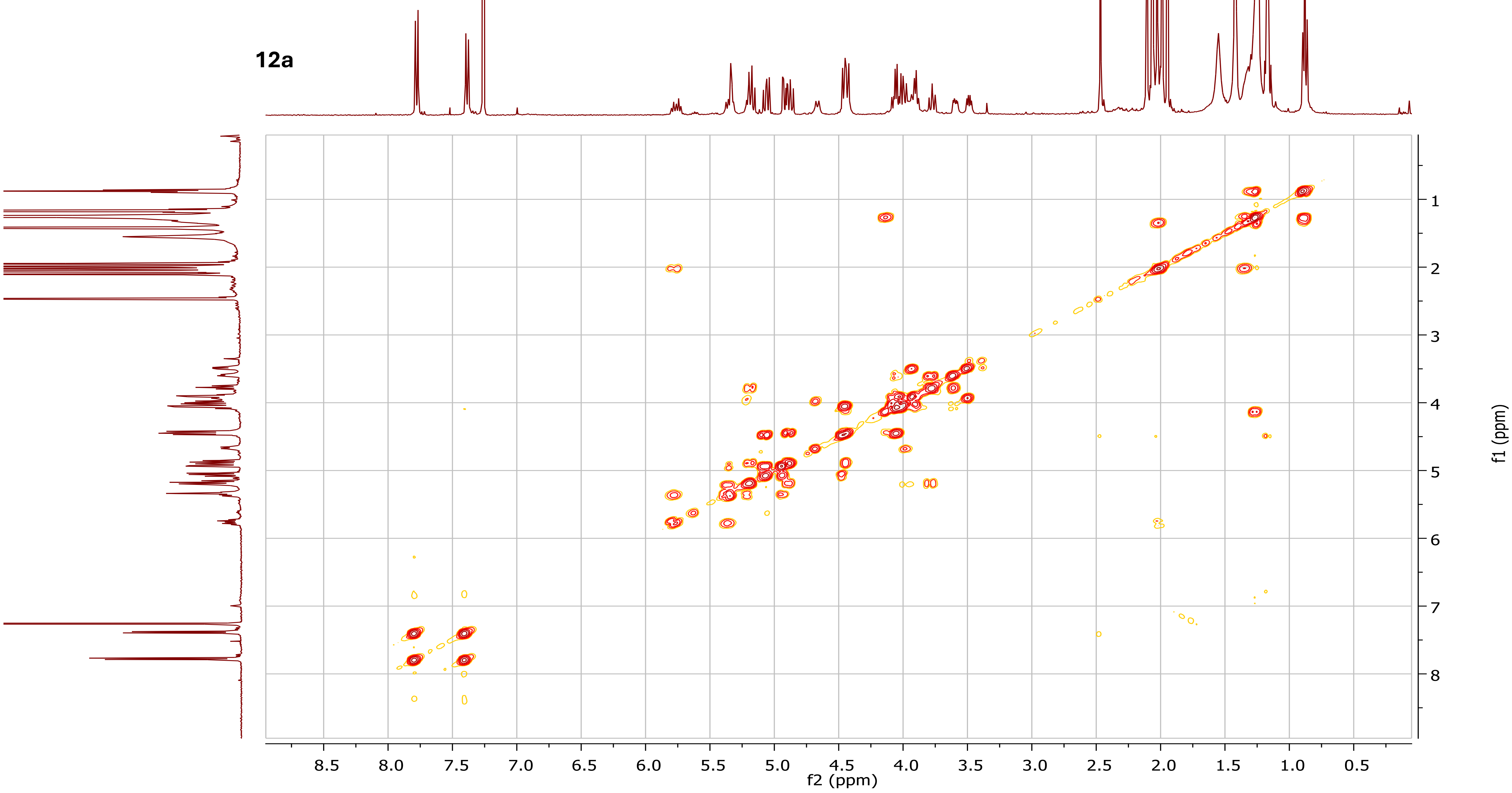

Figure S23. ^{1}H-^{13}C HSQC NMR (400/101 MHz, $CDCl_3$) of compound **12a**

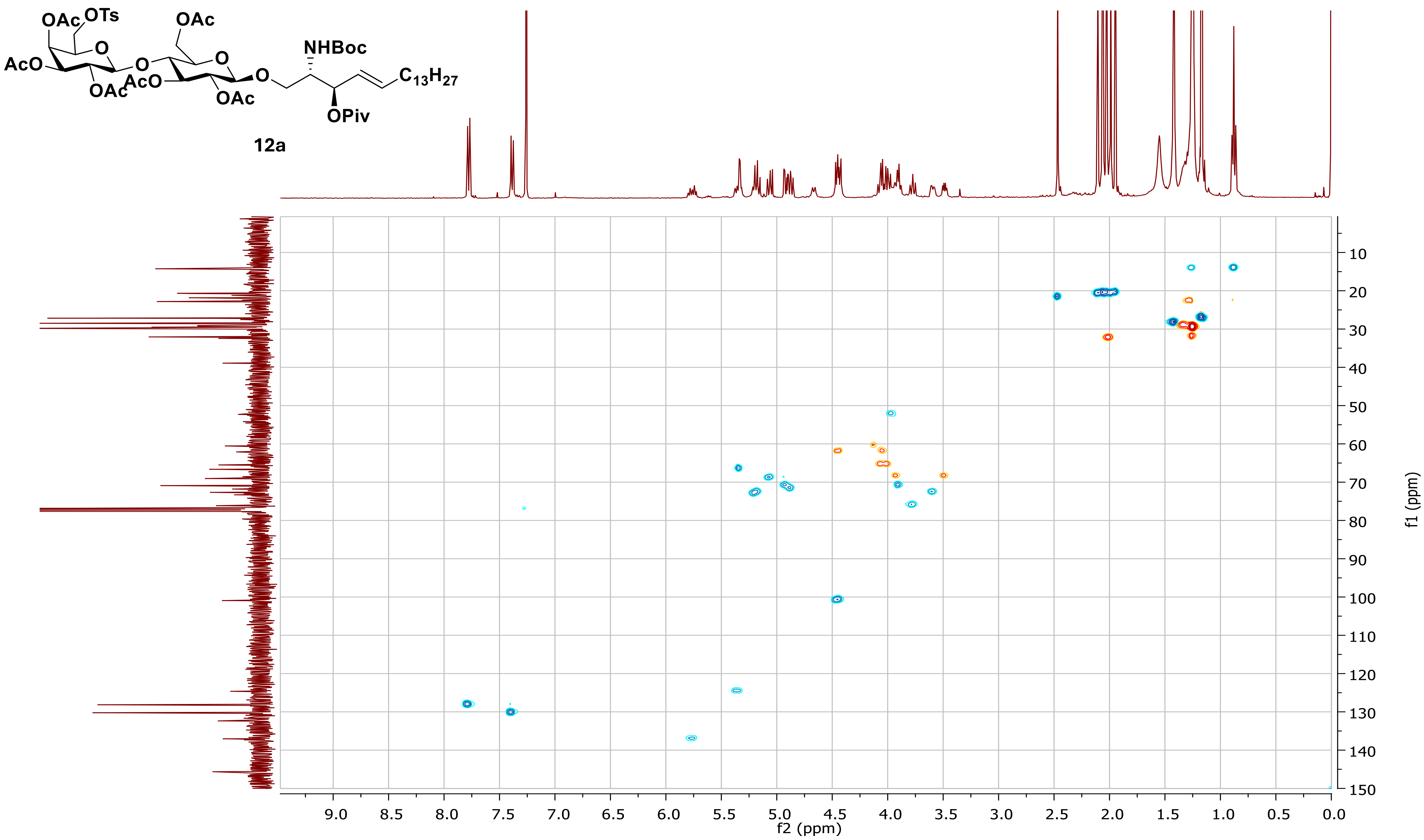

Figure S24. HR ESI-TOF-MS of compound **12a**

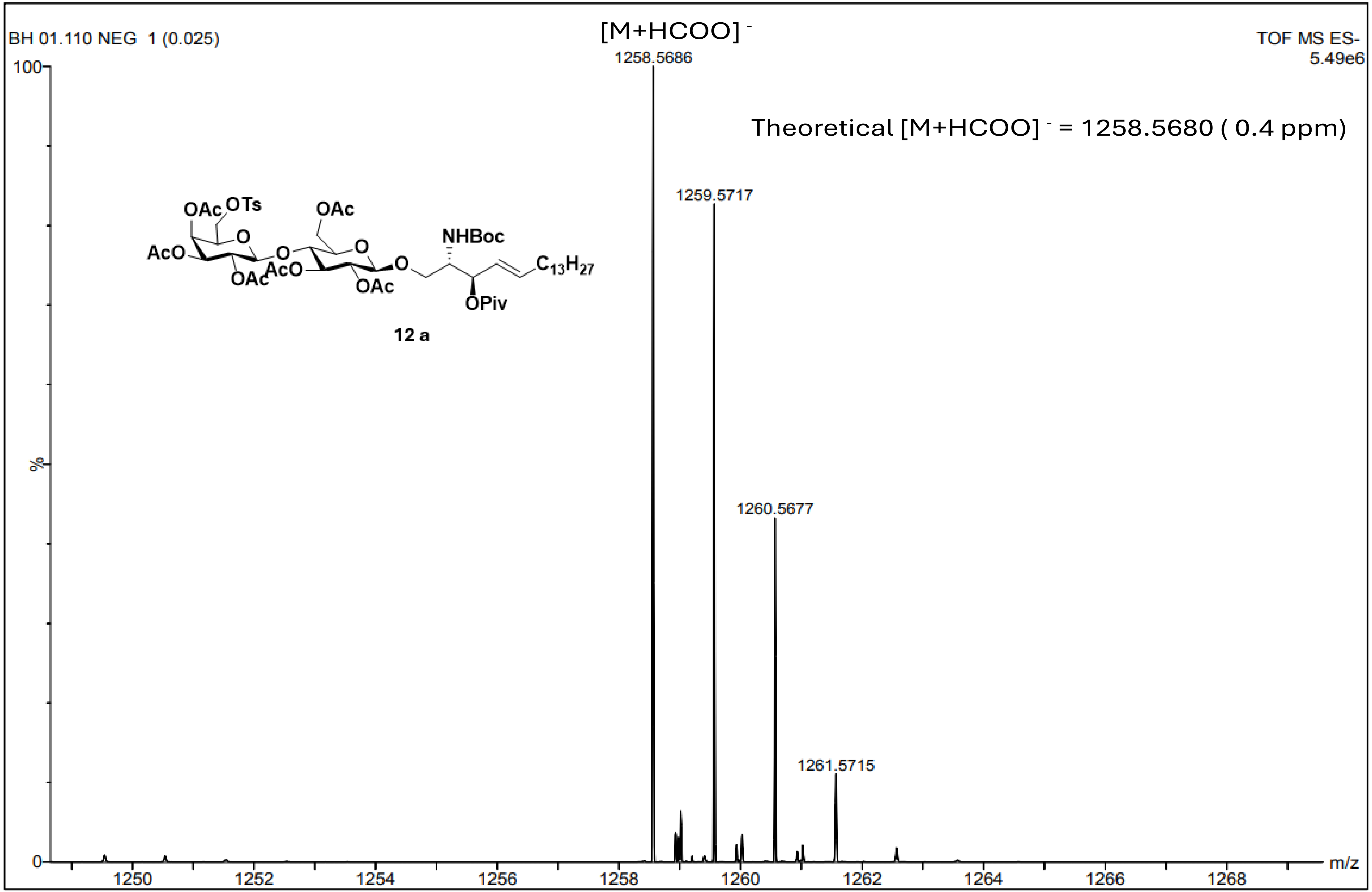

Figure S25. ^{1}H NMR of compound **12b** (600 MHz, $CDCl_3$)

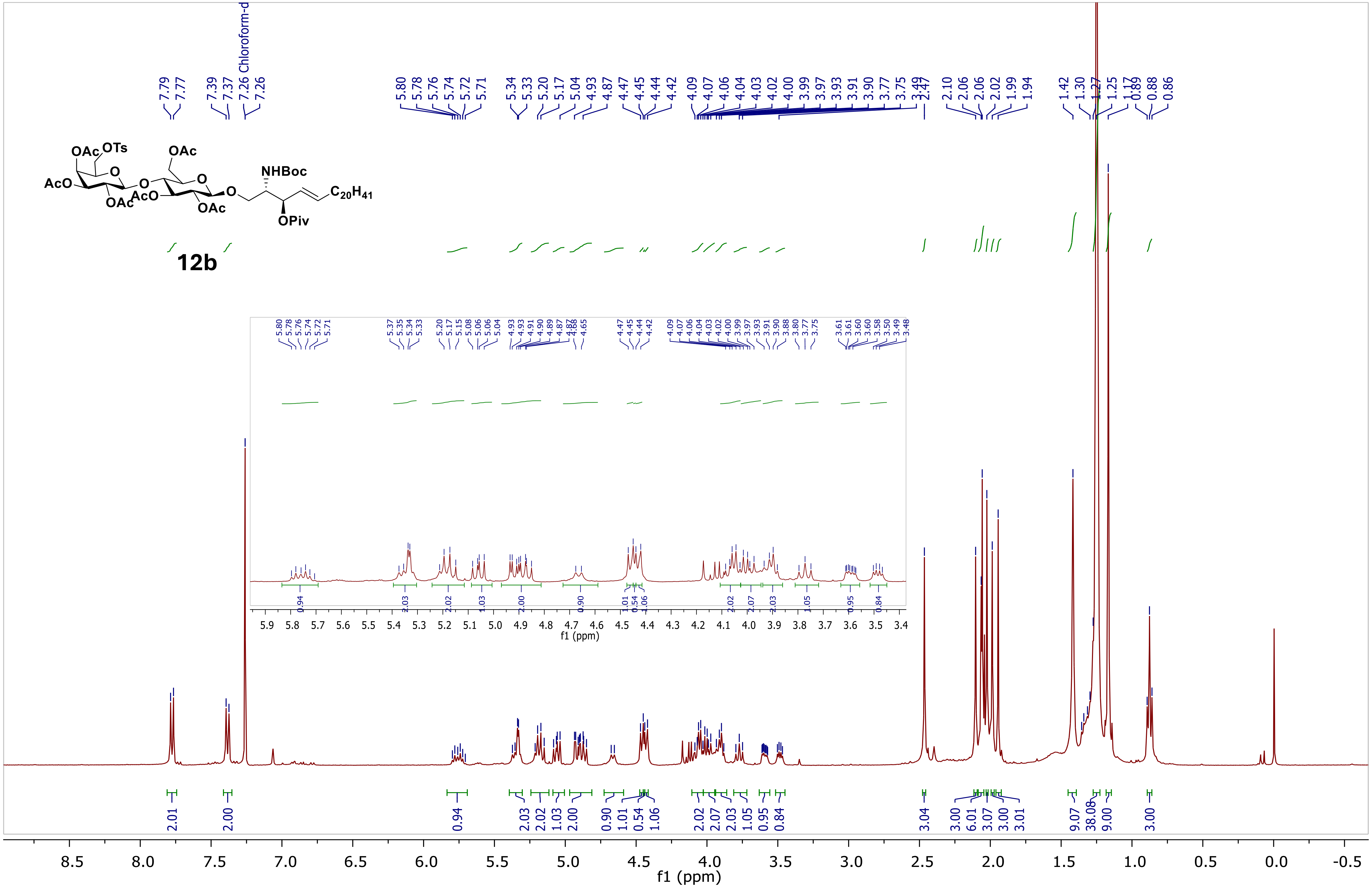

Figure S26. ^{13}C NMR of compound **12b** (151 MHz, $CDCl_3$)

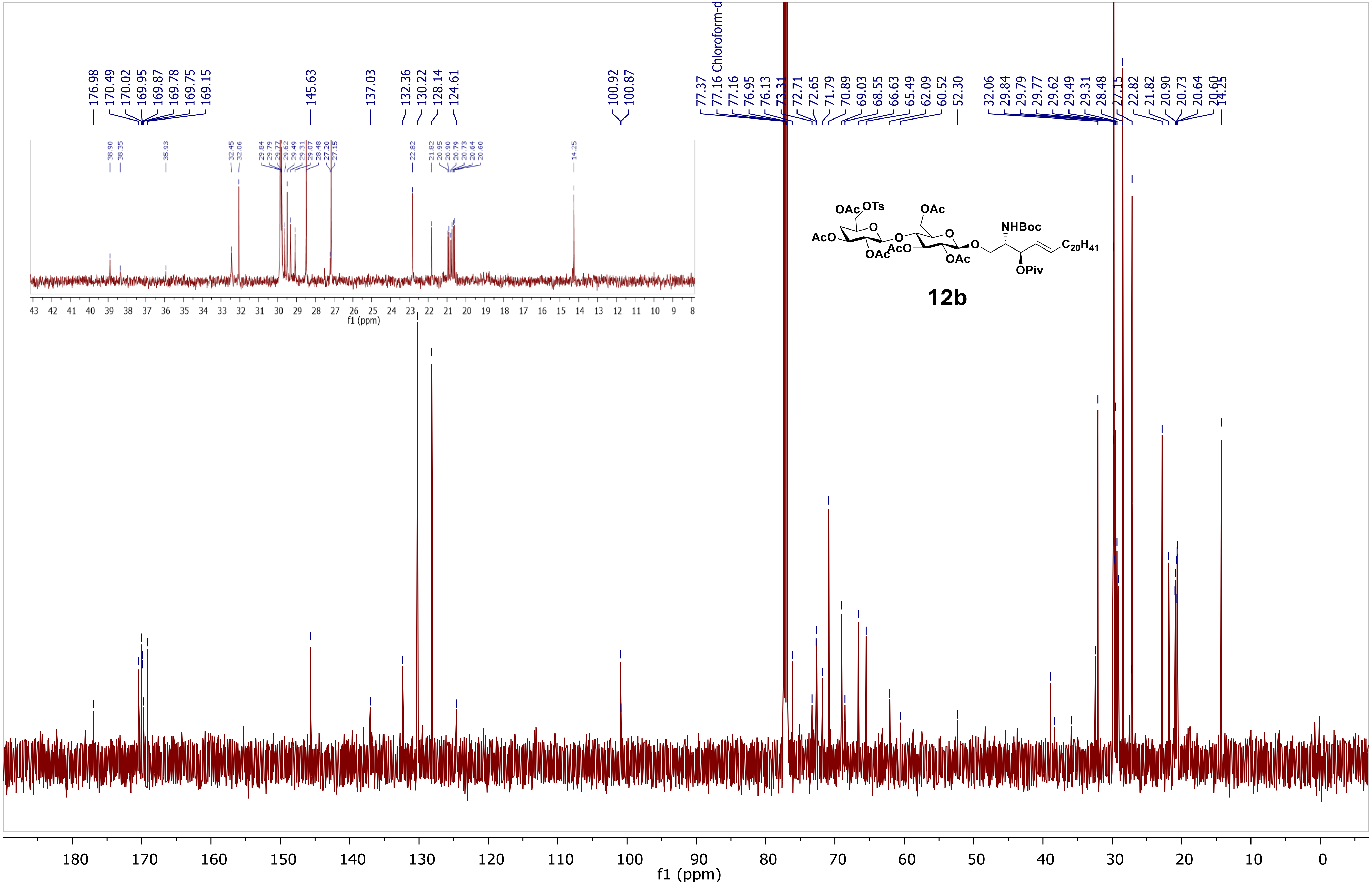

Figure S27. ^1H-^1H COSY NMR (600 MHz, $CDCl_3$) of compound **12b**

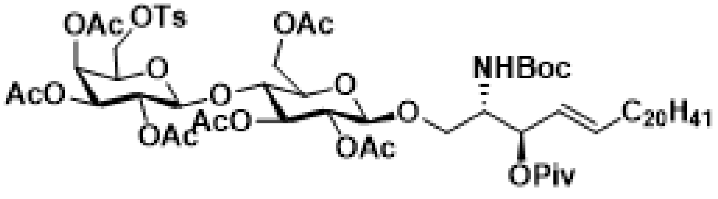

12b

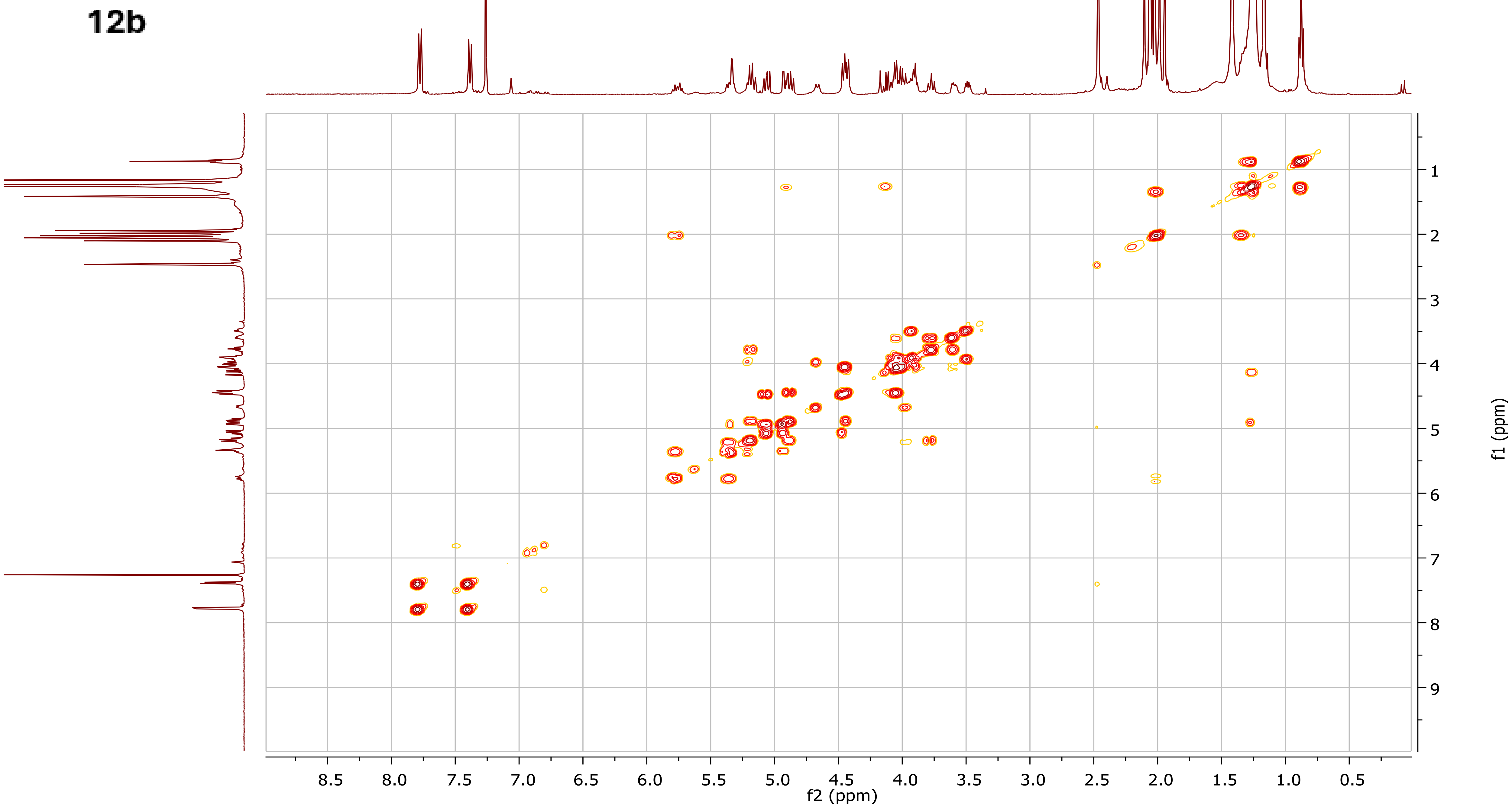

Figure S28. ^{1}H-^{13}C HSQC NMR (600/151 MHz, $CDCl_3$) of compound **12b**

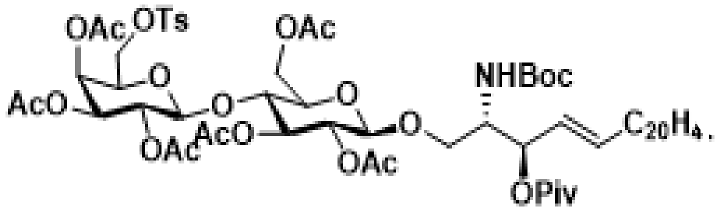

12b

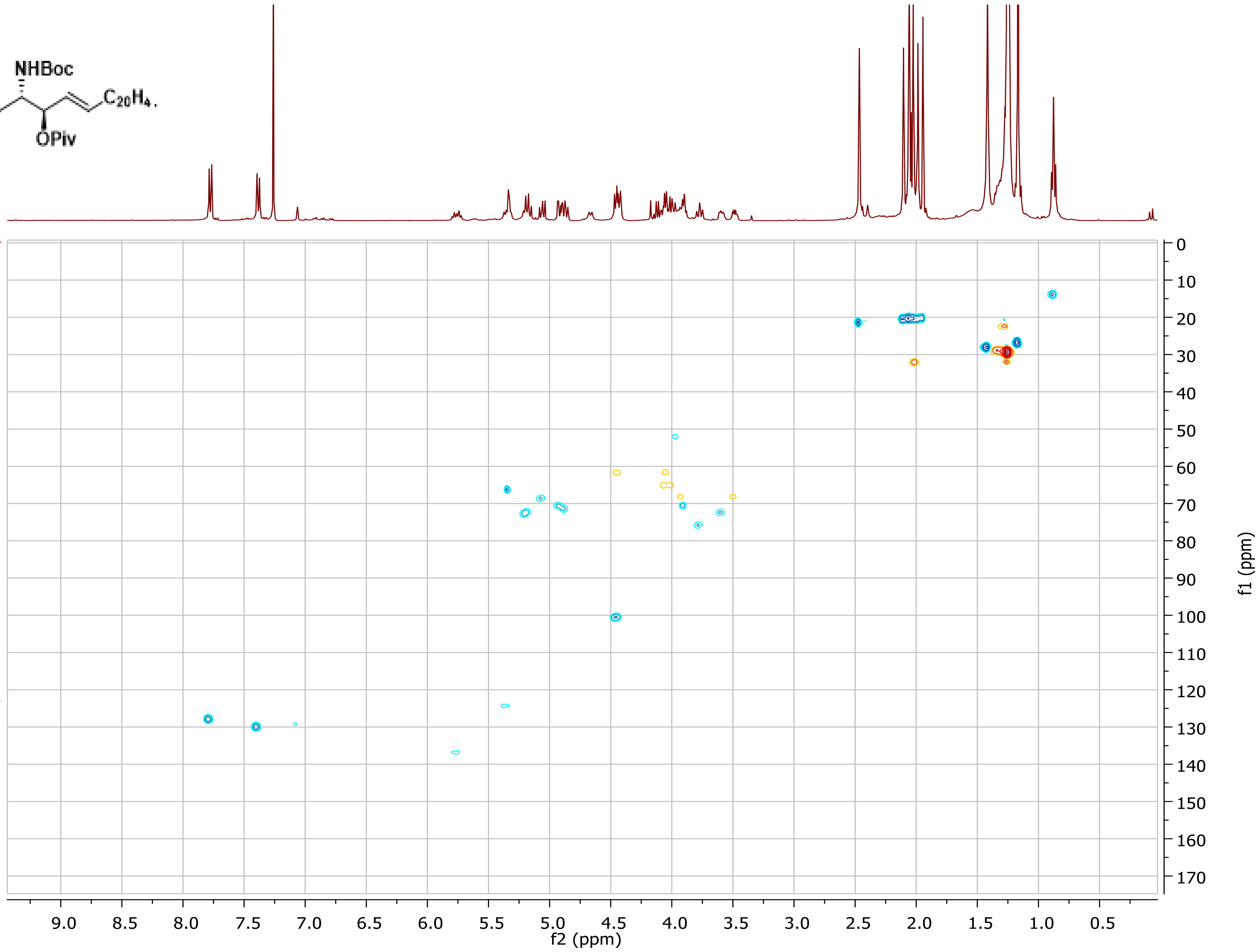

Figure S29. HR ESI-TOF-MS of compound **12b**

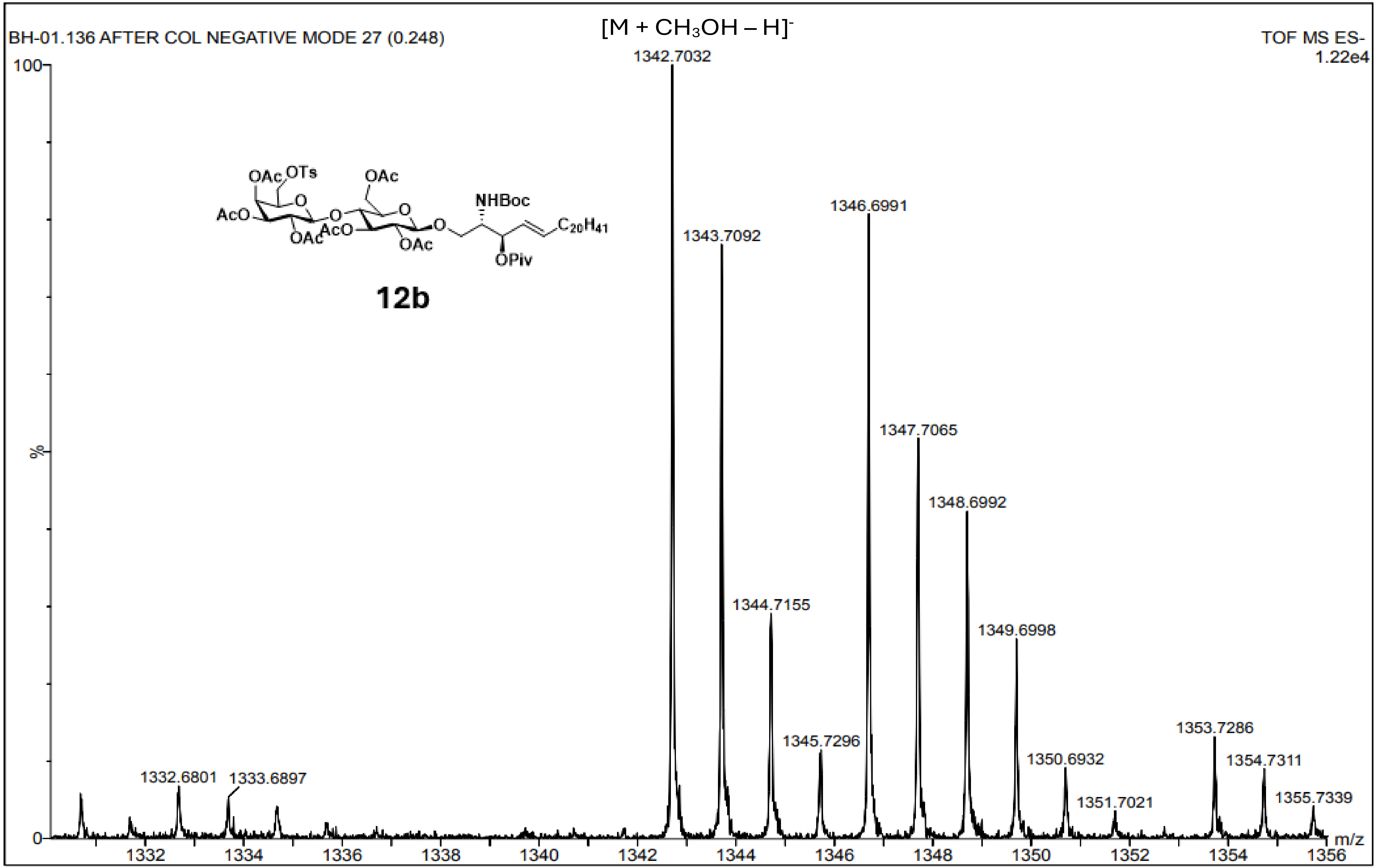

Figure S30. ^{1}H NMR of compound **13a** (600 MHz, $CDCl_3$)

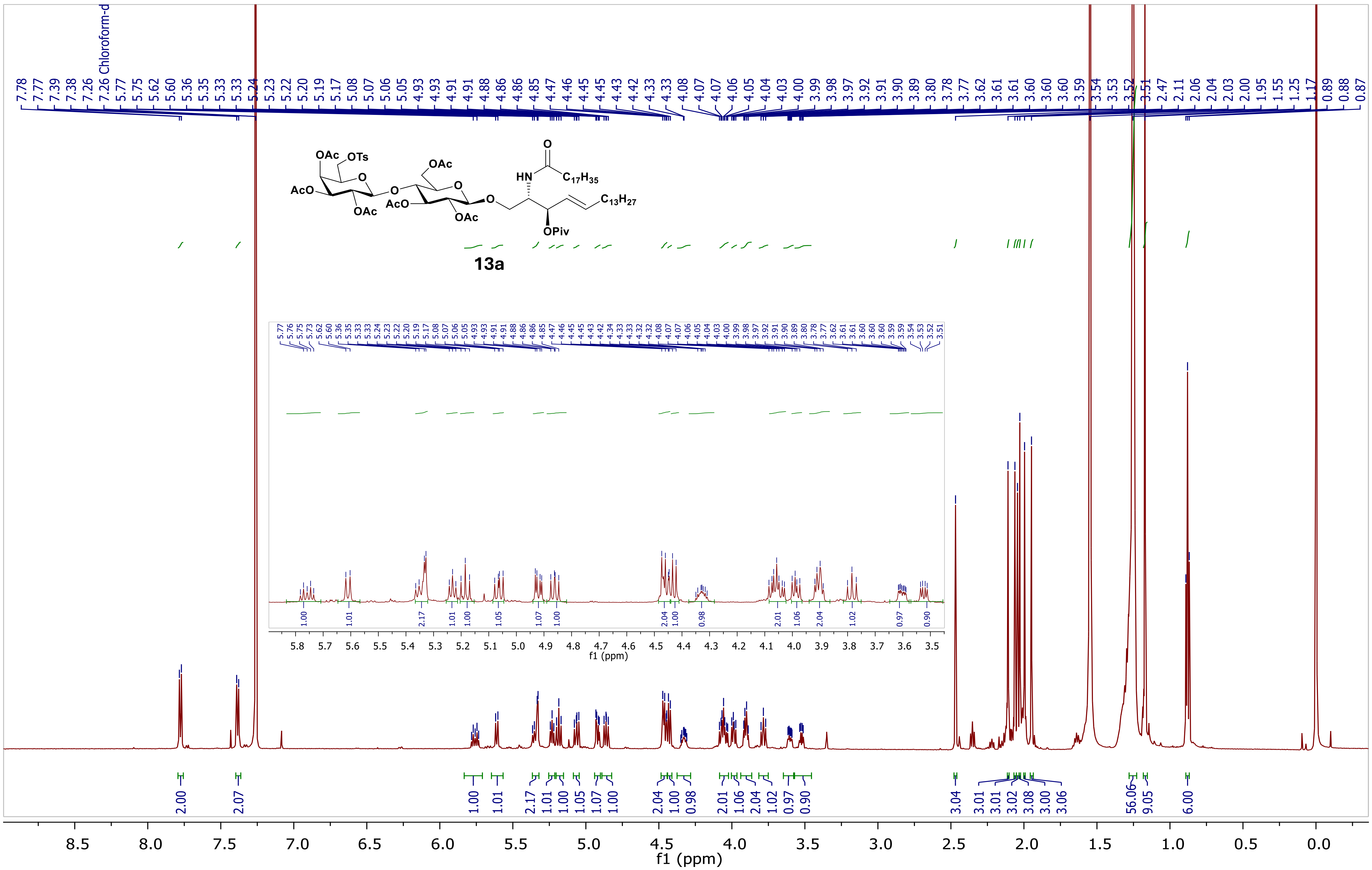

Figure S31. ^{13}C NMR of compound **13a** (151 MHz, $CDCl_3$)

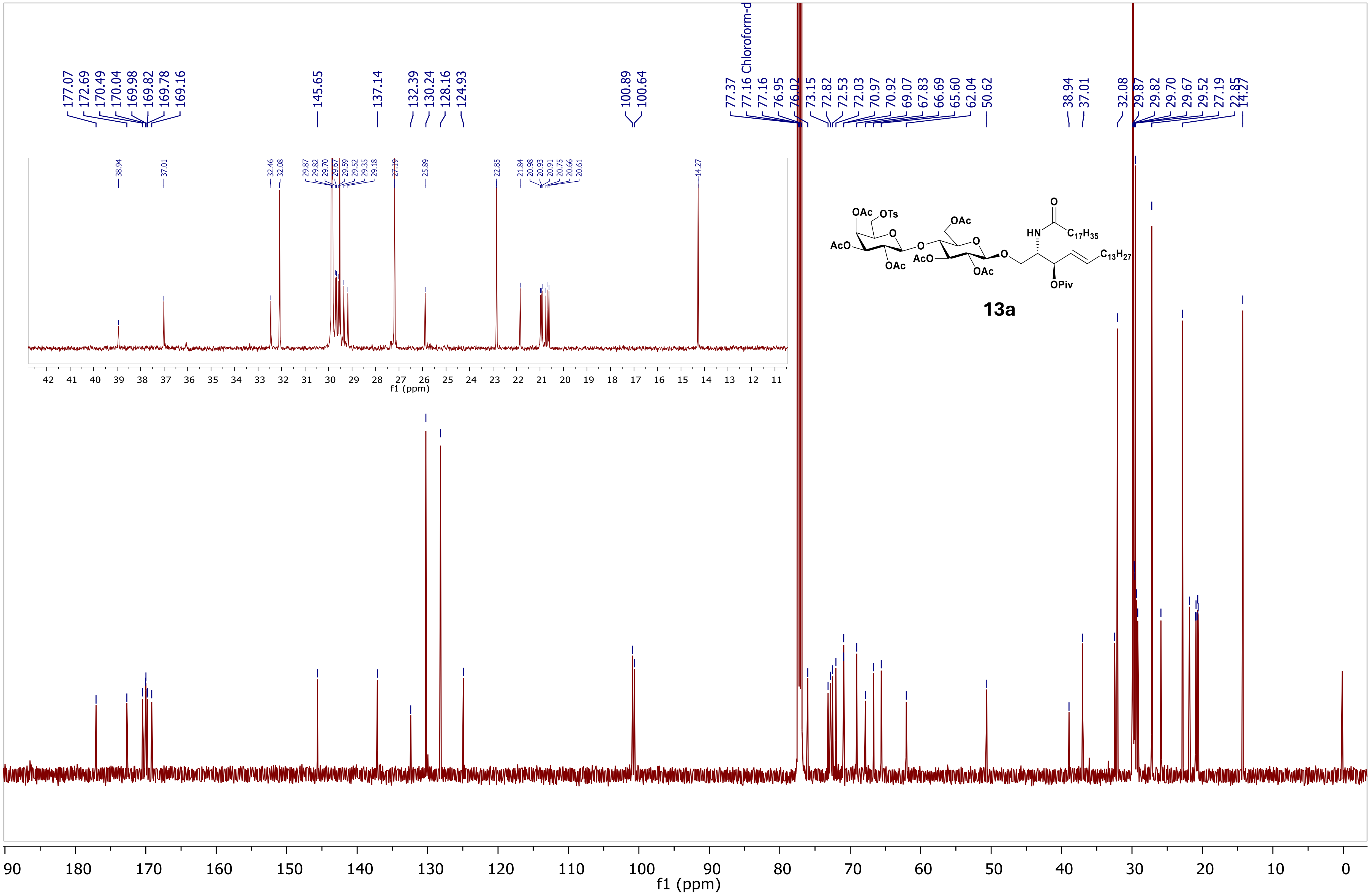

Figure S32. ^{1}H-^{1}H COSY NMR (600 MHz, $CDCl_3$) of compound **13a**

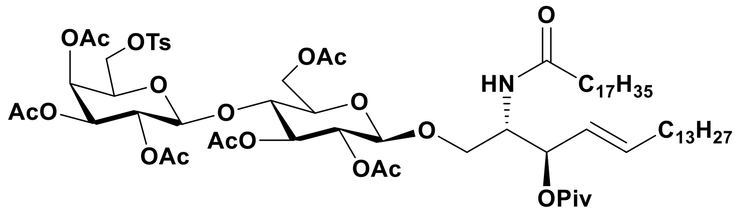

13a

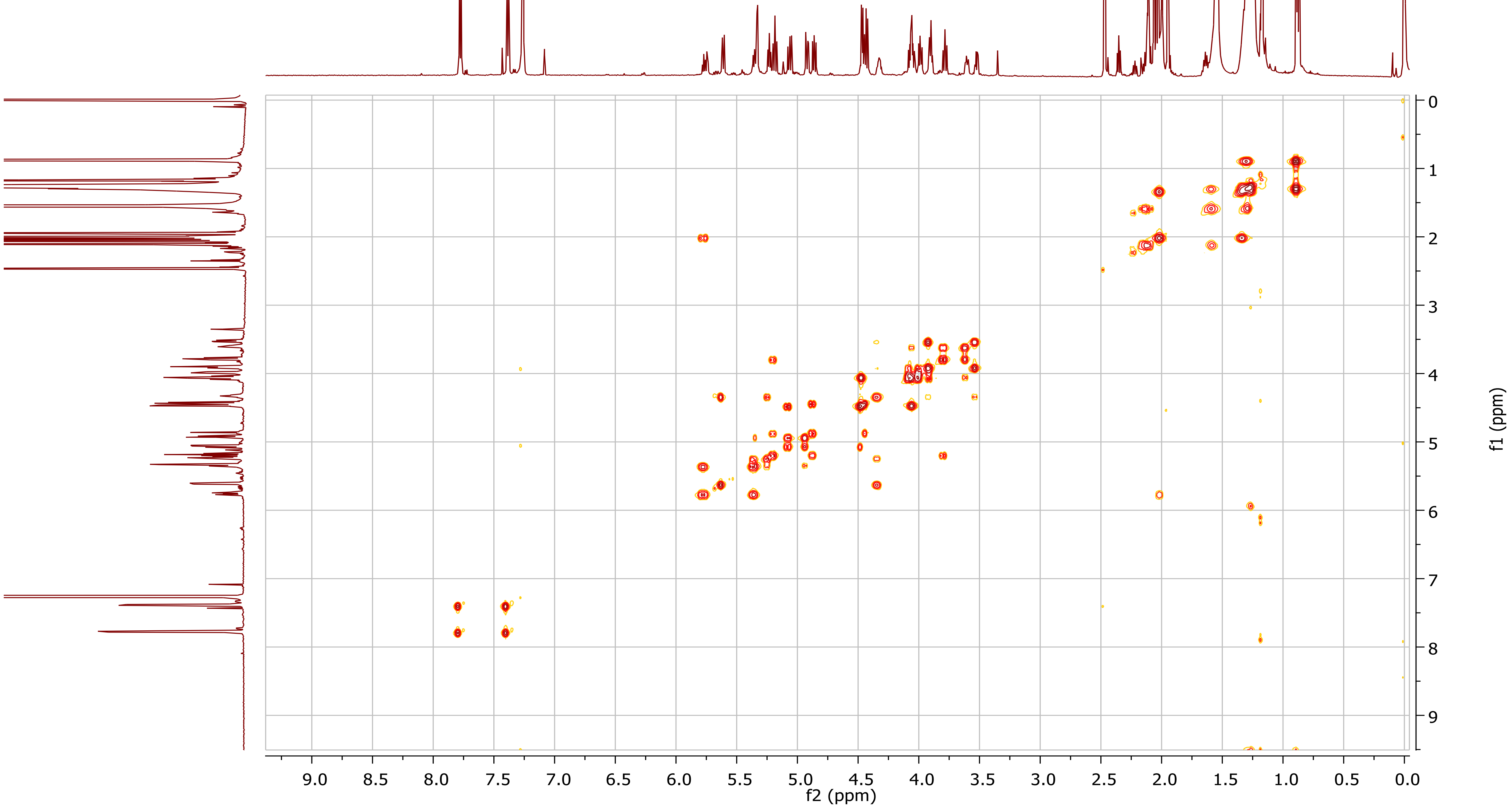

Figure S33. ^{1}H-^{13}C HSQC NMR (600/151 MHz, $CDCl_3$) of compound **13a**

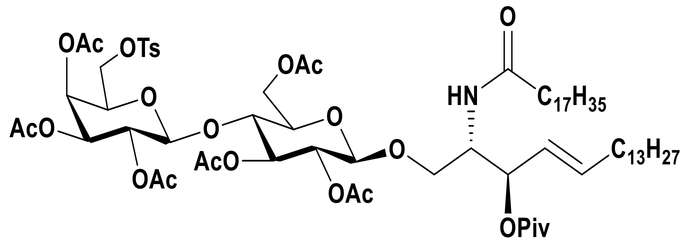

13a

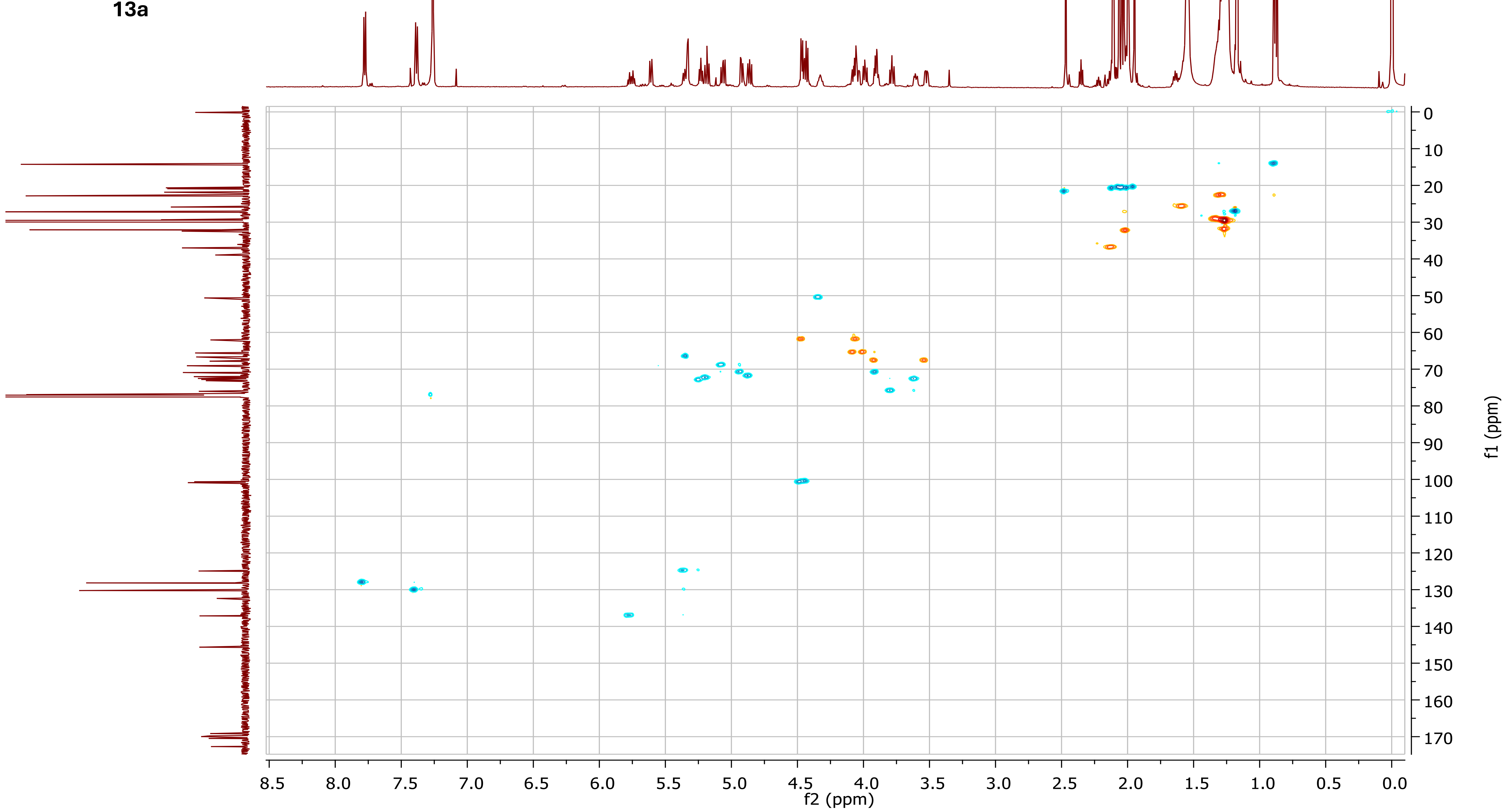

Figure S34. HR ESI-TOF-MS of compound **13a**

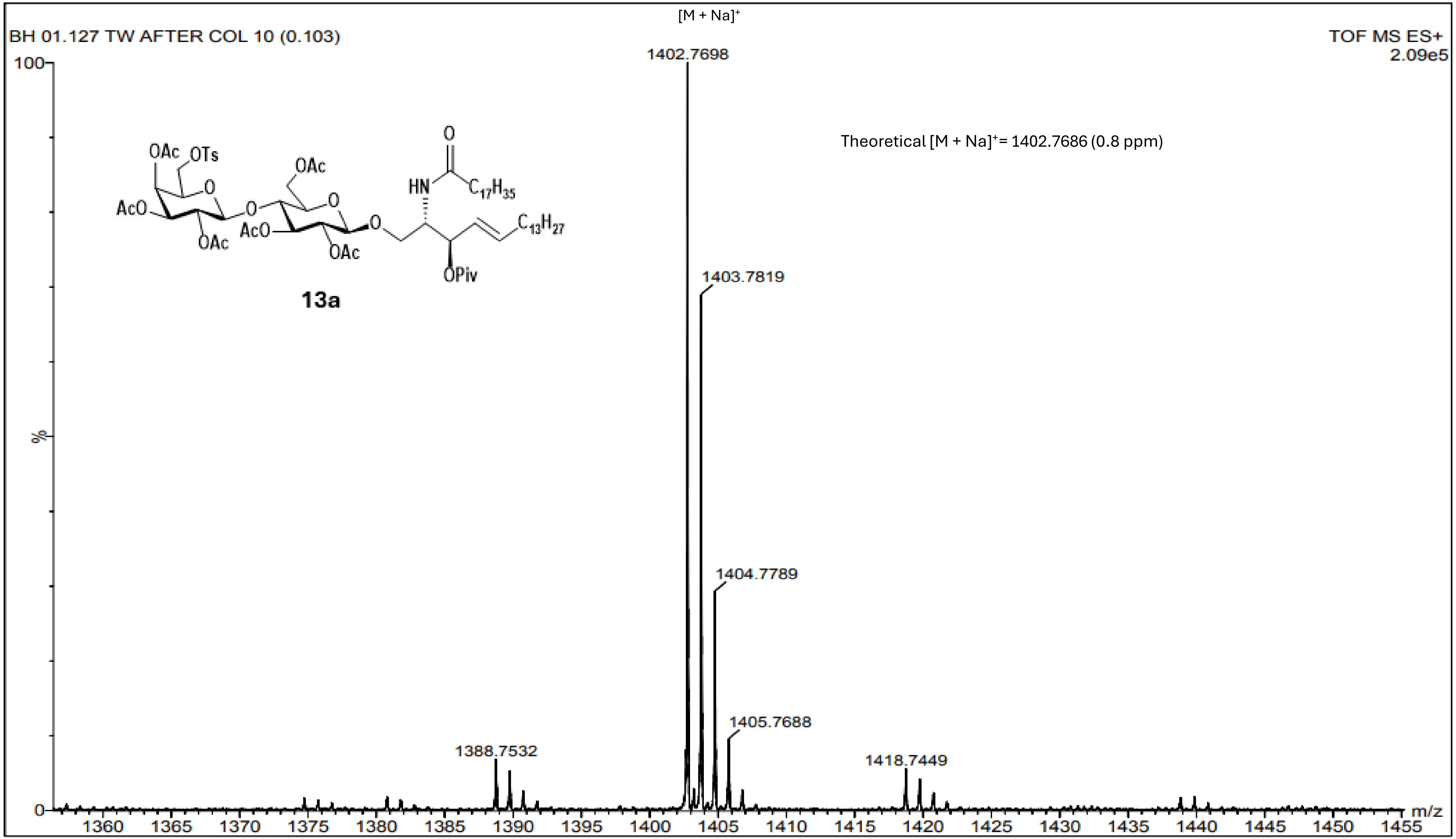

Figure S35. ^{1}H NMR of compound **13b** (600 MHz, $CDCl_3$)

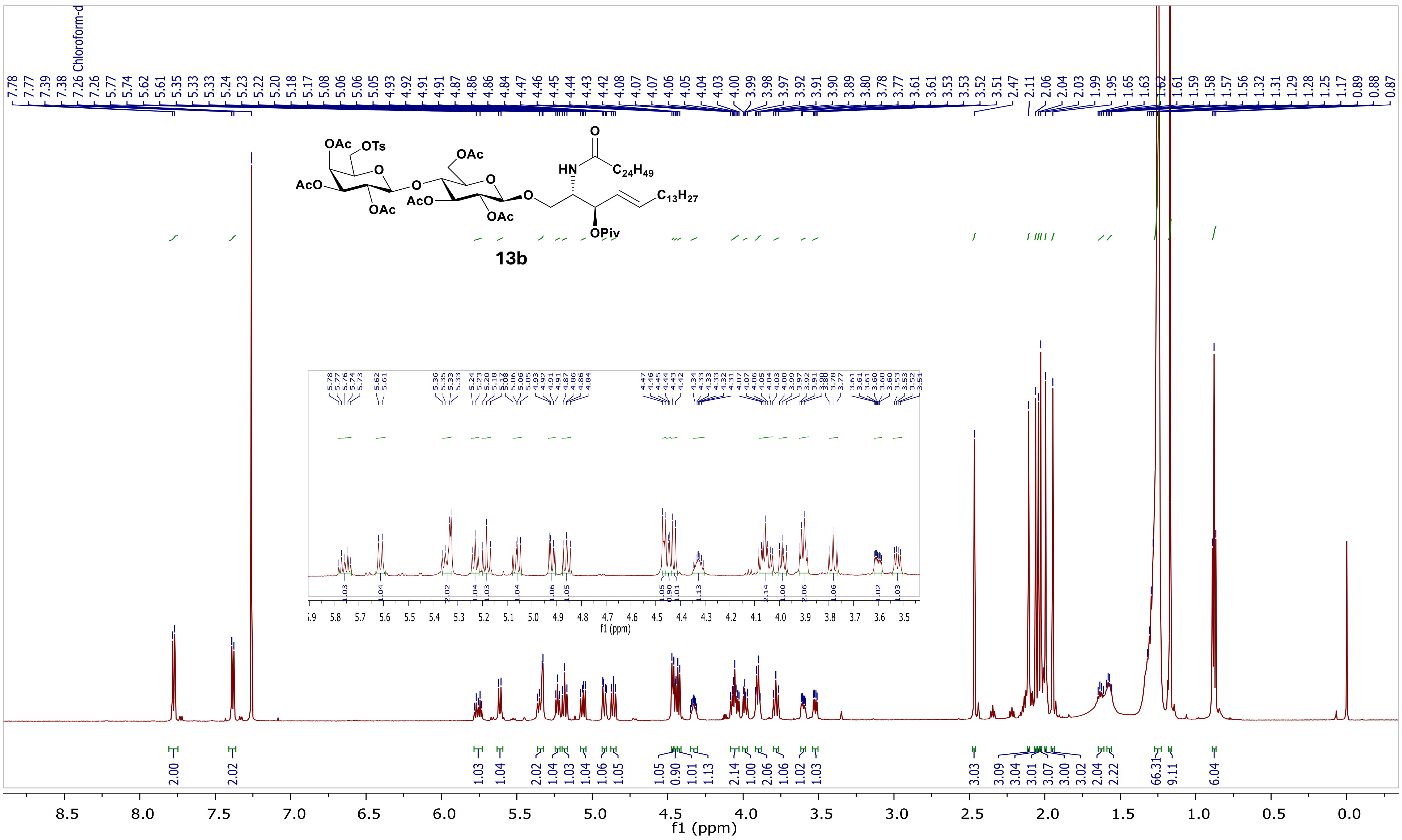

Figure S36. ^{13}C NMR of compound **13b** (151 MHz, $CDCl_3$)

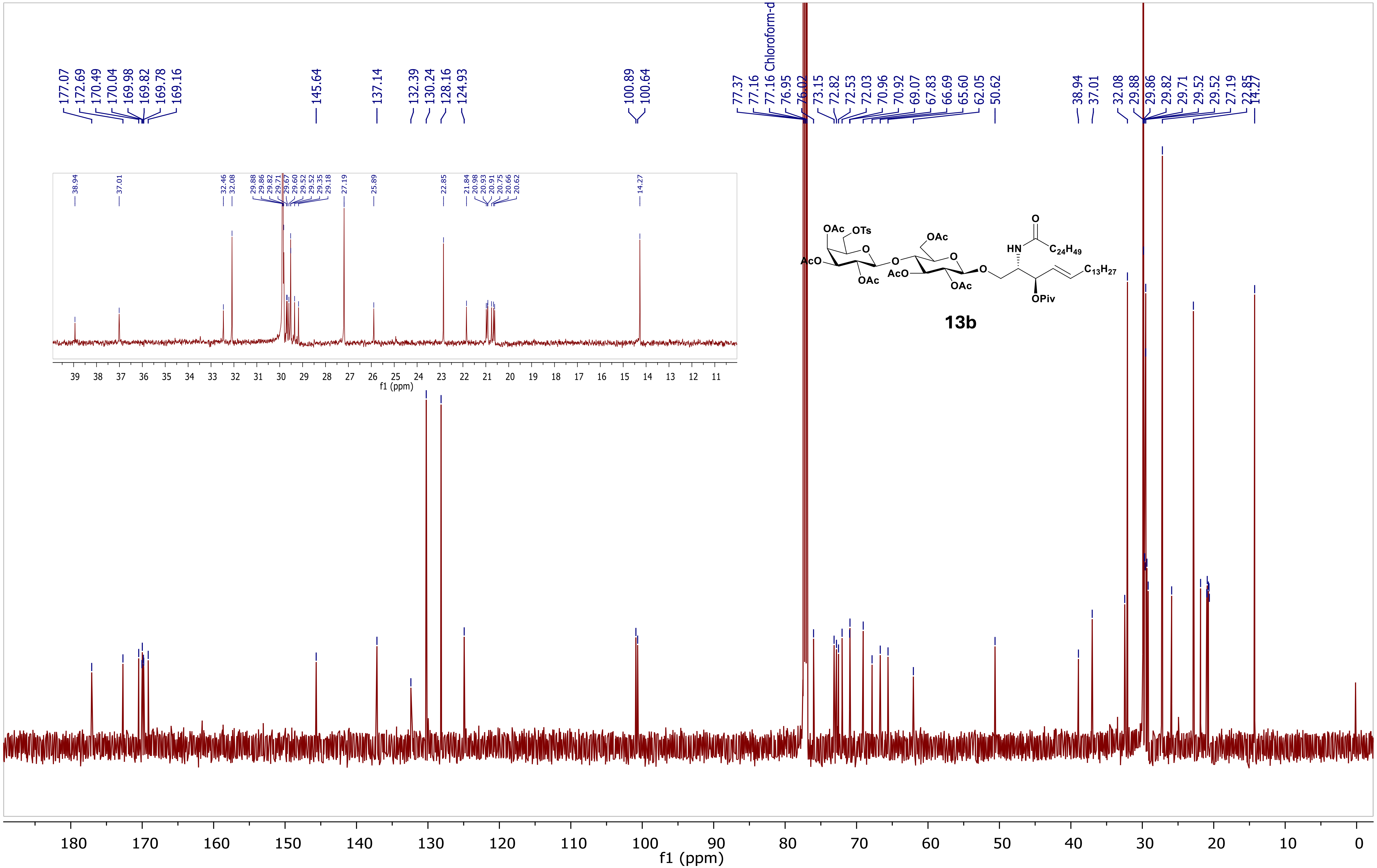

Figure 37. ^{1}H-^{1}H COSY NMR (600 MHz, $CDCl_3$) of compound **13b**

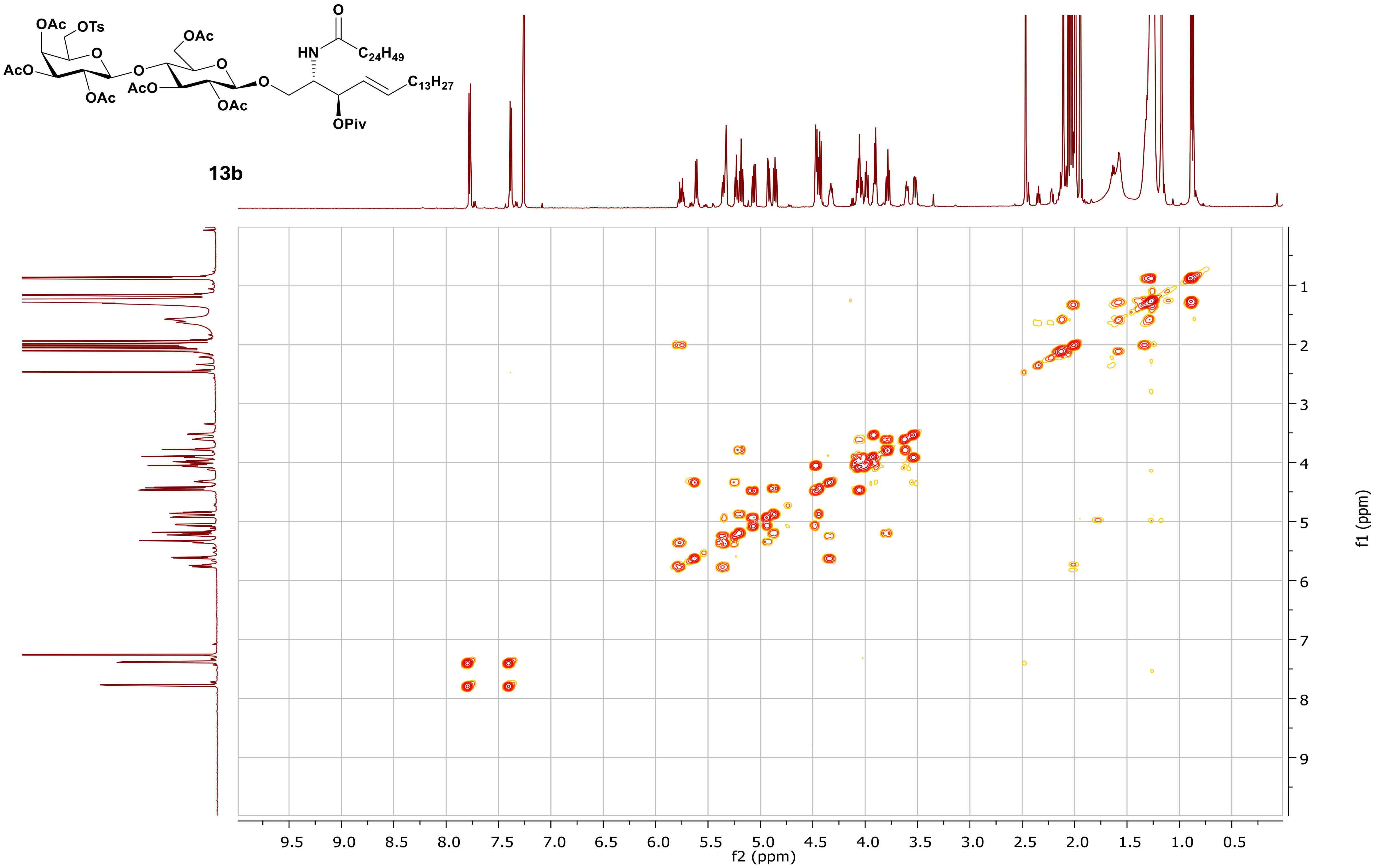

Figure S38. ^{1}H-^{13}C HSQC NMR (600/151 MHz, $CDCl_3$) of compound **13b**

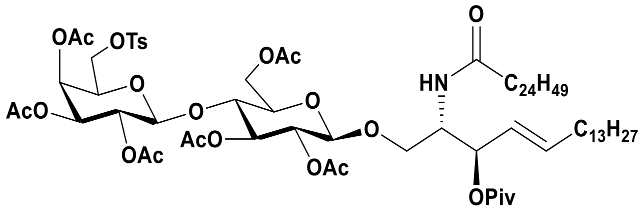

13b

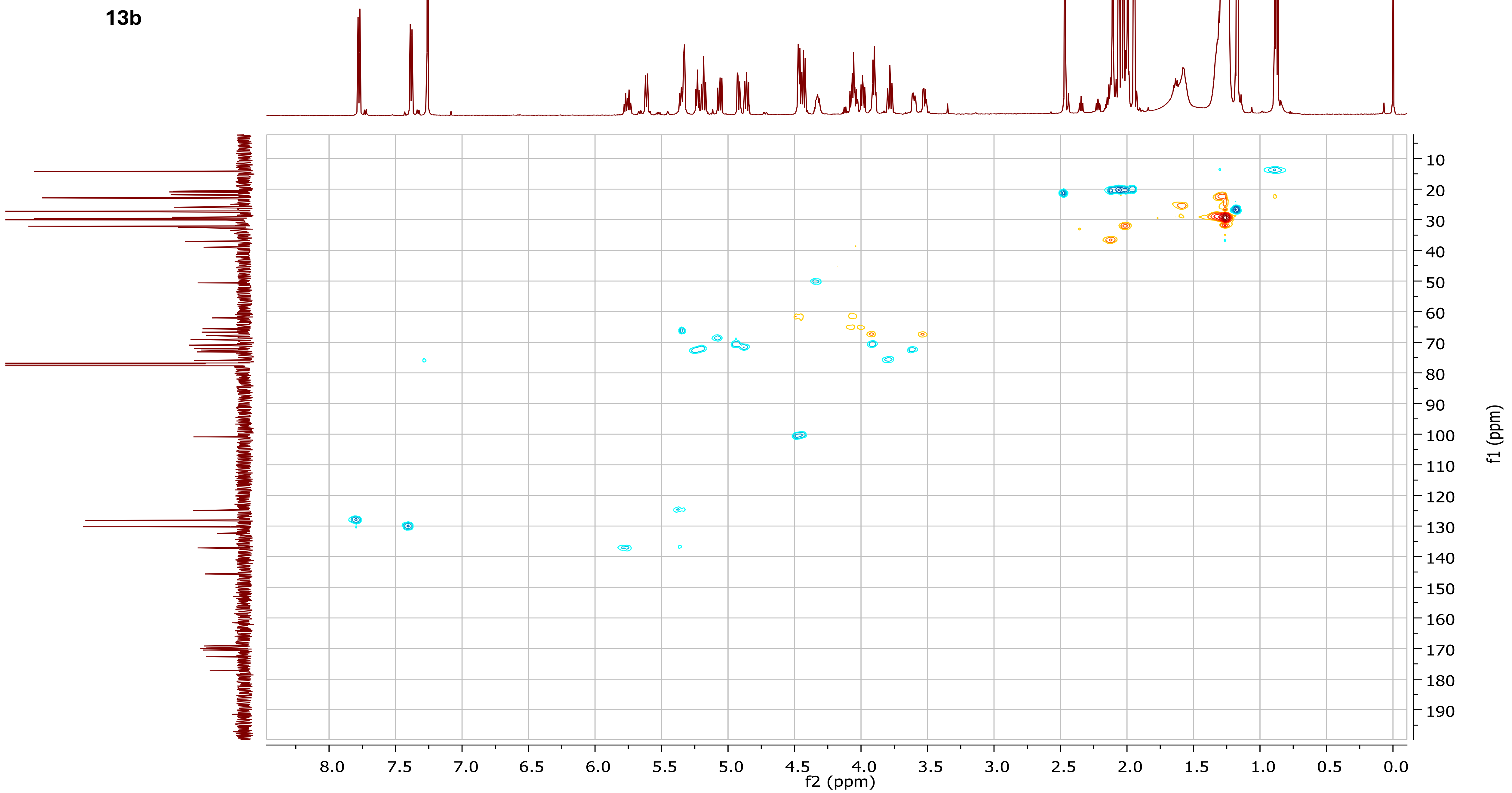

Figure S39. HR ESI-TOF-MS of compound **13b**

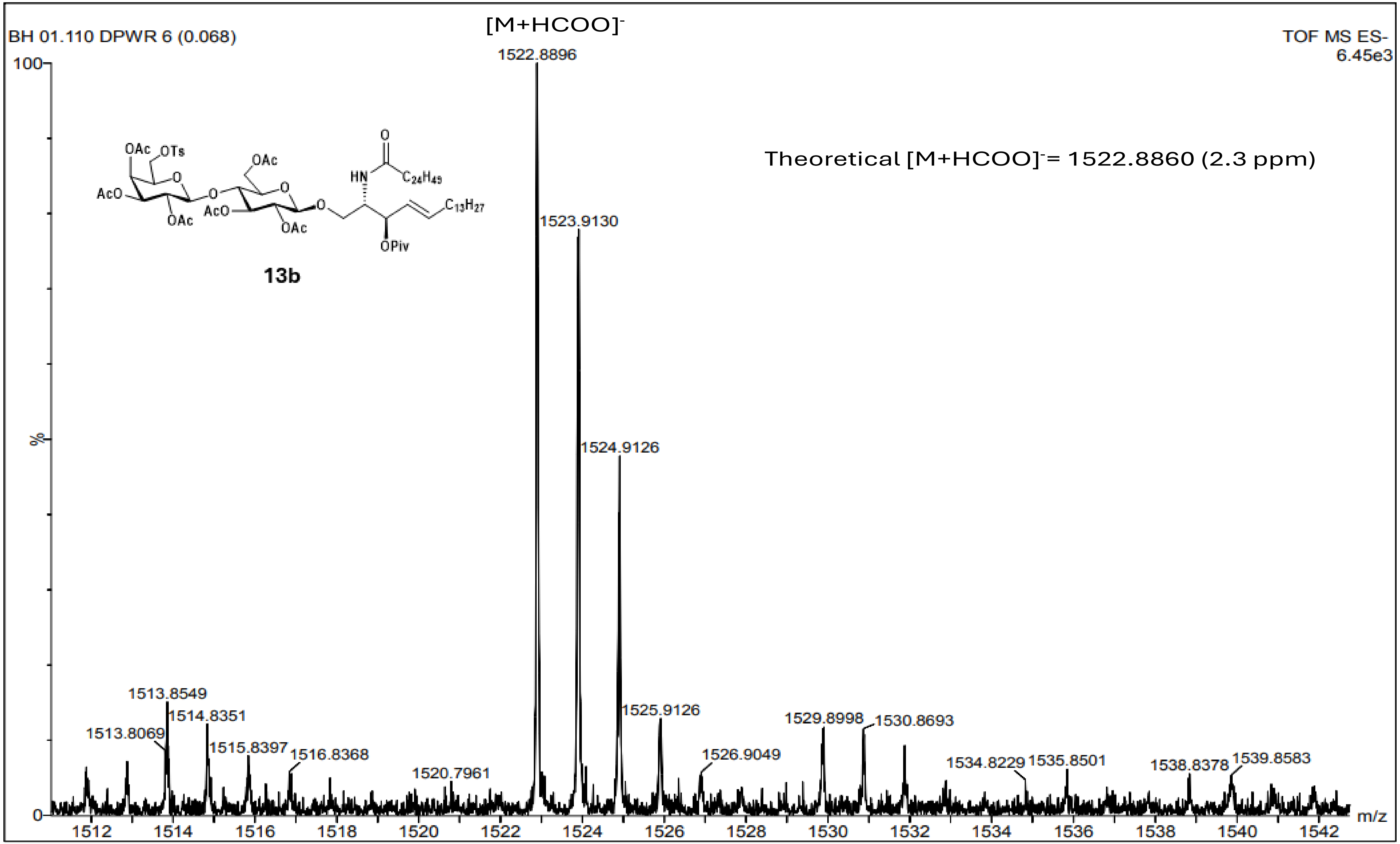

Figure S40. ^{1}H NMR of compound **13c** (600 MHz, $CDCl_3$)

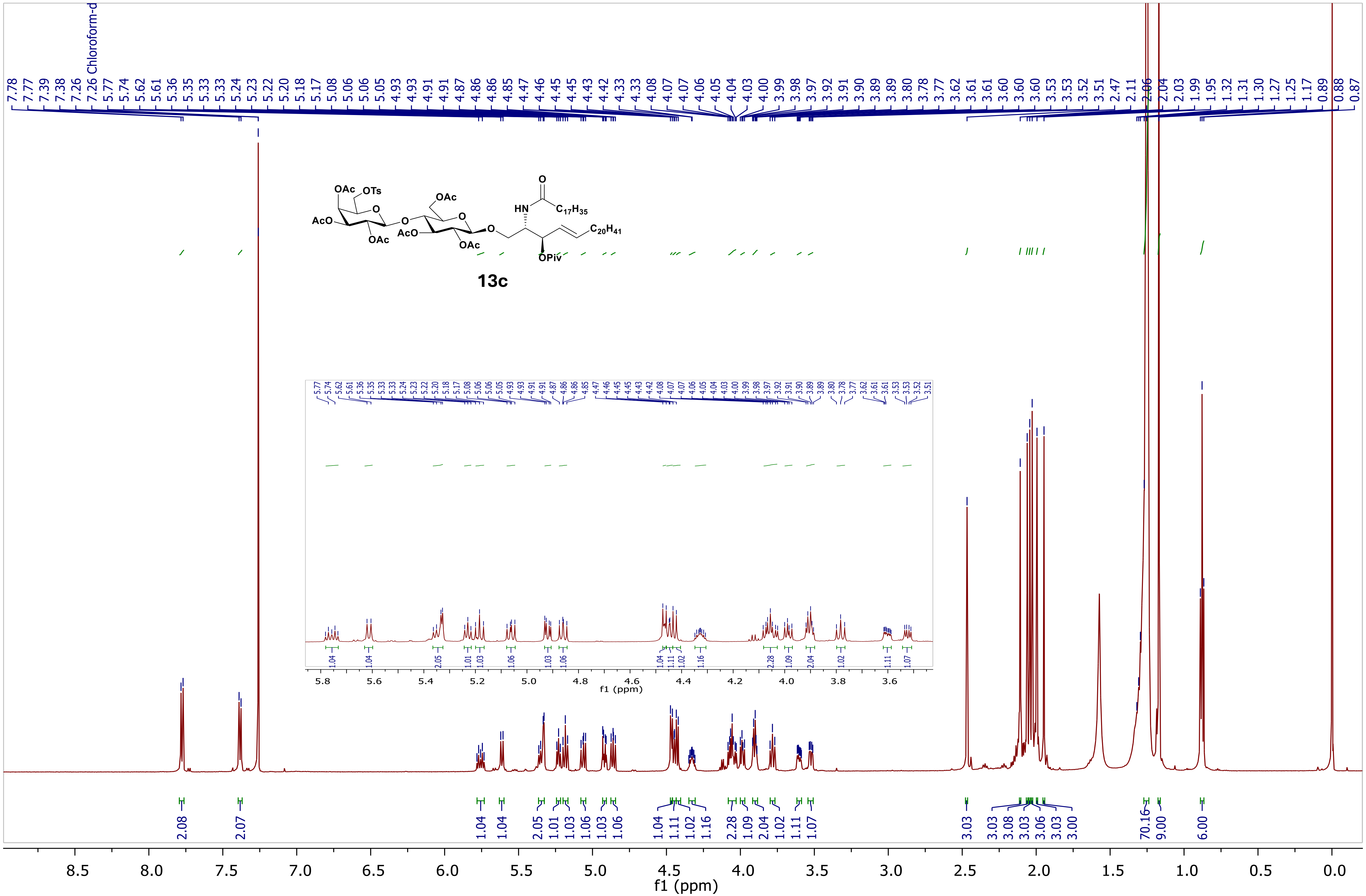

Figure S41. ^{13}C NMR of compound **13c** (151 MHz, $CDCl_3$)

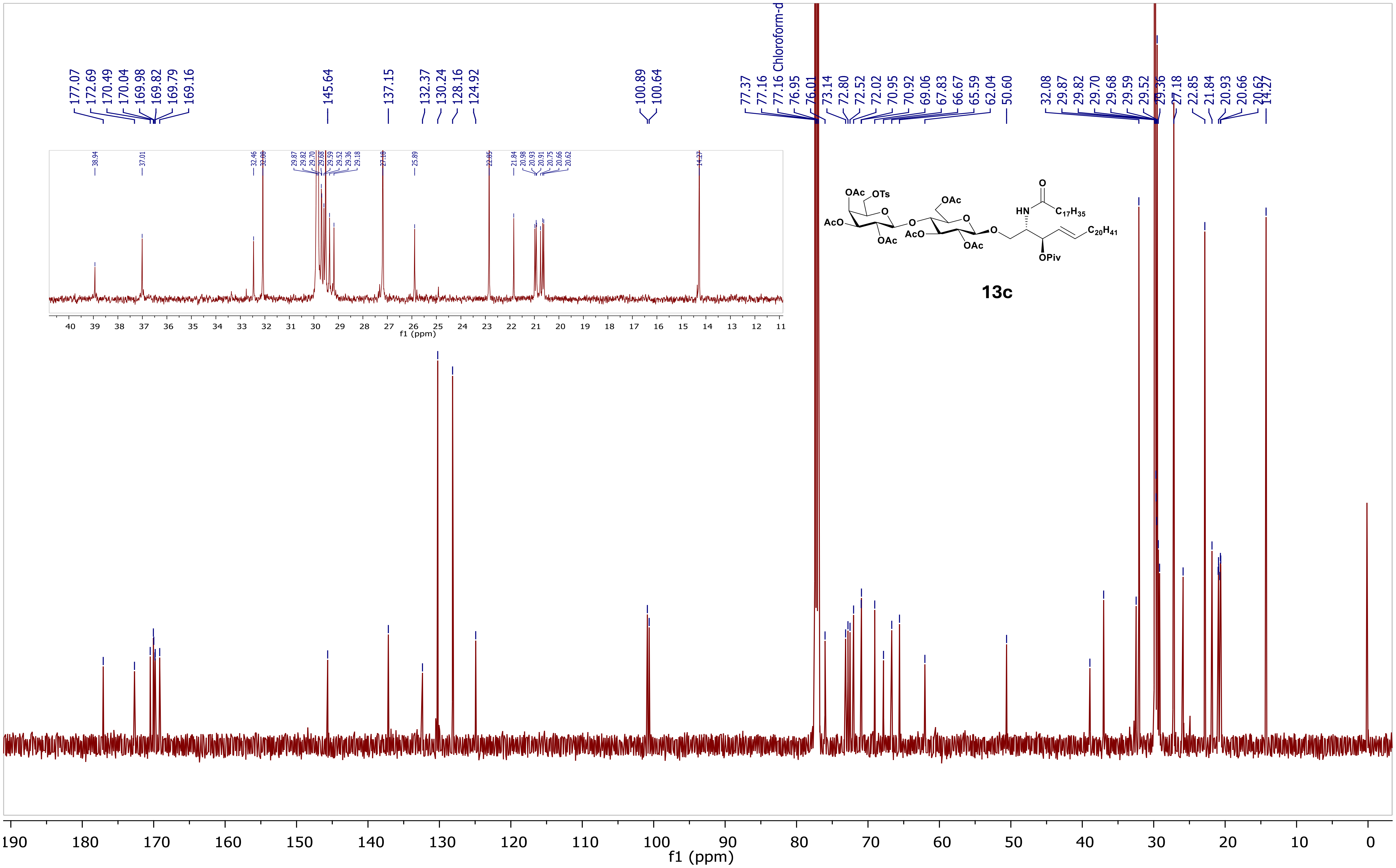

Figure 42. ^{1}H-^{1}H COSY NMR (600 MHz, $CDCl_3$) of compound **13c**

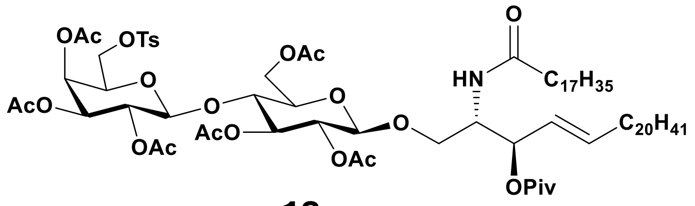

13c

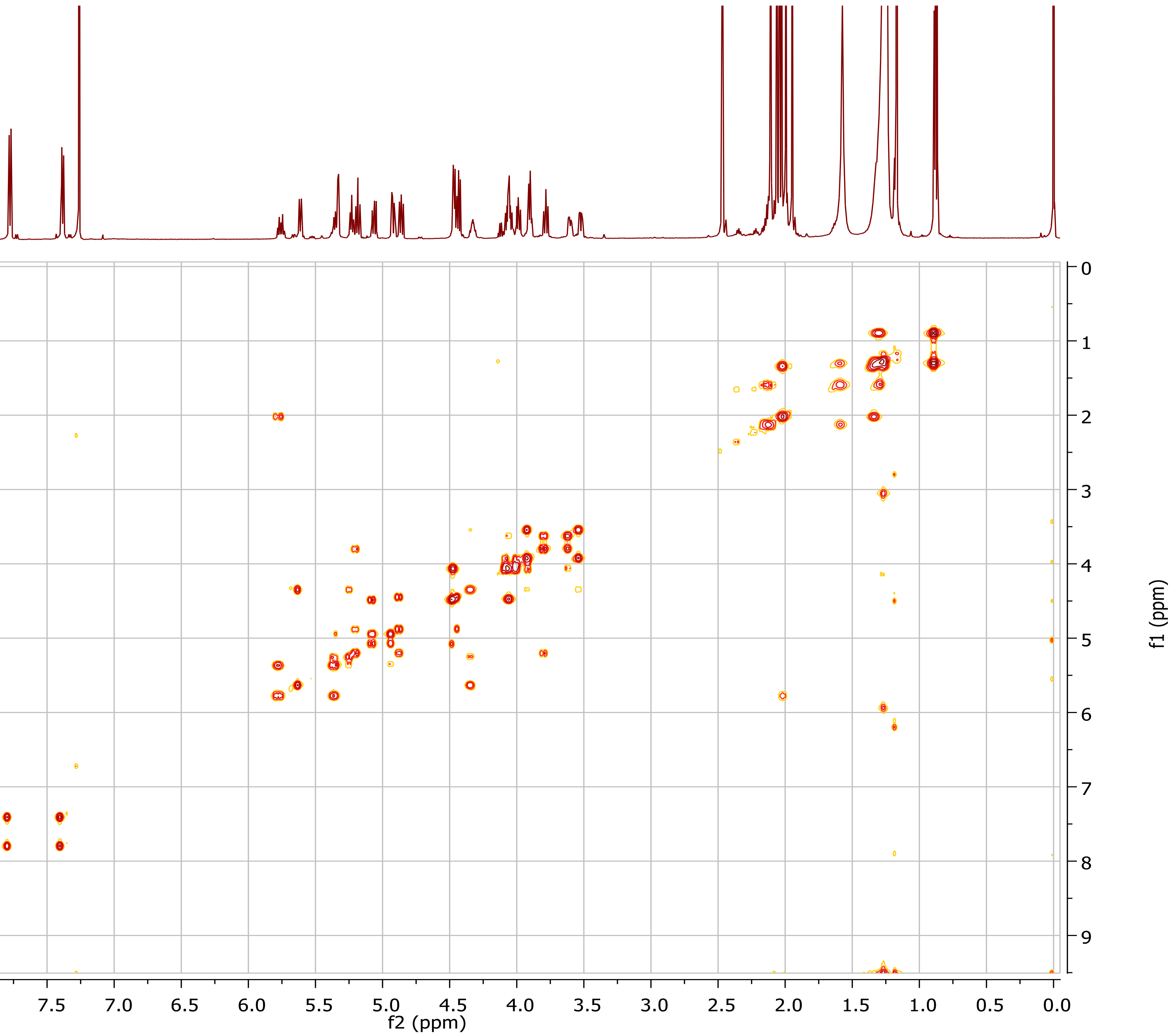

Figure S43. ^{1}H-^{13}C HSQC NMR (600/151 MHz, $CDCl_3$) of compound **13c**

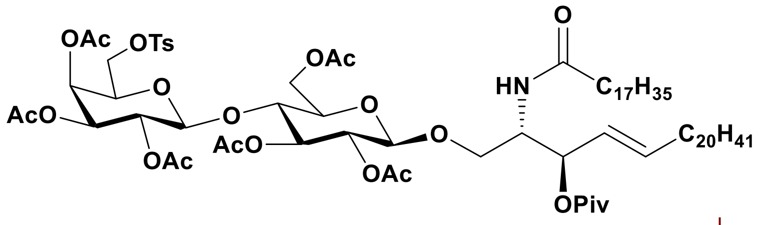

13c

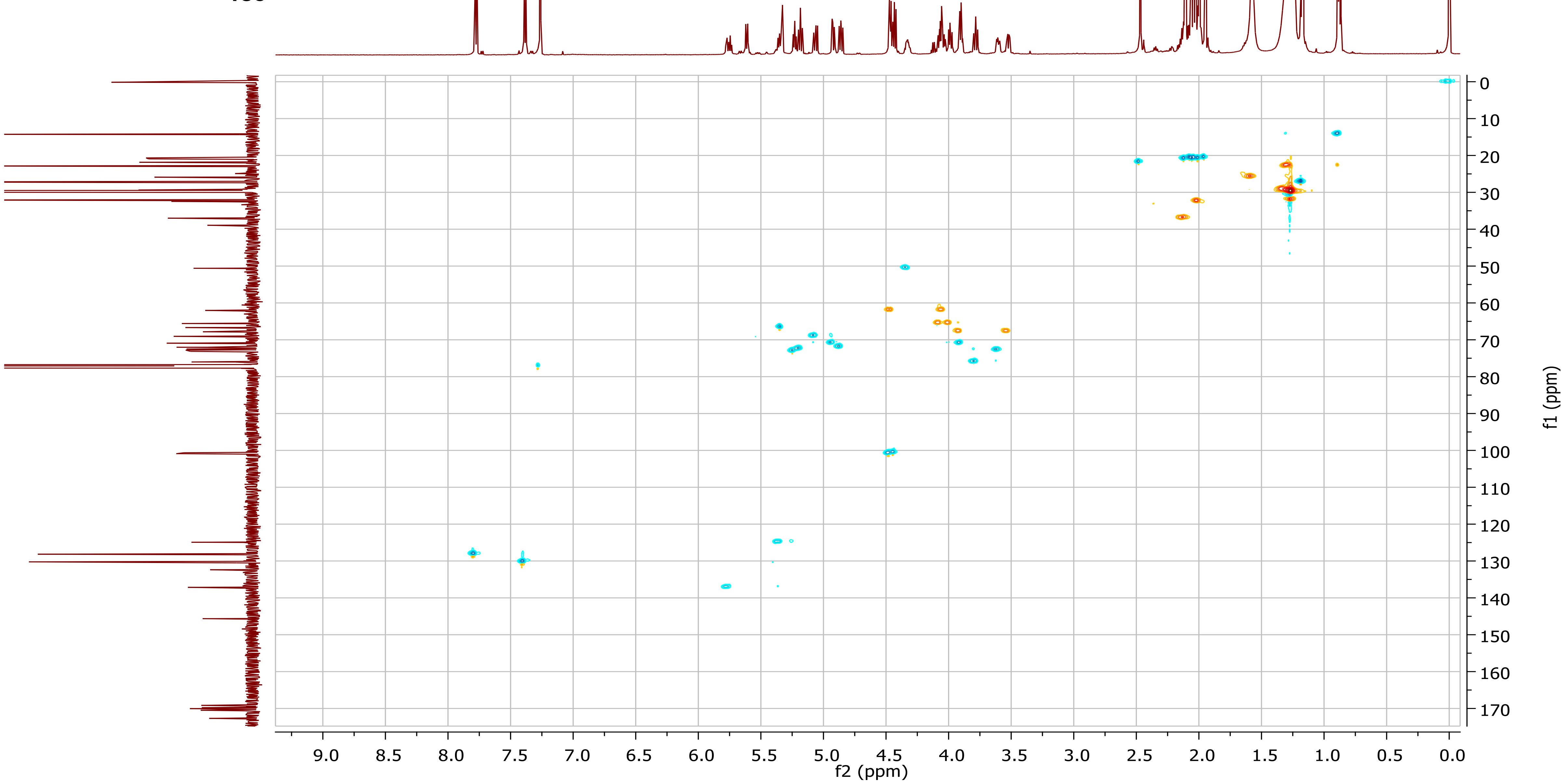

Figure S44. HR ESI-TOF-MS of compound **13c**

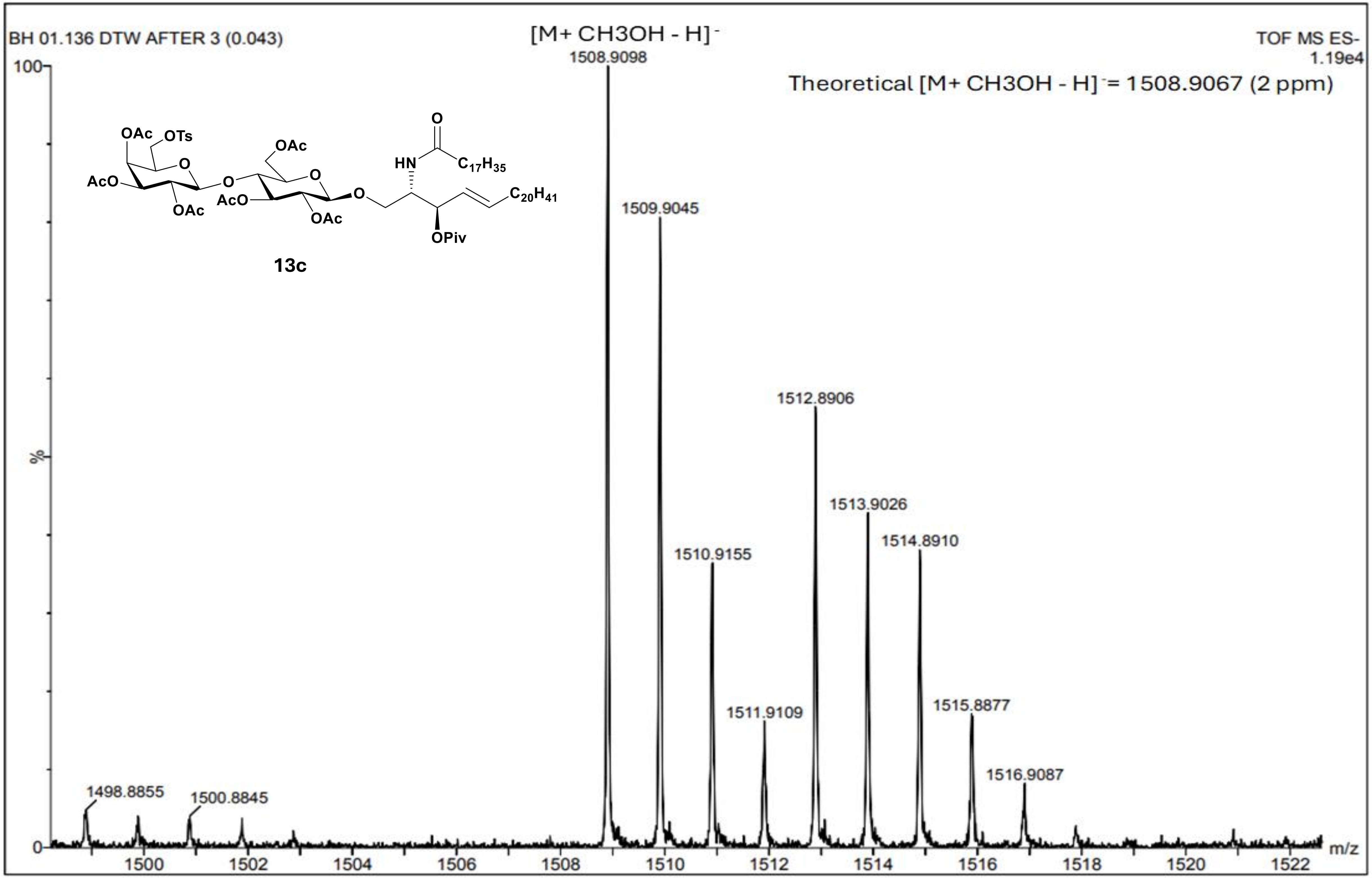

Figure S45. ^{1}H NMR of compound **13d** (600 MHz, $CDCl_3$)

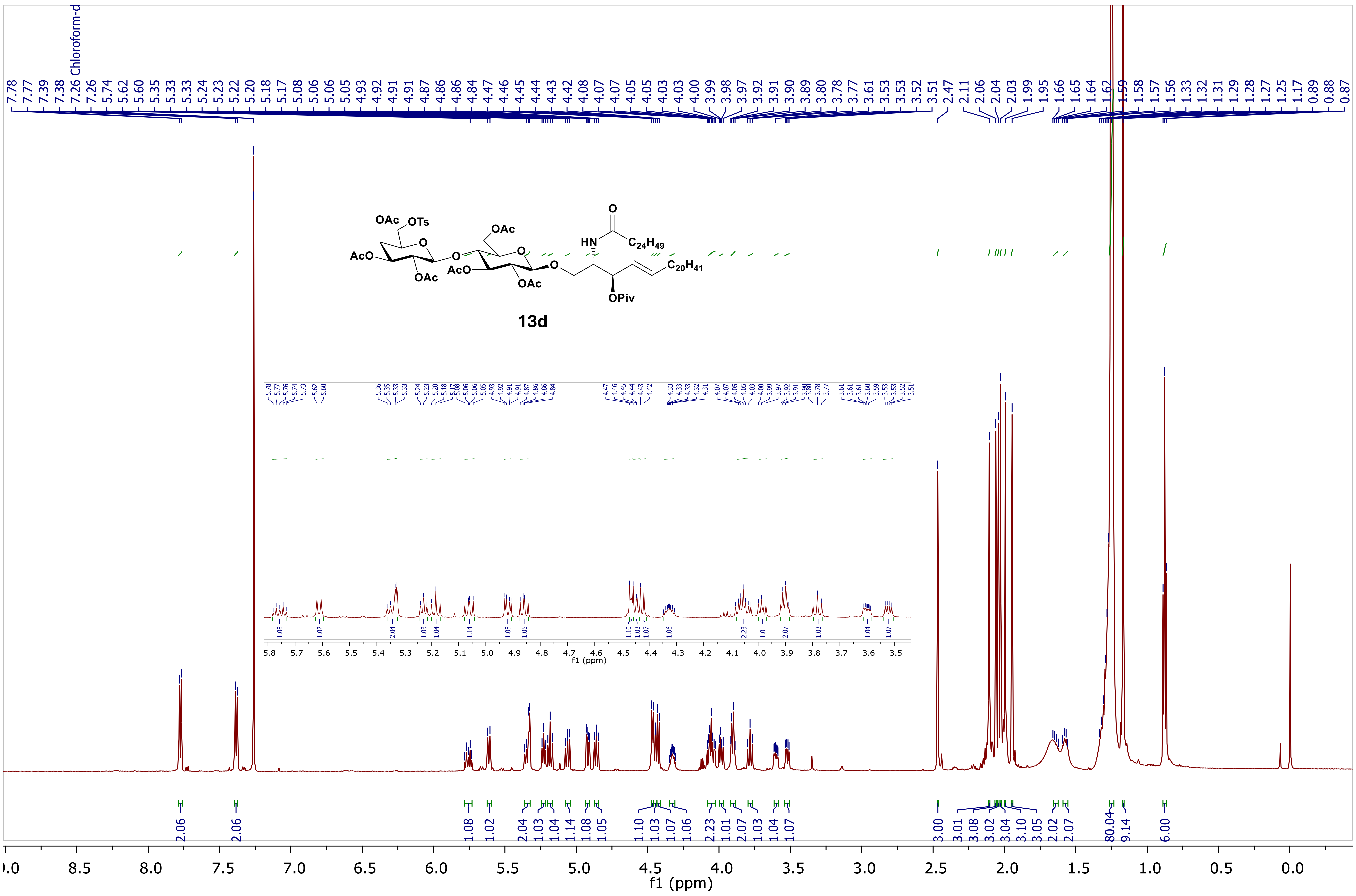

Figure S46. ^{13}C NMR of compound **13d** (151 MHz, $CDCl_3$)

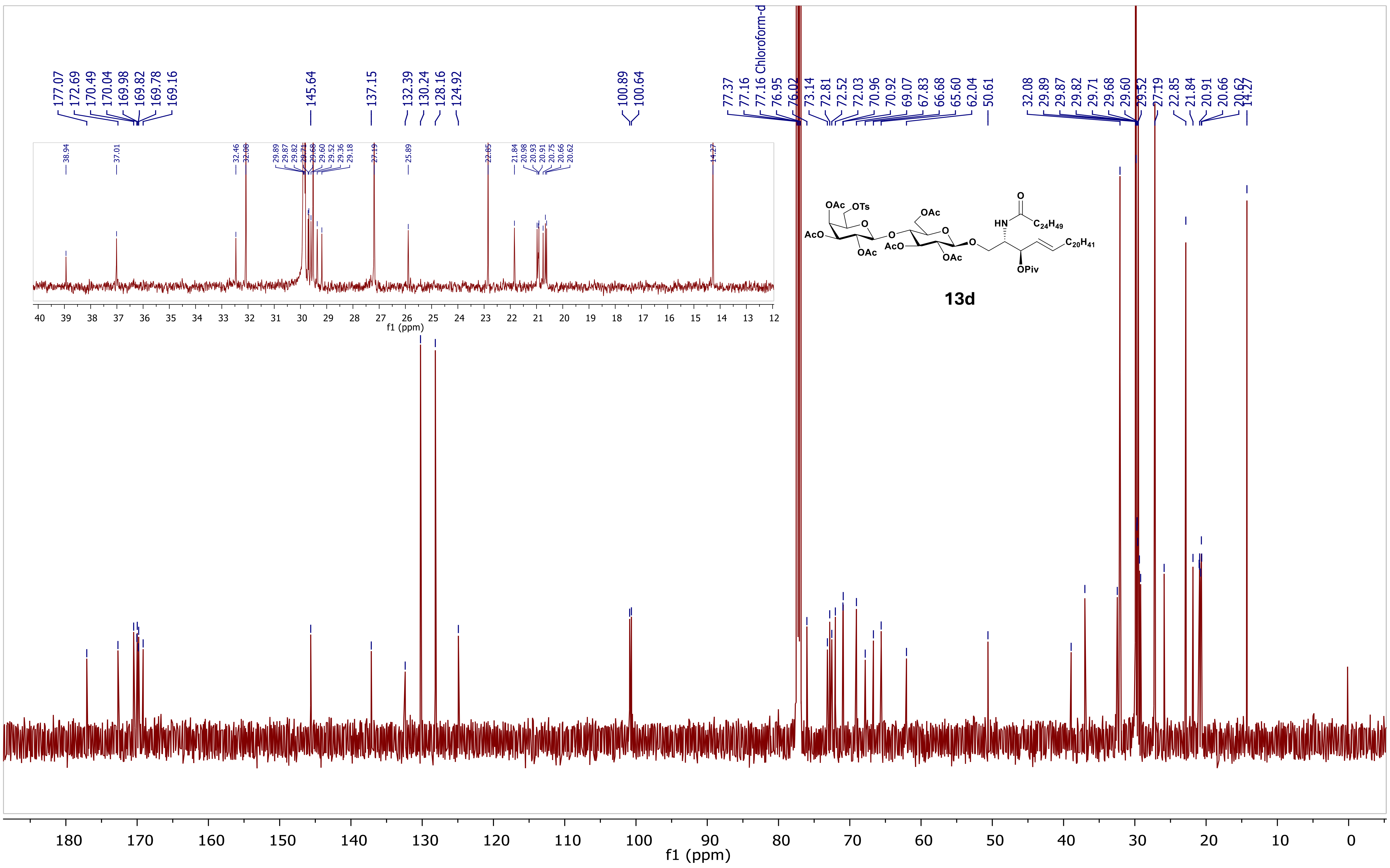

Figure S47. ^1H-^1H COSY NMR (600 MHz, $CDCl_3$) of compound **13d**

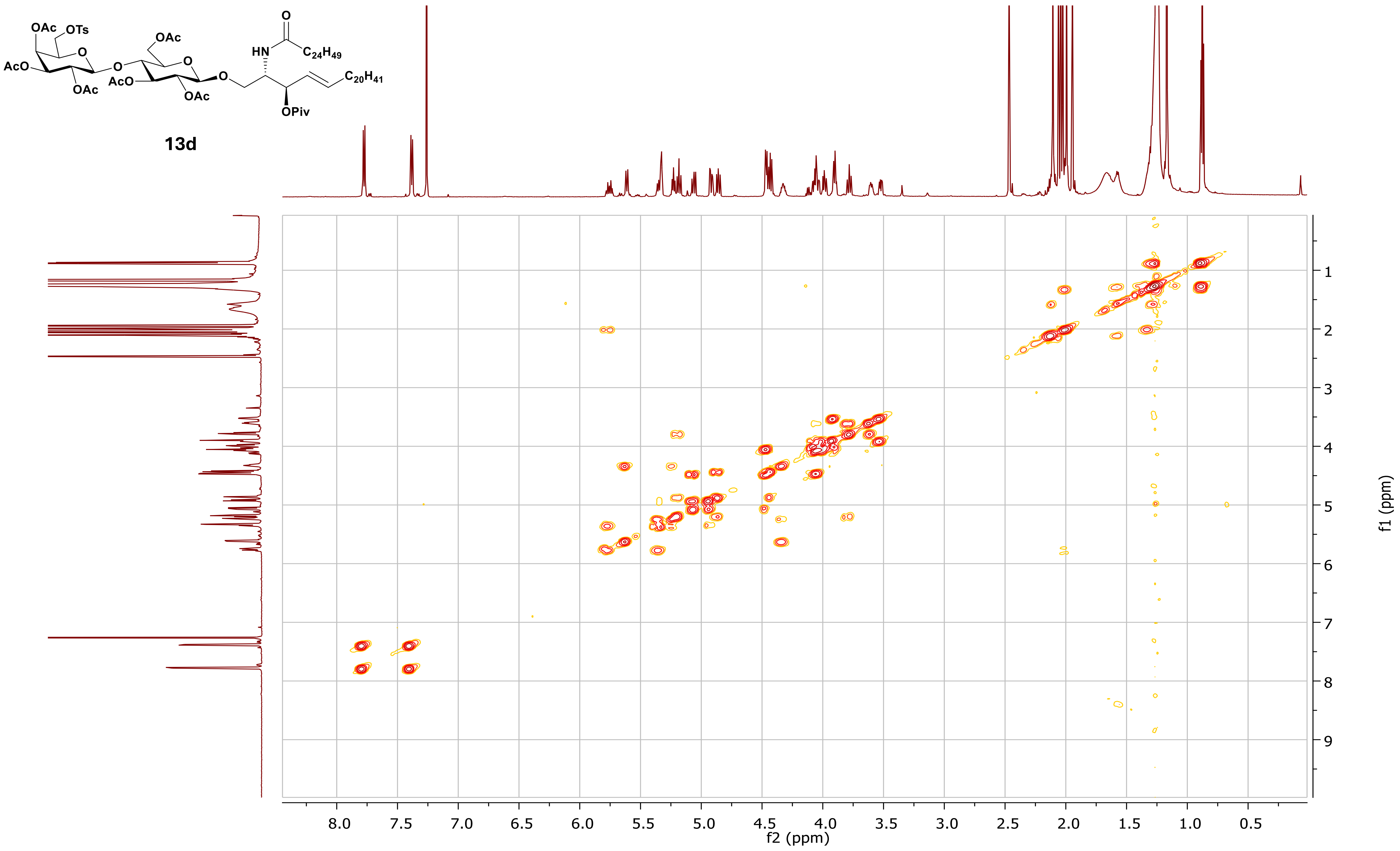

Figure S48. ^{1}H-^{13}C HSQC NMR (600/151 MHz, $CDCl_3$) of compound **13d**

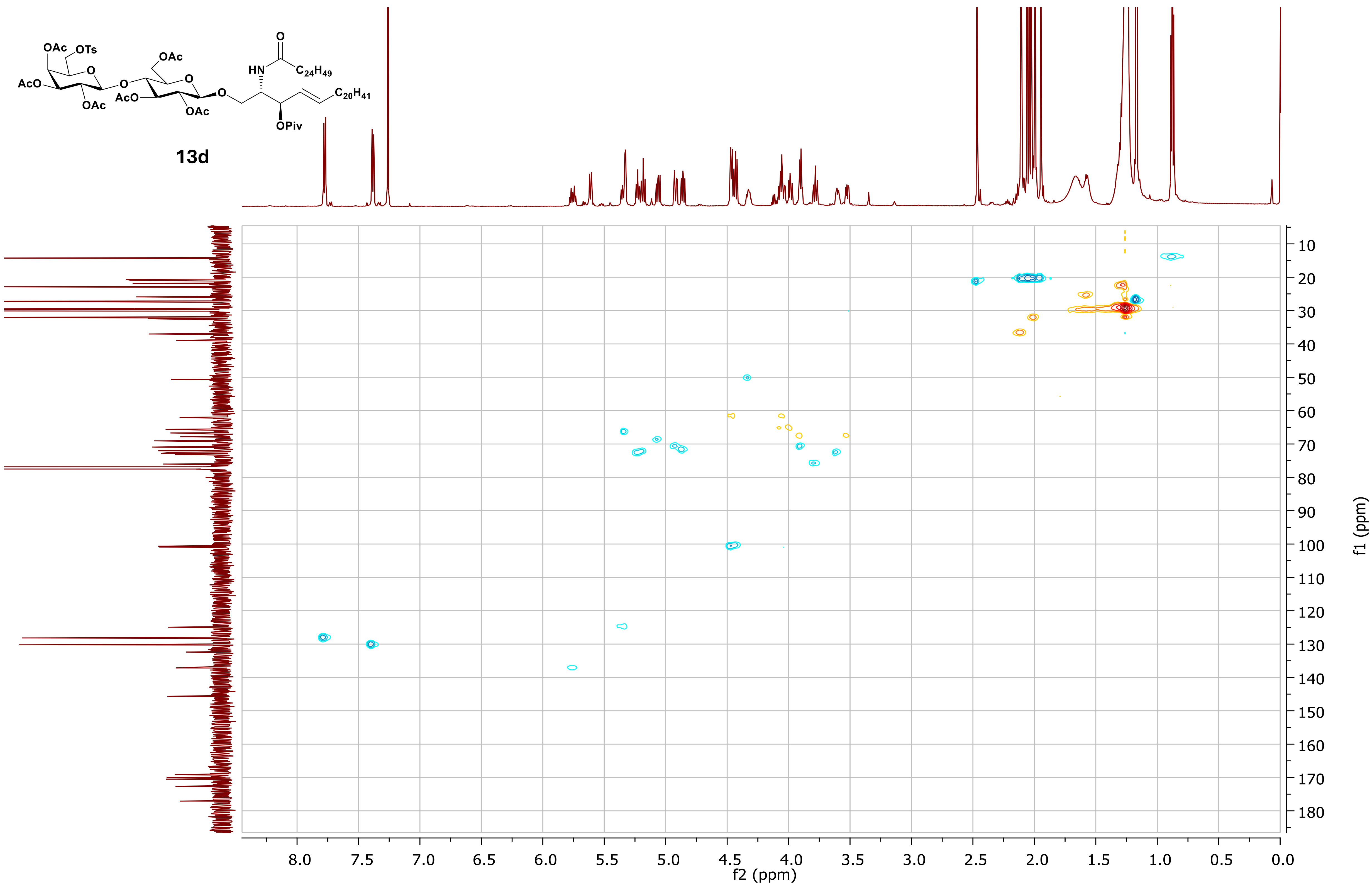

Figure S49. HR ESI-TOF-MS of compound **13d**

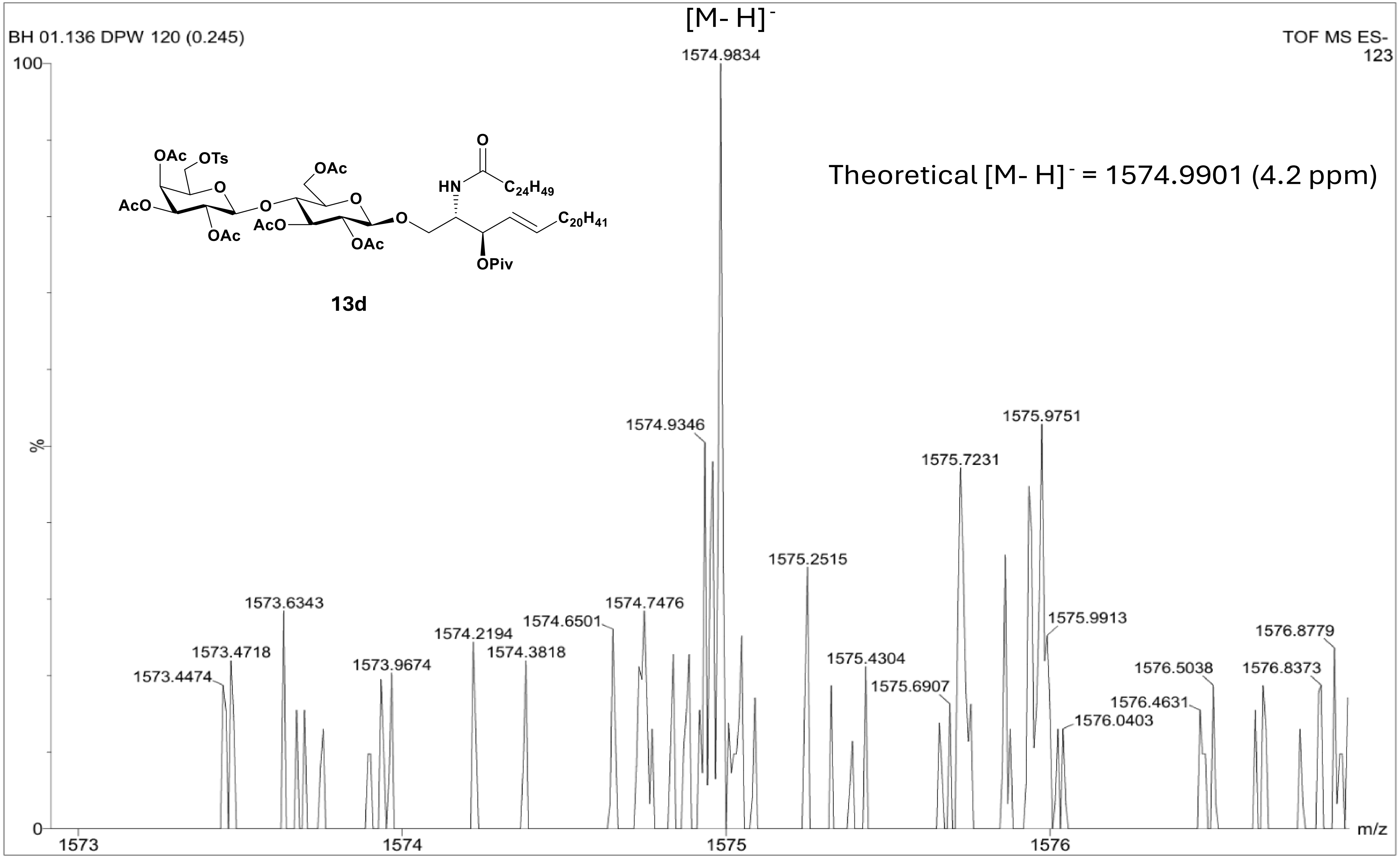

Figure S50. ^{1}H NMR of compound **14a** (600 MHz, $CDCl_3$)

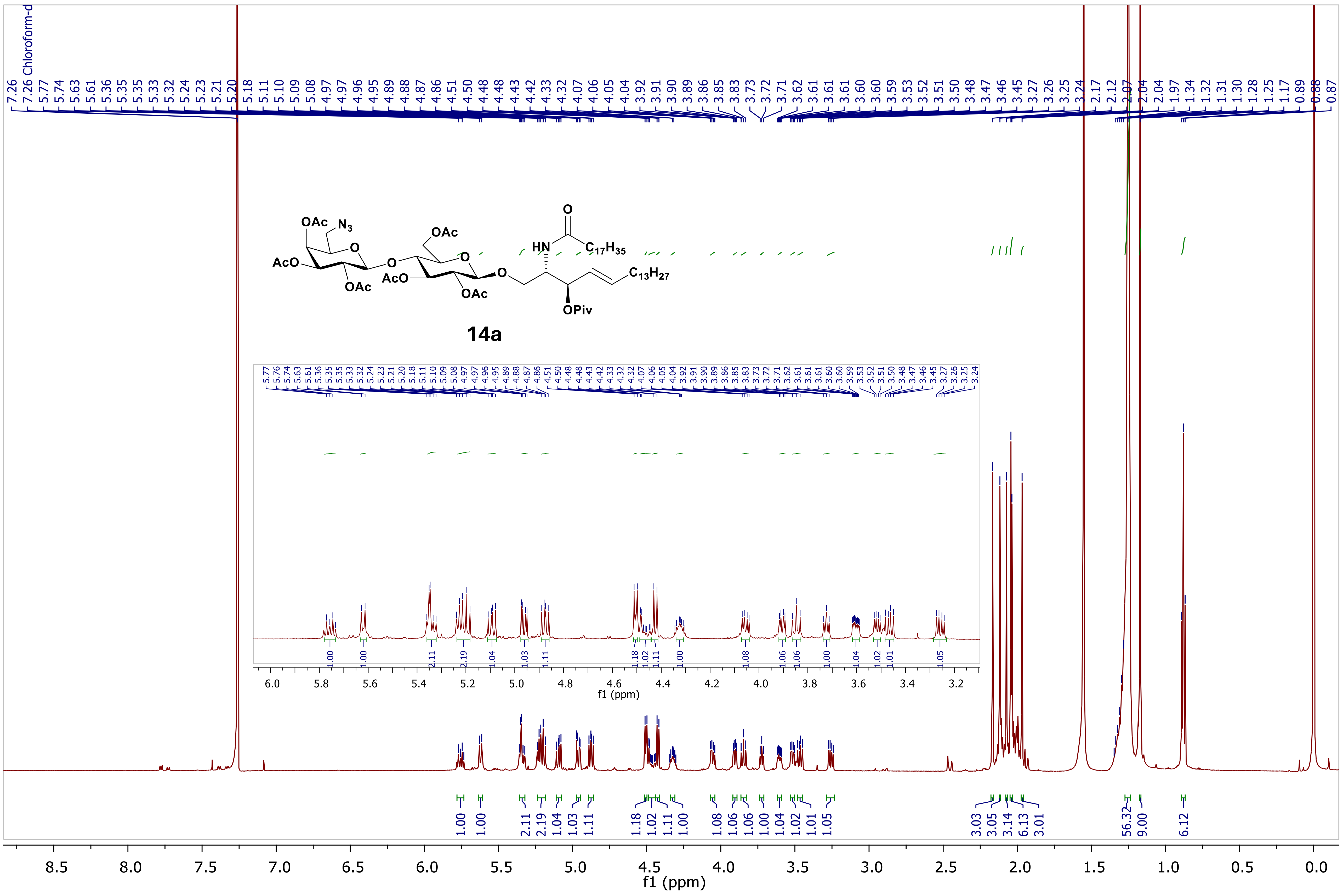

Figure S51. ^{13}C NMR of compound **14a** (151 MHz, $CDCl_3$)

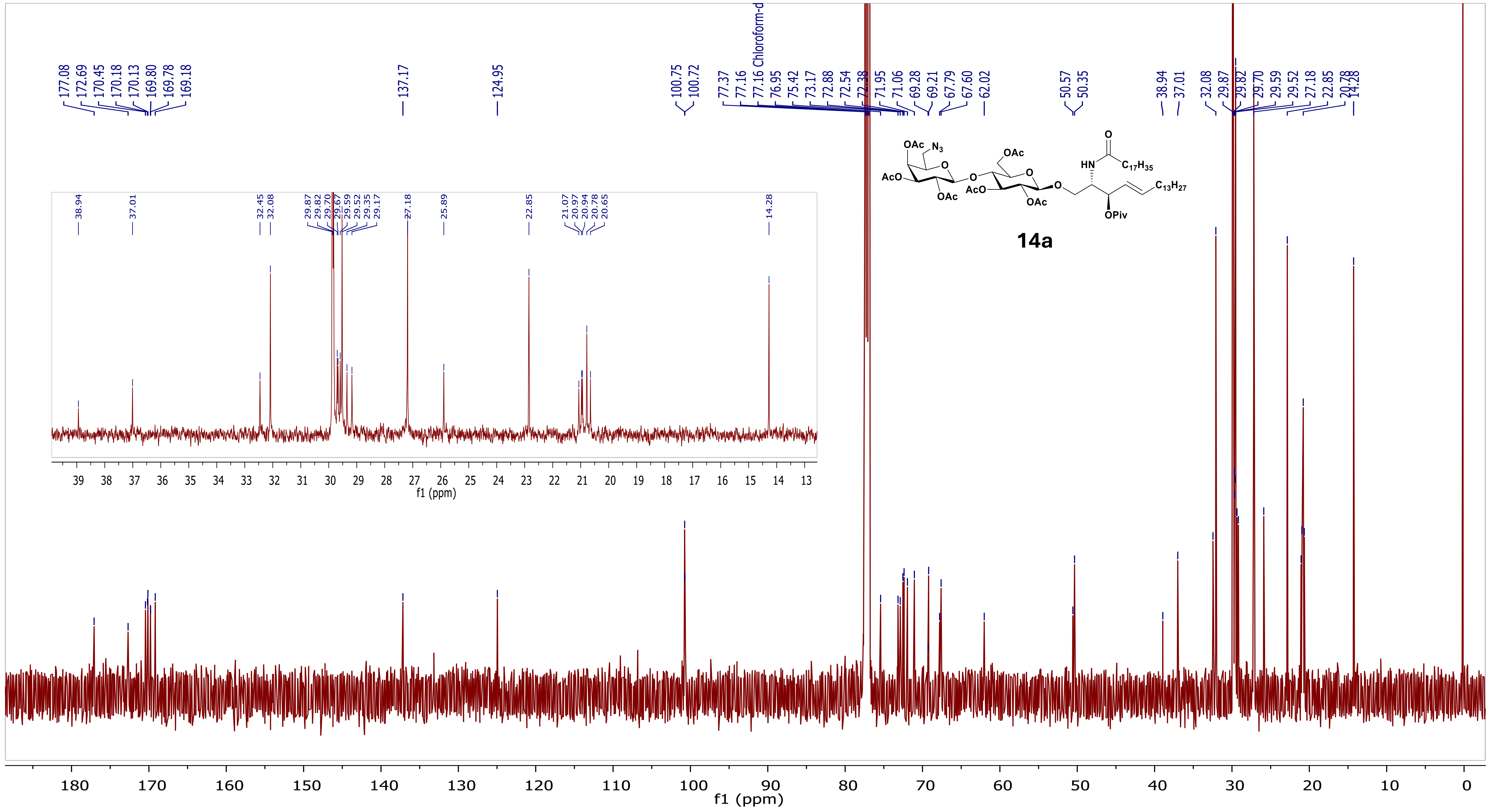

Figure 52. ^1H-^1H COSY NMR (600 MHz, $CDCl_3$) of compound **14a**

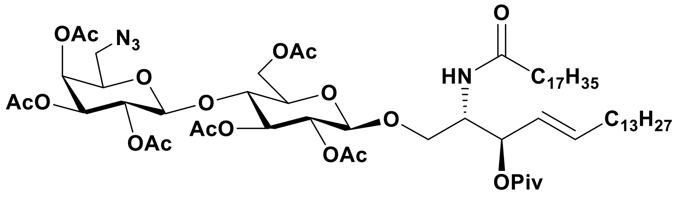

14a

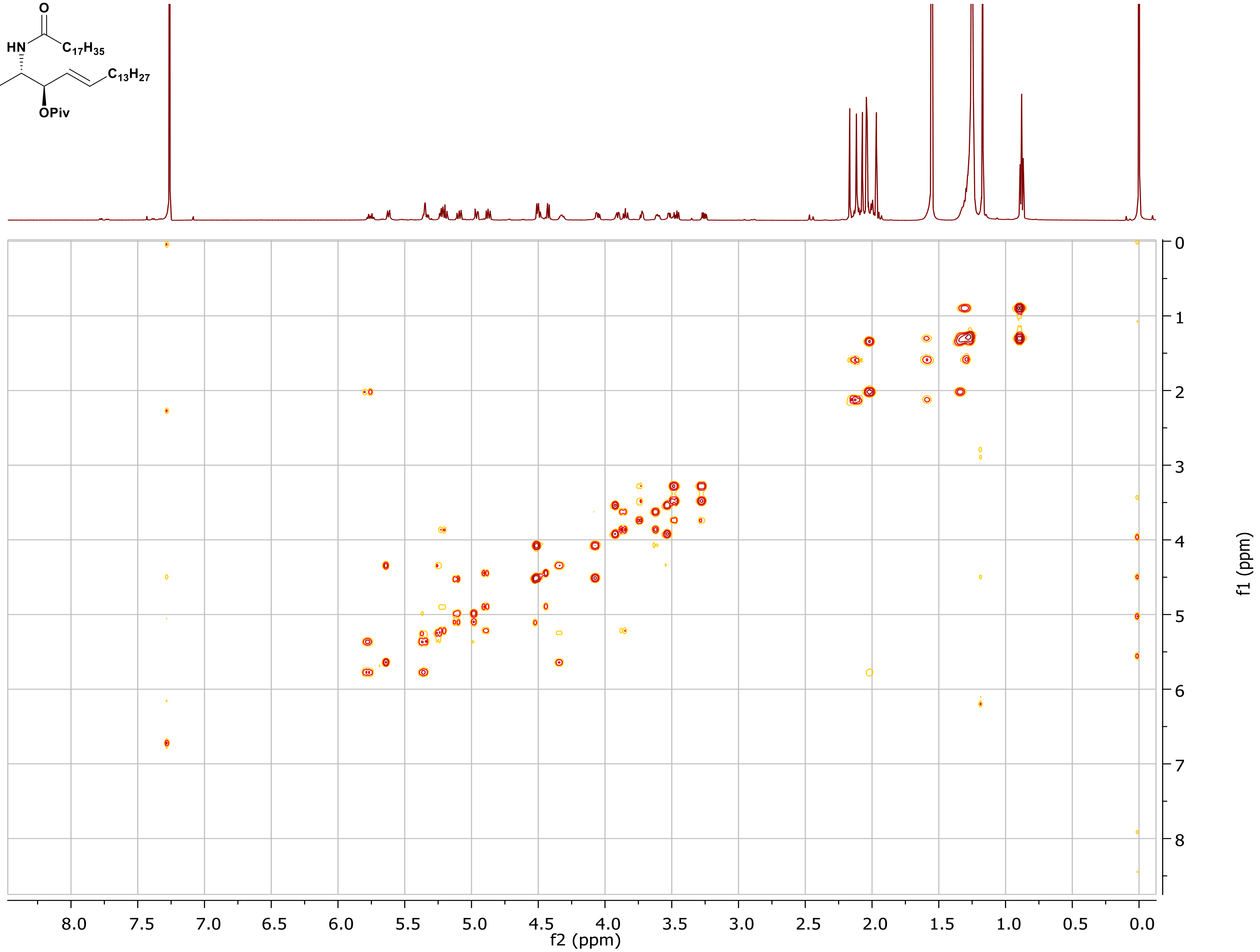

Figure S53. ^{1}H-^{13}C HSQC NMR (600/151 MHz, $CDCl_3$) of compound **14a**

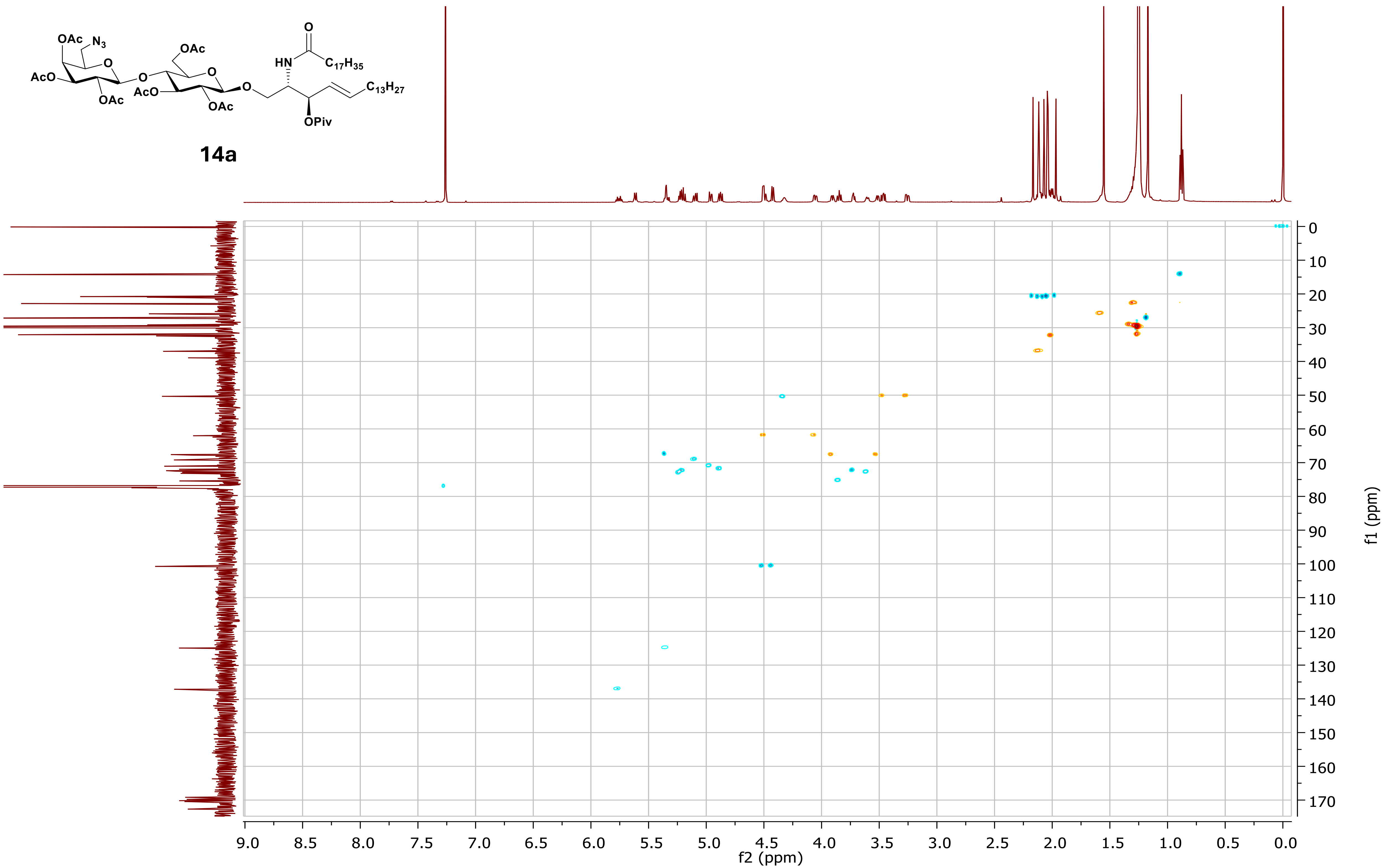

Figure S54. HR ESI-TOF-MS of compound **14a**

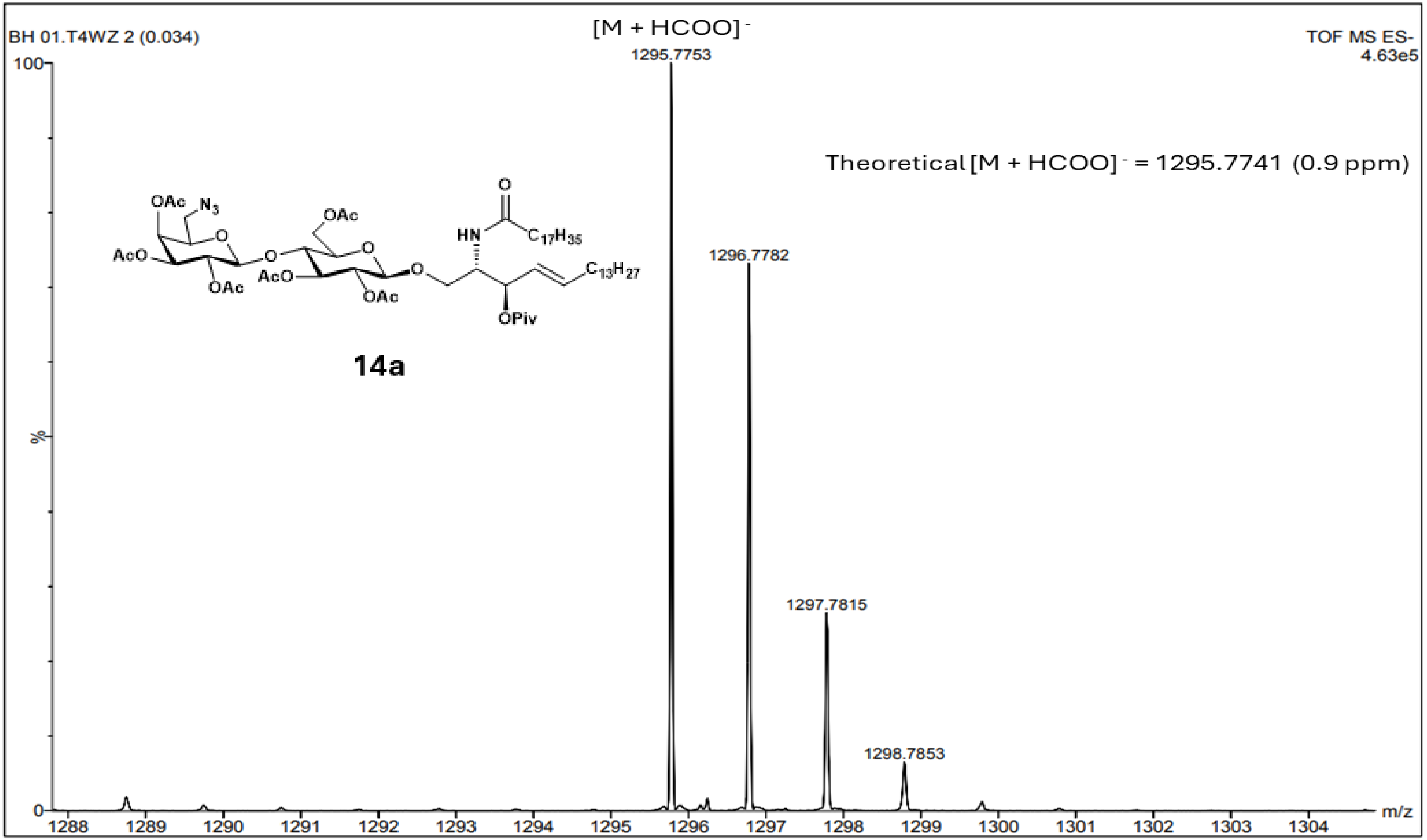

Figure S55. ^{1}H NMR of compound **14b** (600 MHz, $CDCl_3$)

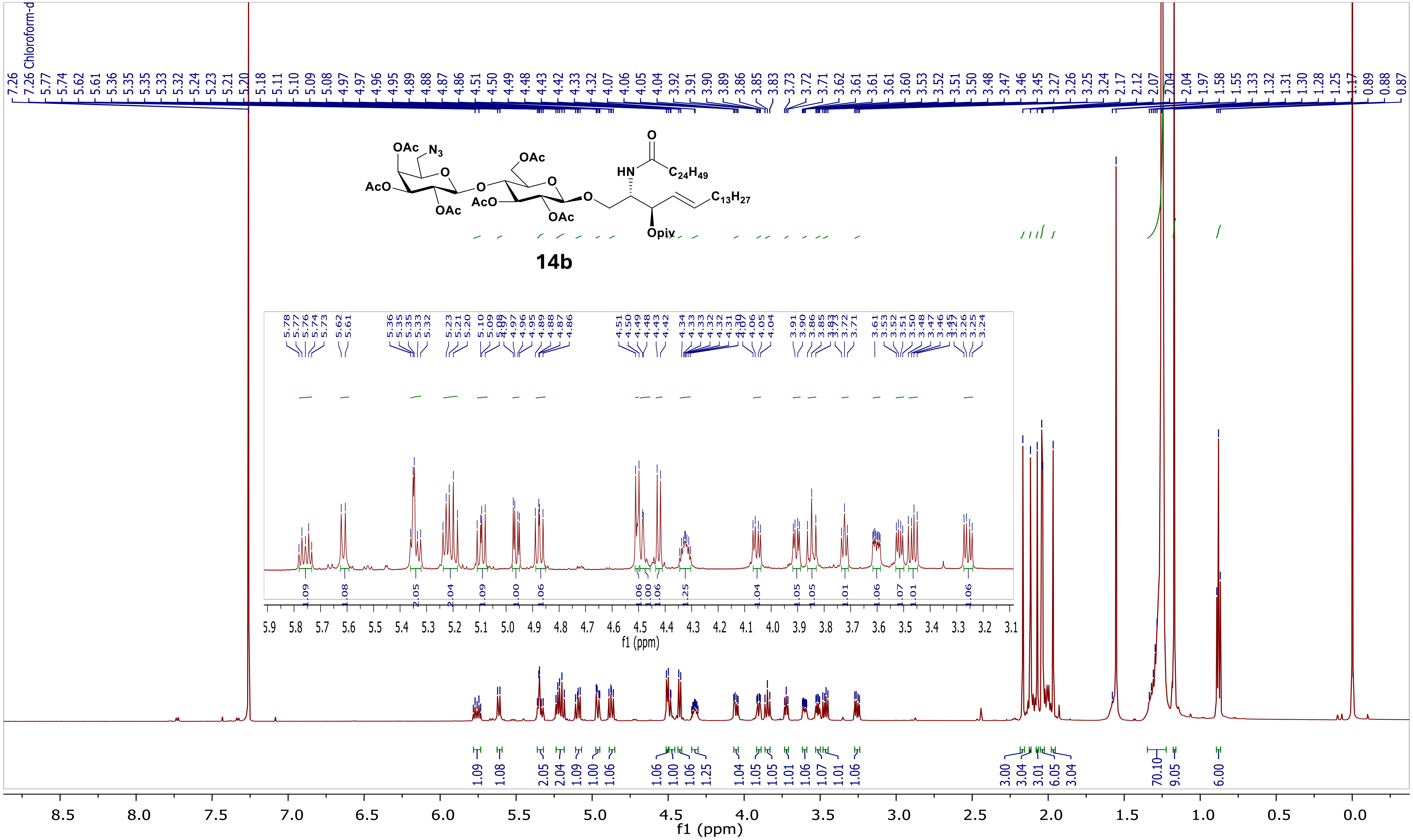

Figure S56. ^{13}C NMR of Compound **14b** (151 MHz, $CDCl_3$)

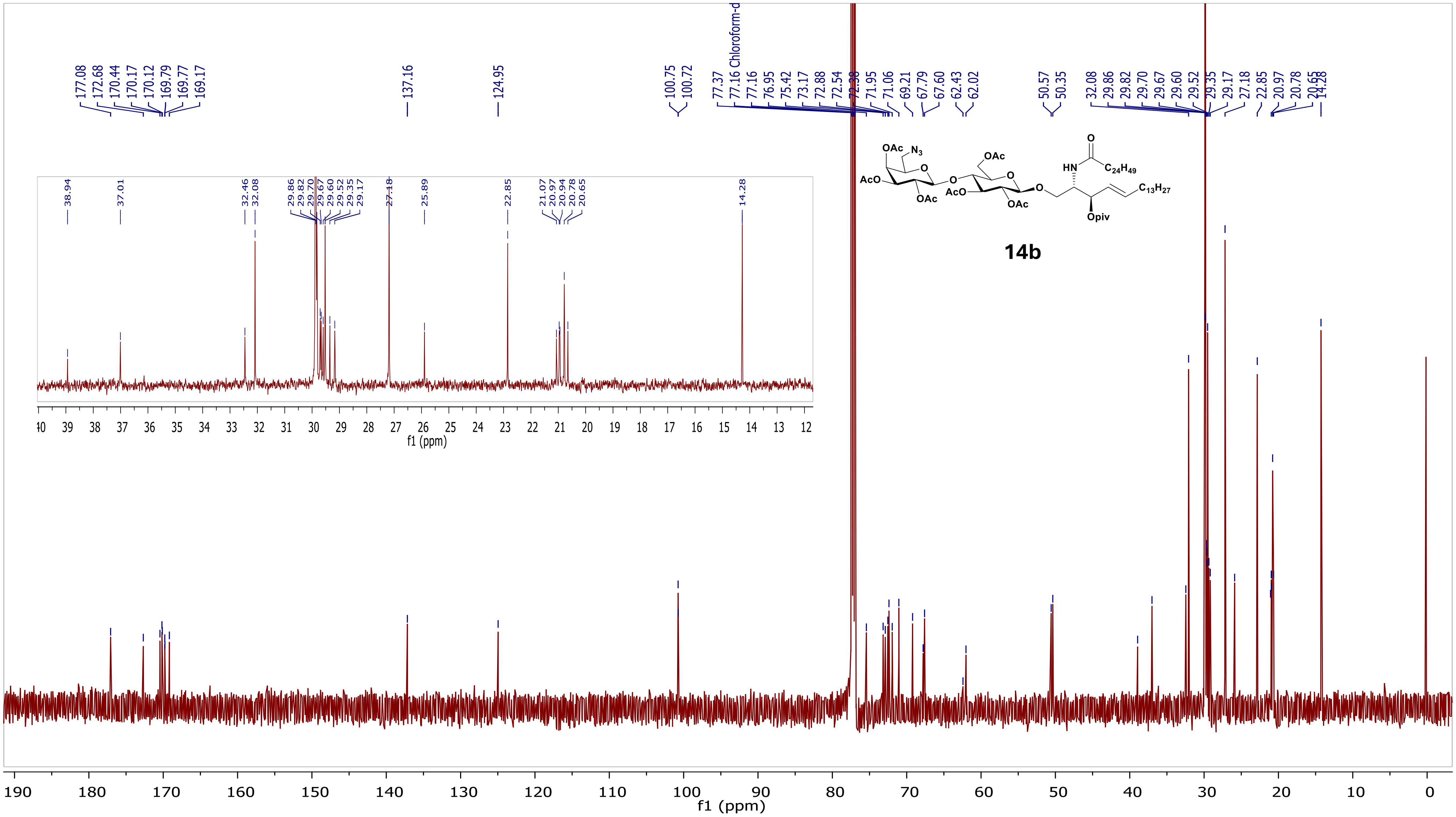

Figure S57. ^{1}H-^{1}H COSY NMR (600 MHz, $CDCl_3$) of compound **14b**

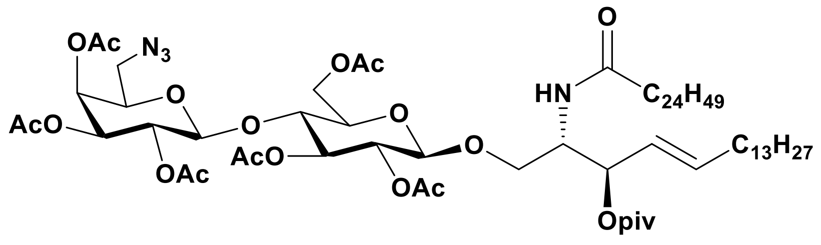

14b

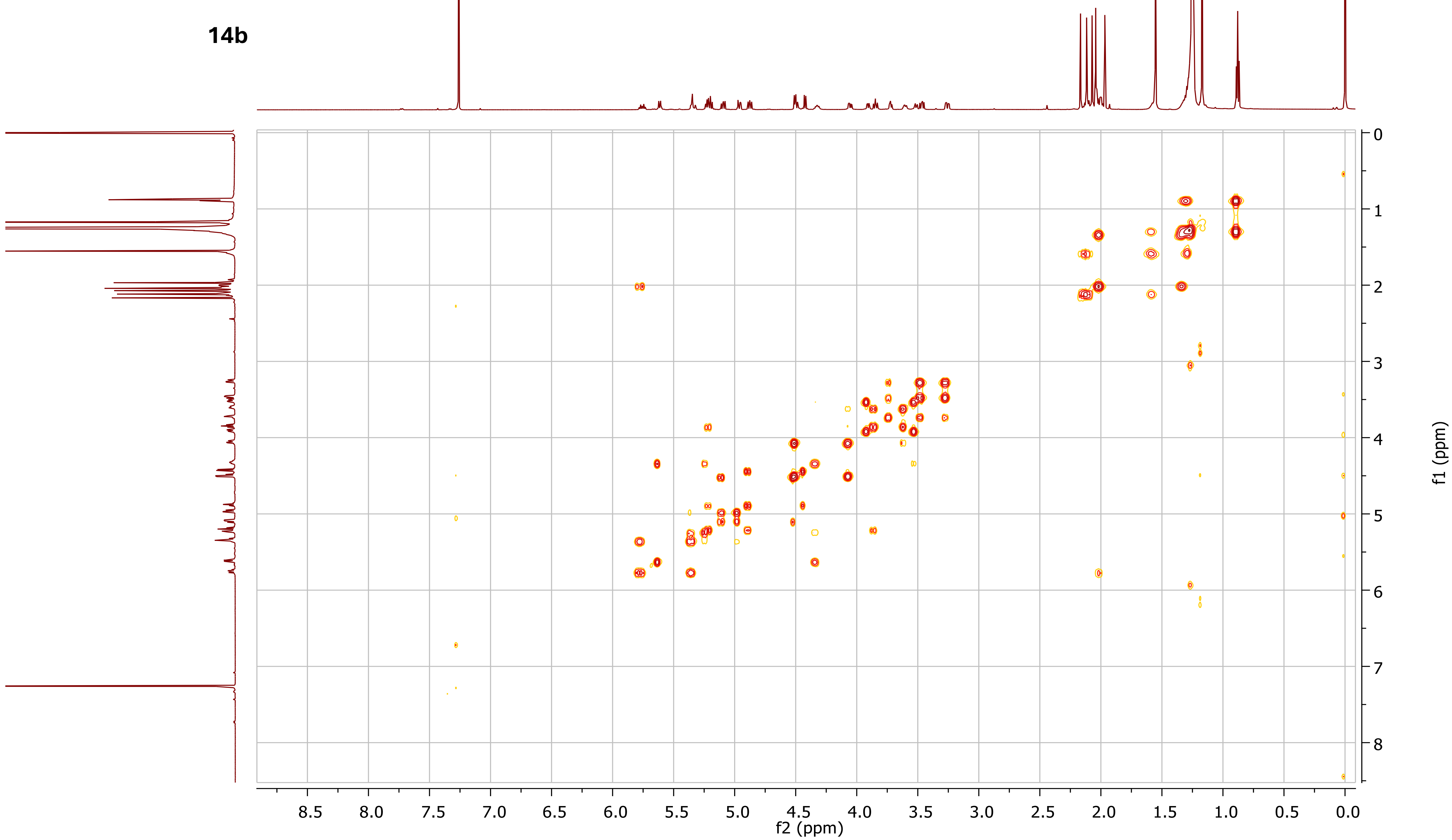

Figure S58. ^1H-^{13}C HSQC NMR (600/151 MHz, $CDCl_3$) of compound **14b**

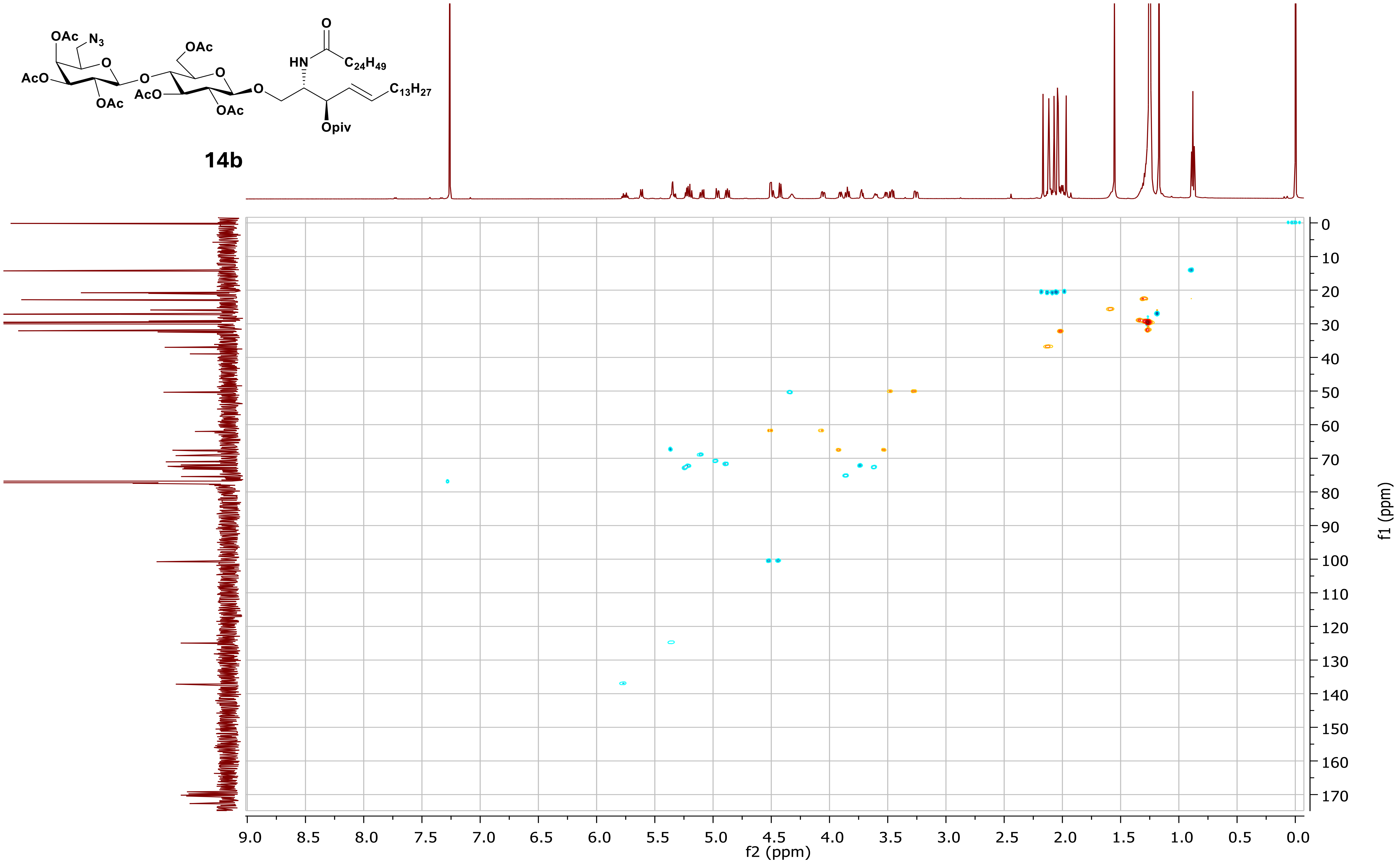

Figure S59. HR ESI-TOF-MS of compound **14b**

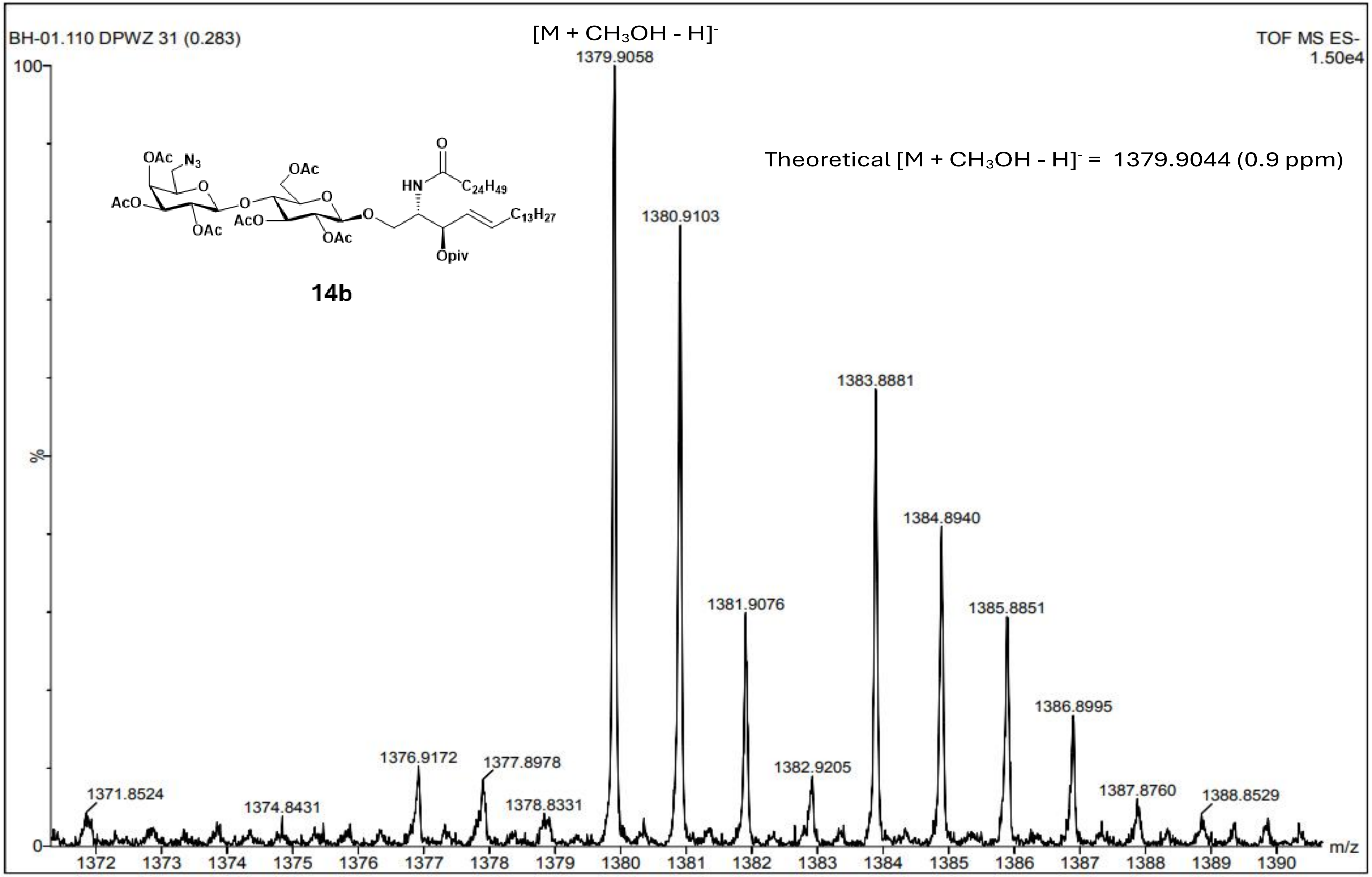

Figure S60. ^{1}H NMR of compound **14c** (600 MHz, $CDCl_3$)

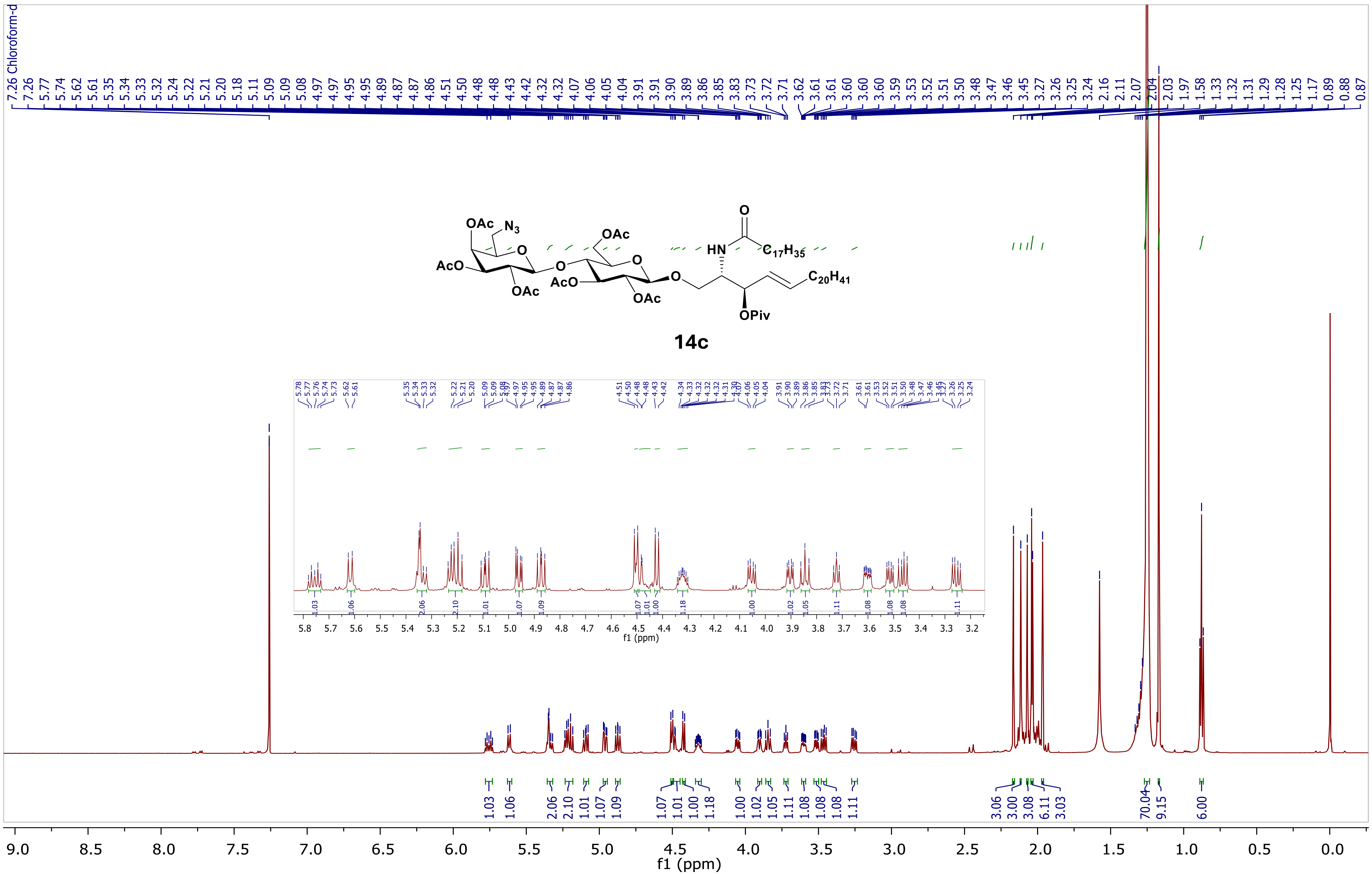

Figure S61. ^{13}C NMR of compound **14c** (151 MHz, $CDCl_3$)

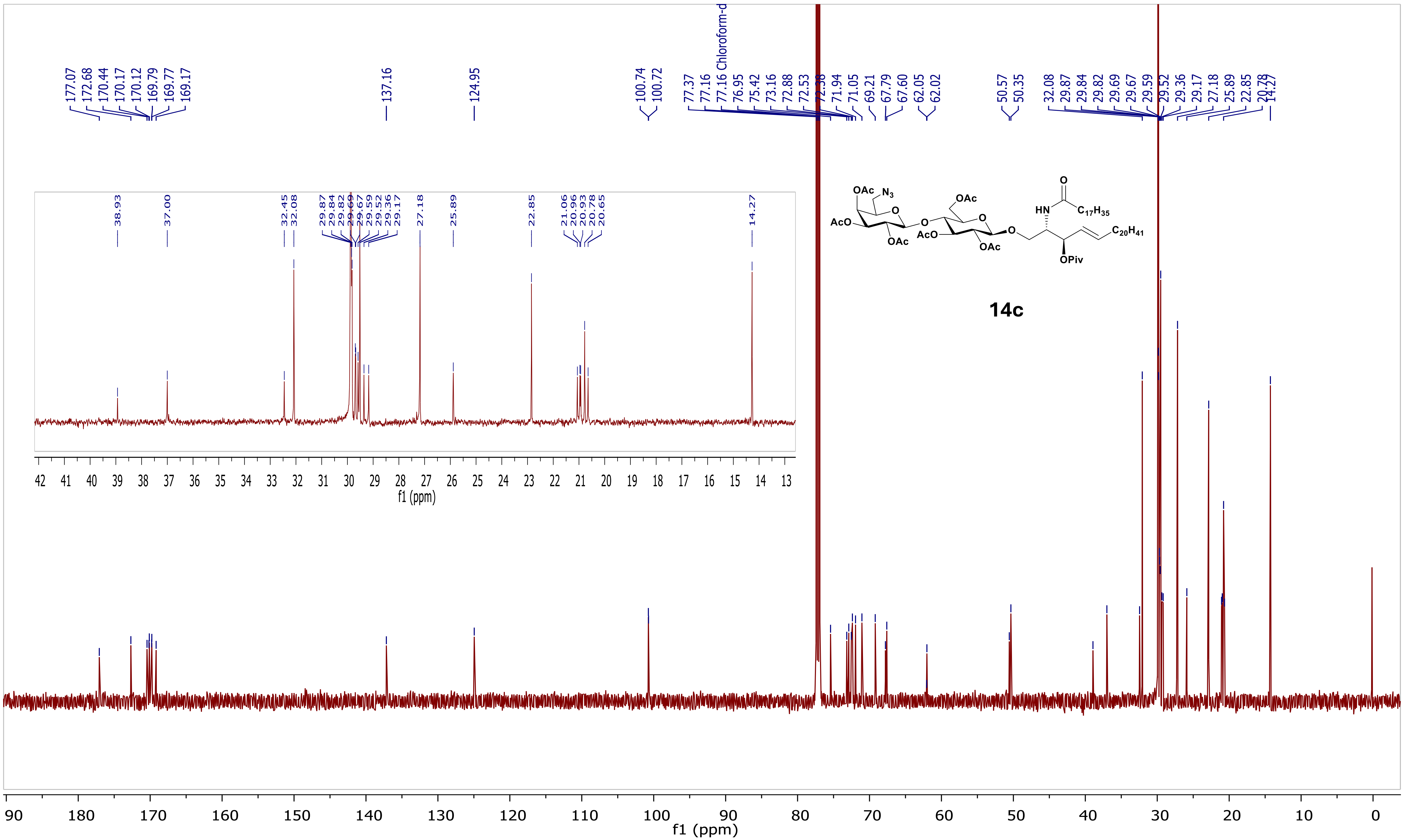

Figure S62. ^{1}H-^{1}H COSY NMR (600 MHz, $CDCl_3$) of compound **14c**

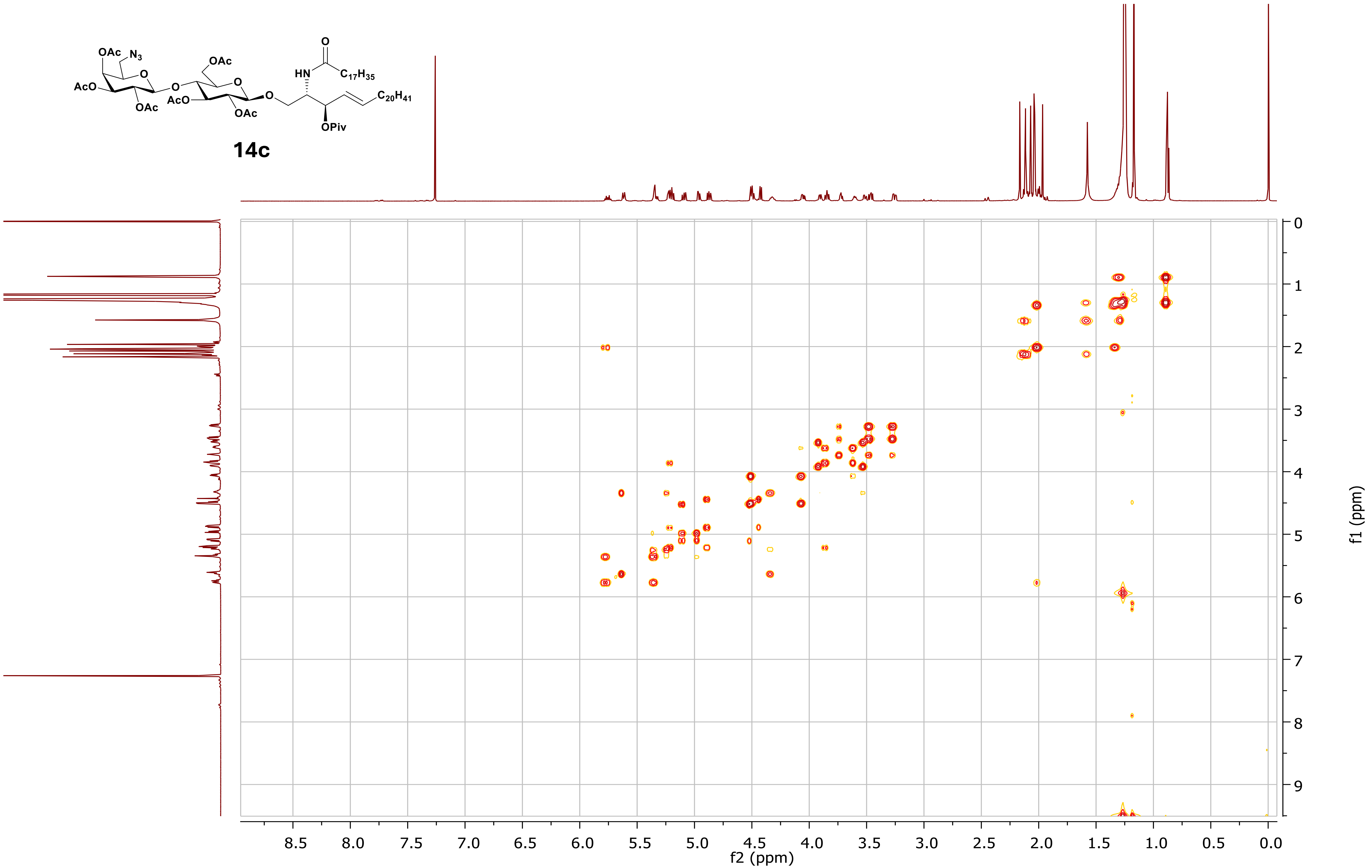

Figure S63. ^{1}H-^{13}C HSQC NMR (600/151 MHz, $CDCl_3$) of compound **14c**

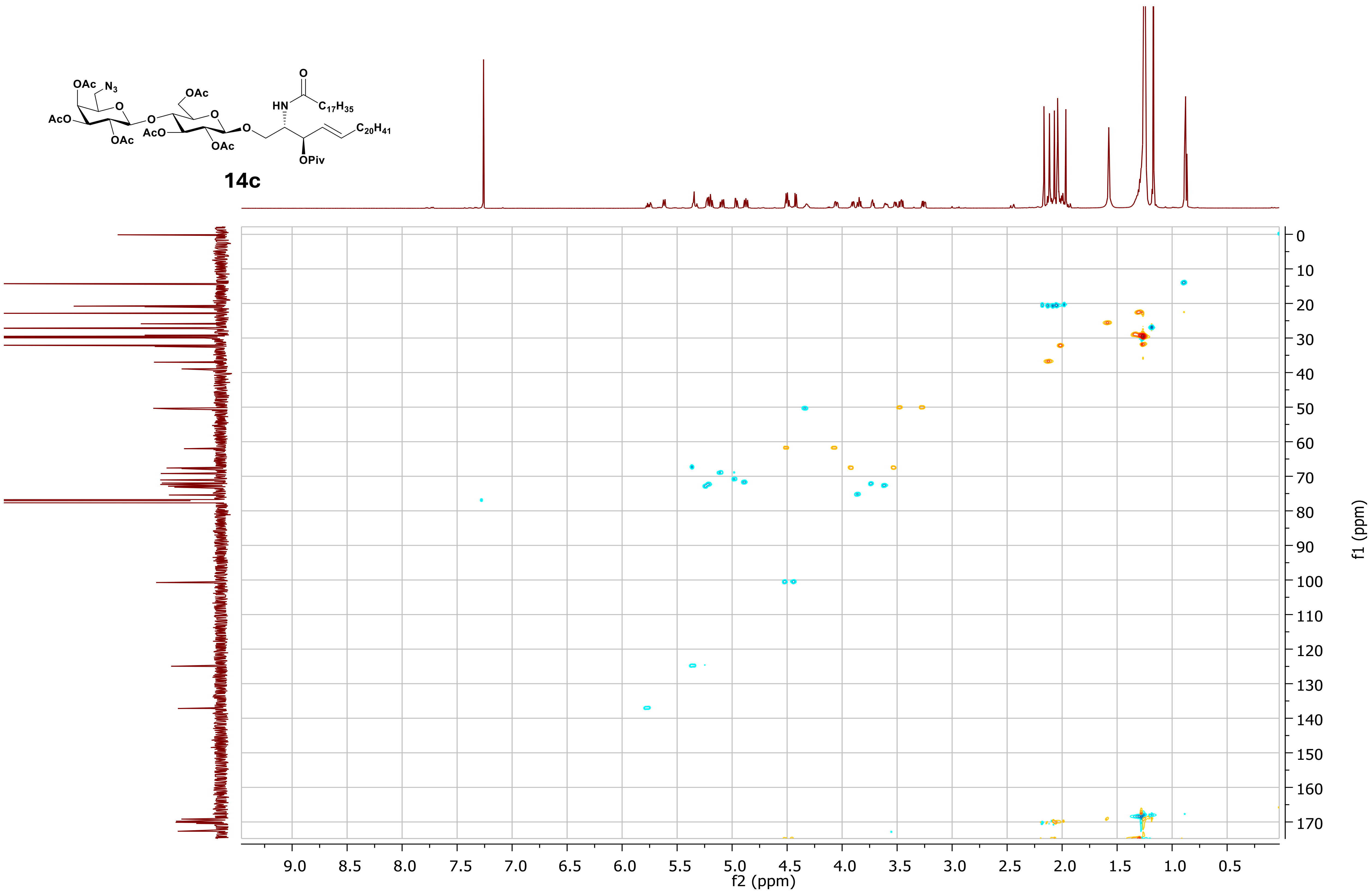

Figure S64. HR ESI-TOF-MS of compound **14c**

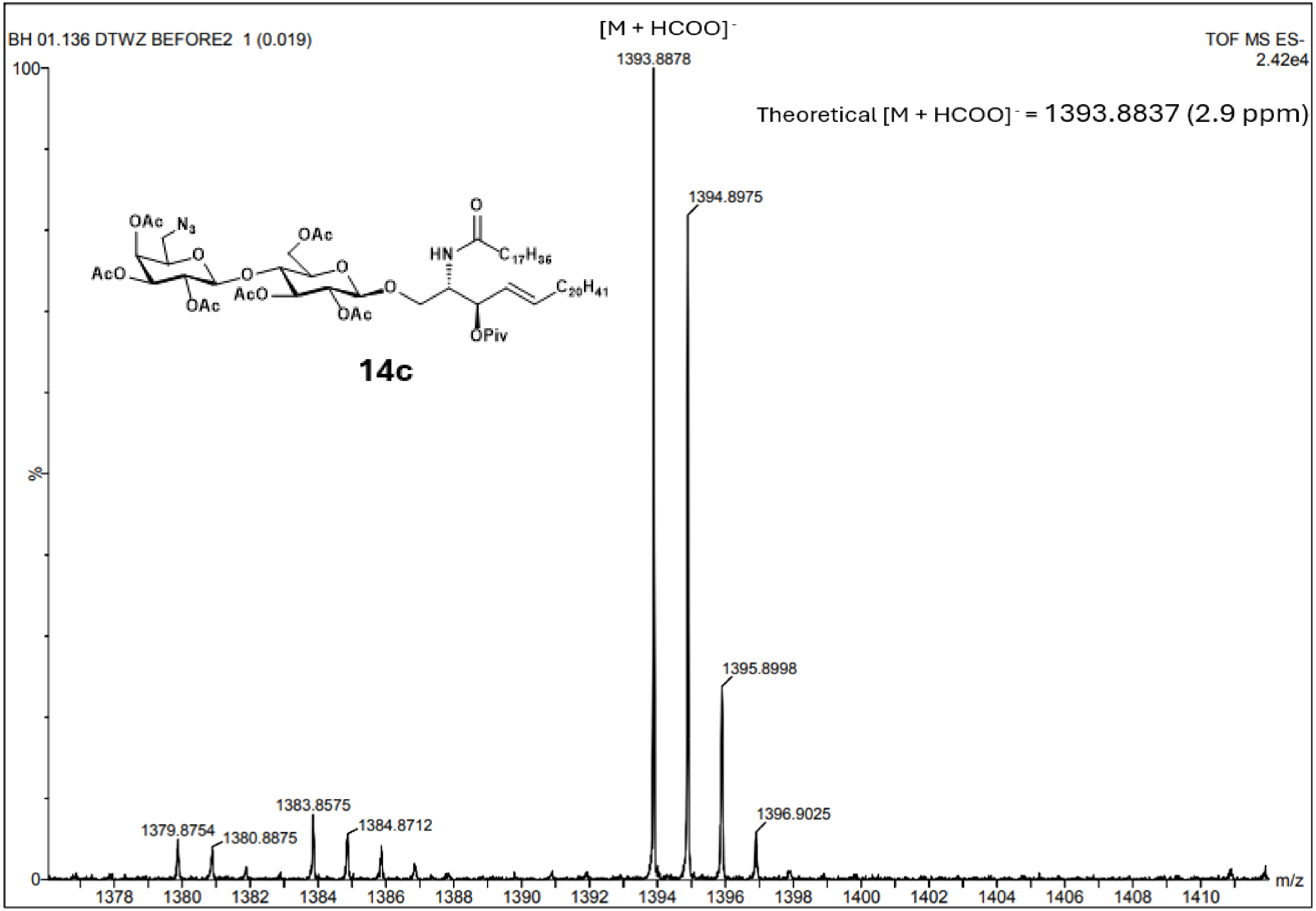

Figure S65. ^{1}H NMR of compound **14d** (600 MHz, $CDCl_3$)

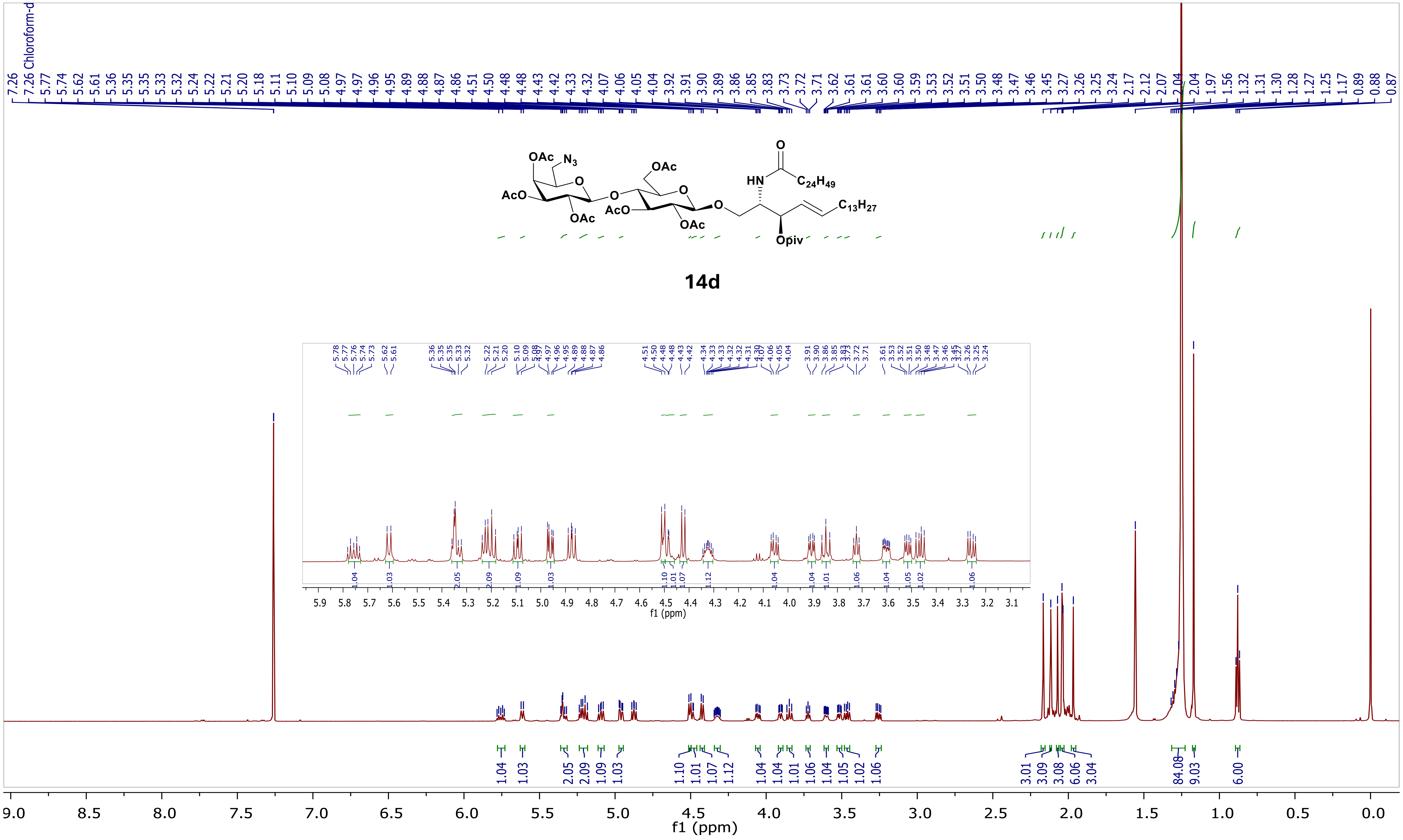

Figure S66. ^{13}C NMR of compound **14d** (151 MHz, $CDCl_3$)

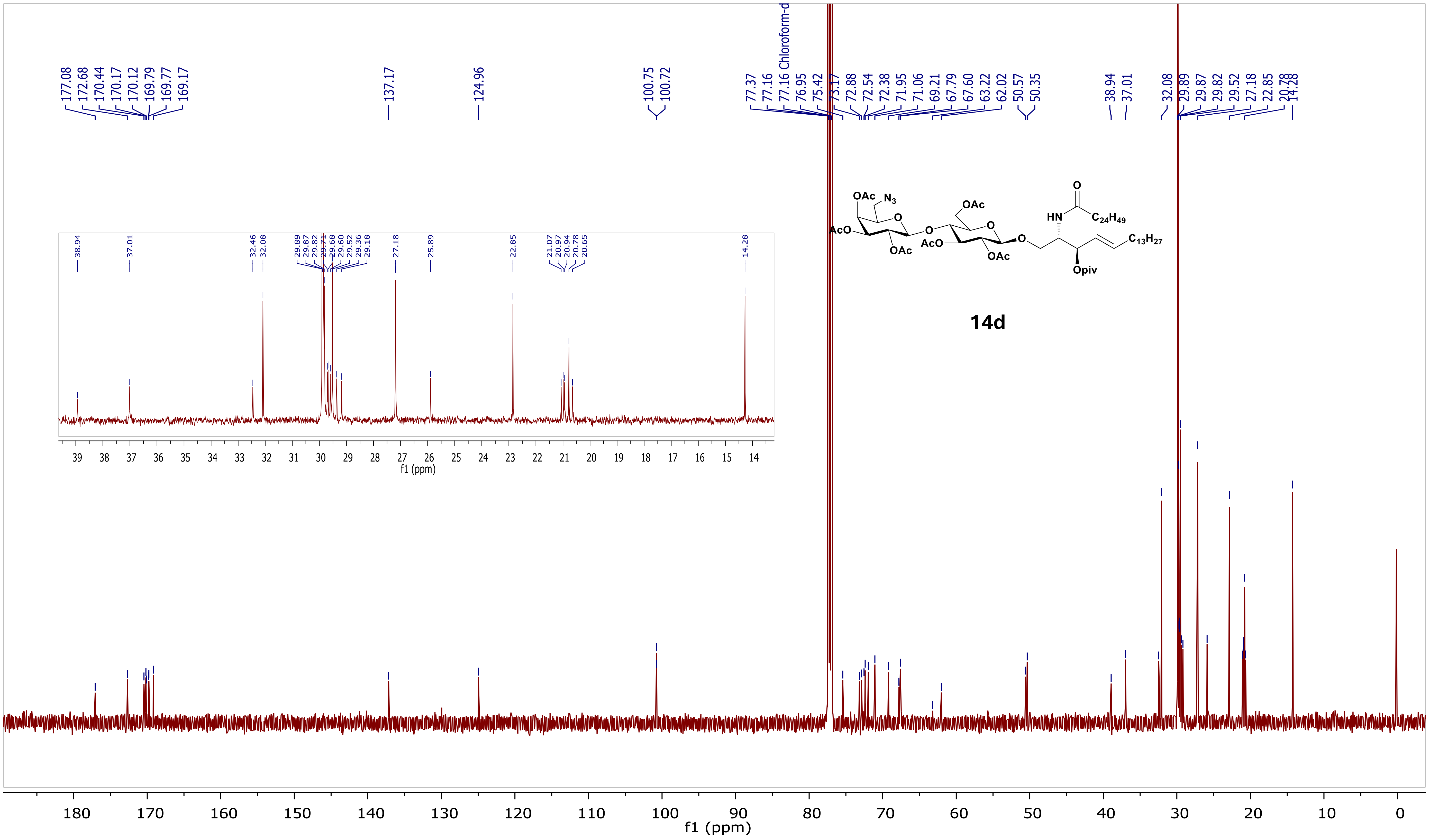

Figure S67. ^{1}H-^{1}H COSY NMR (600 MHz, $CDCl_3$) of compound **14d**

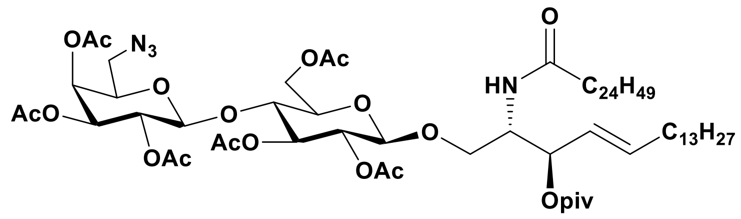

14d

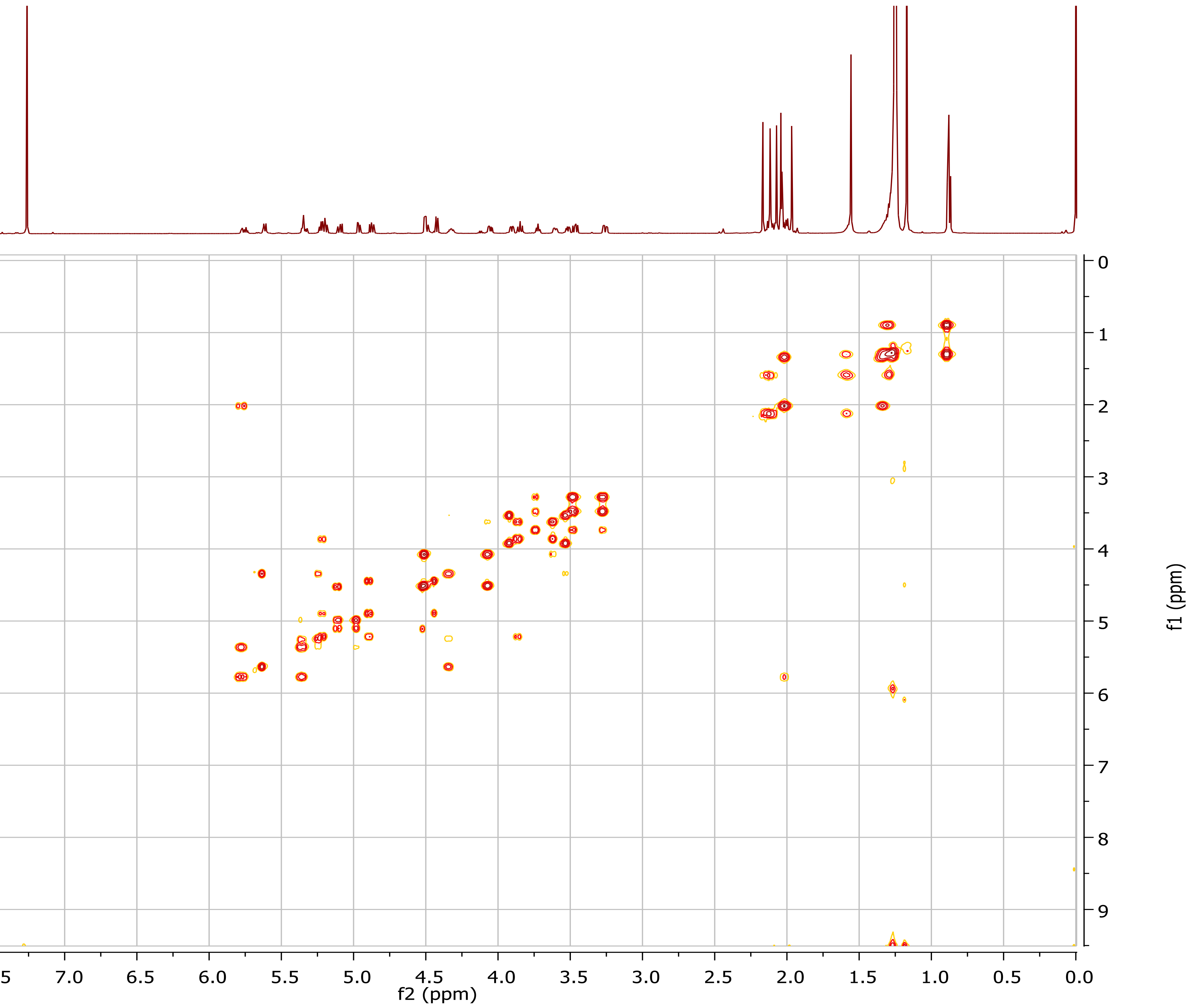

Figure S68. ^{1}H-^{13}C HSQC NMR (600/151 MHz, $CDCl_3$) of compound **14d**

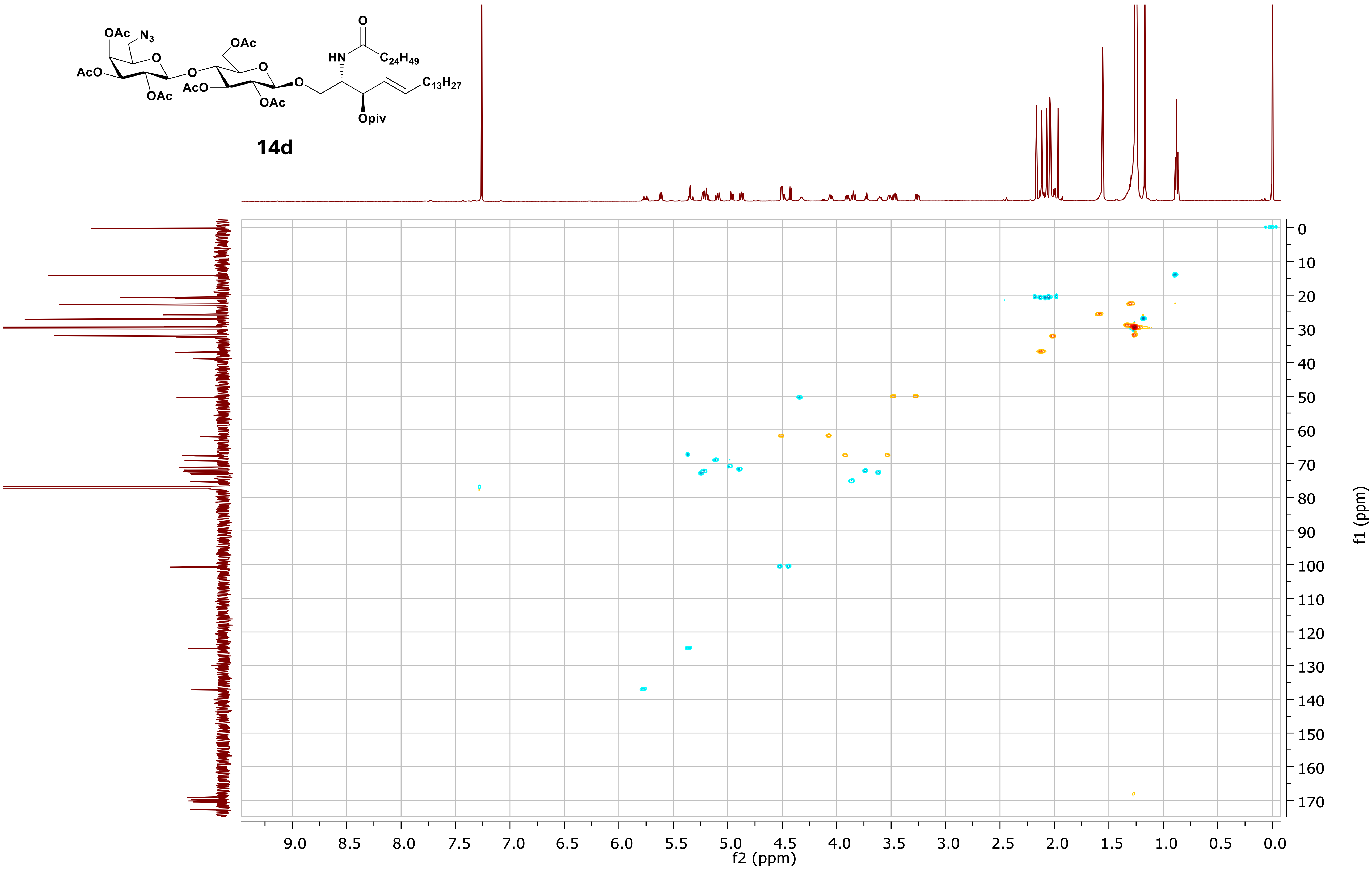

Figure S69. HR ESI-TOF-MS of compound **14d**

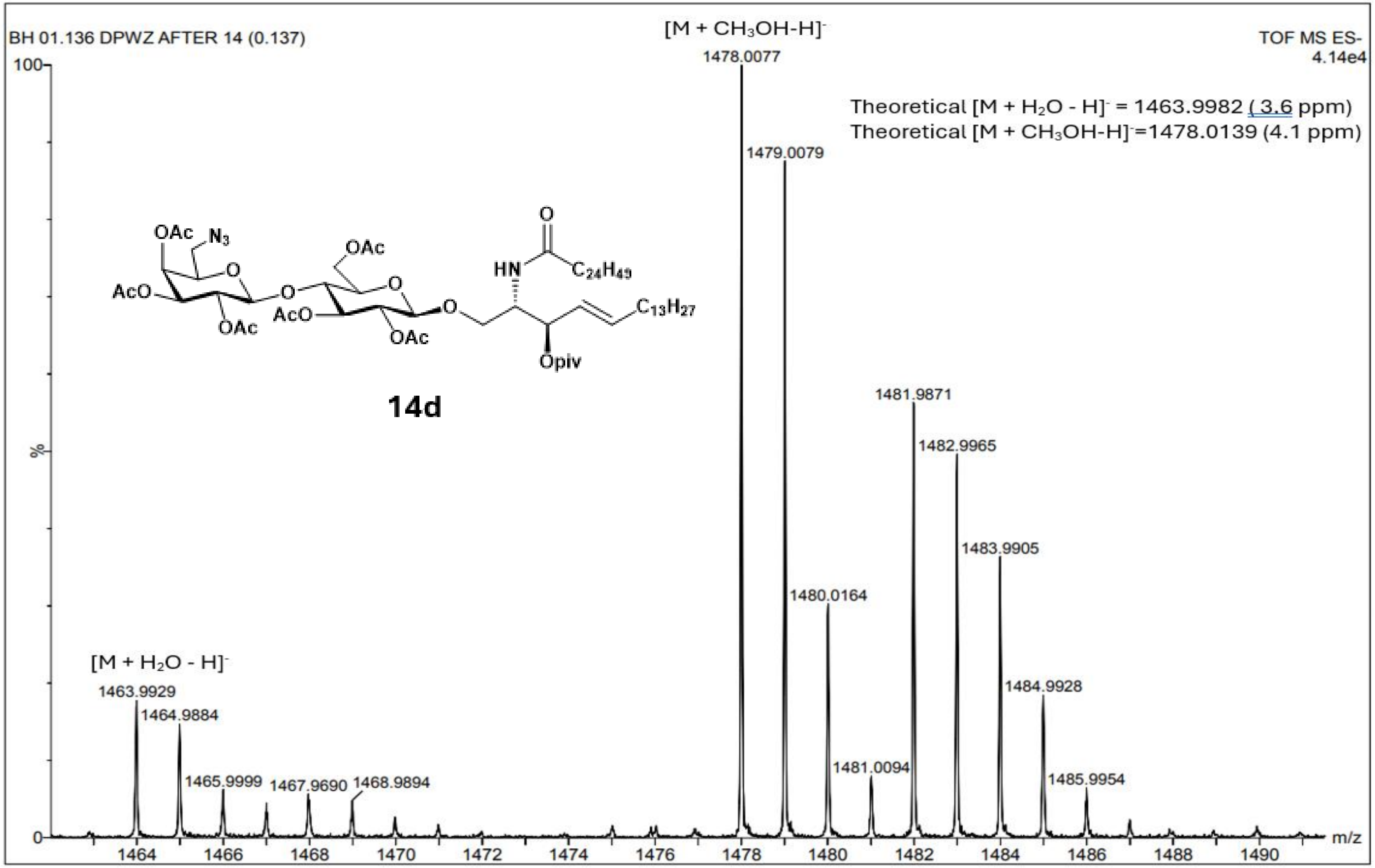

Figure S70. ^1H NMR of compound **1a** (600 MHz, $CDCl_3/CD_3OD$ 2:1)

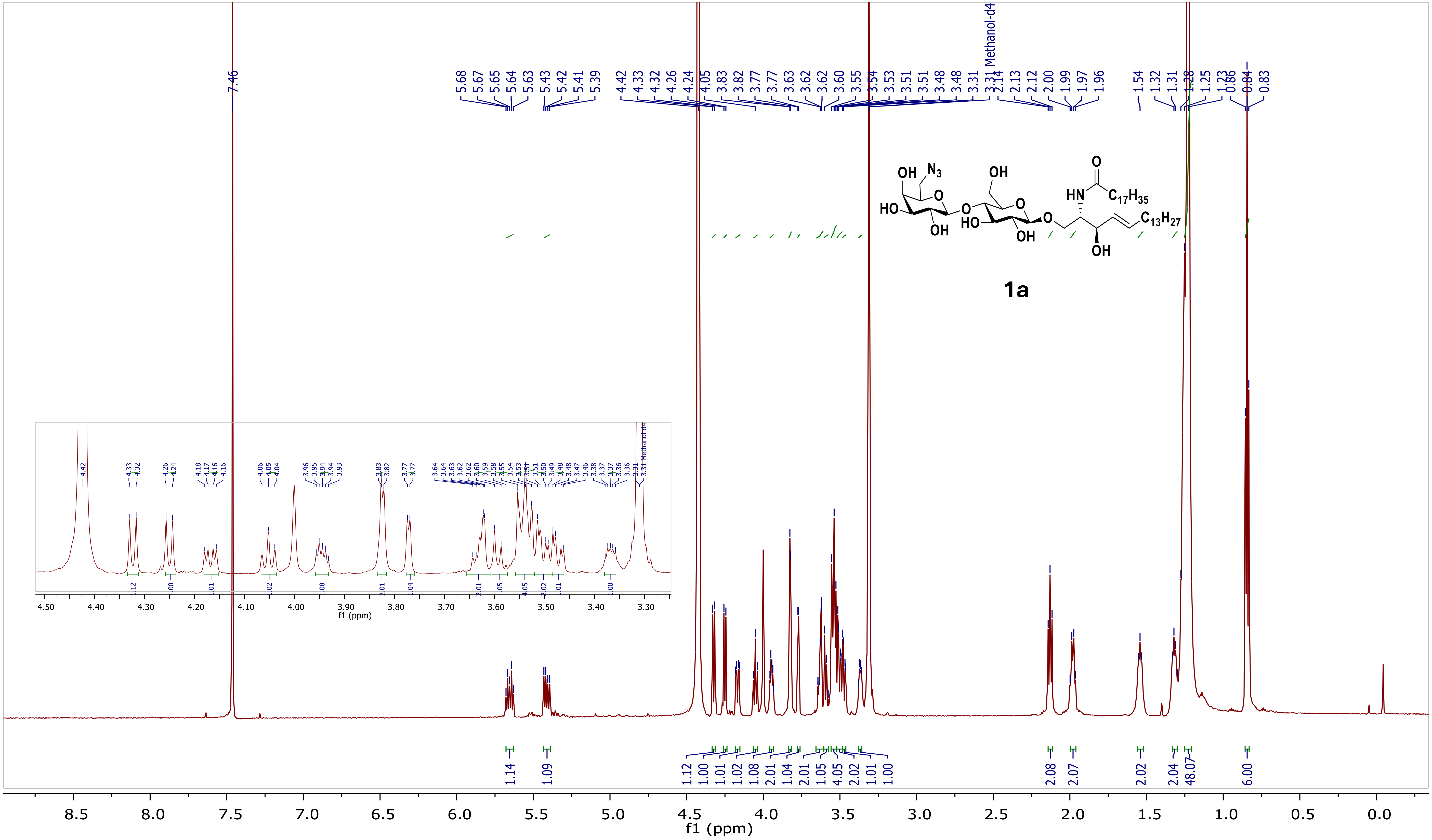

Figure S71. ^{13}C NMR of compound **1a** (151 MHz, $CDCl_3/CD_3OD$ 2:1)

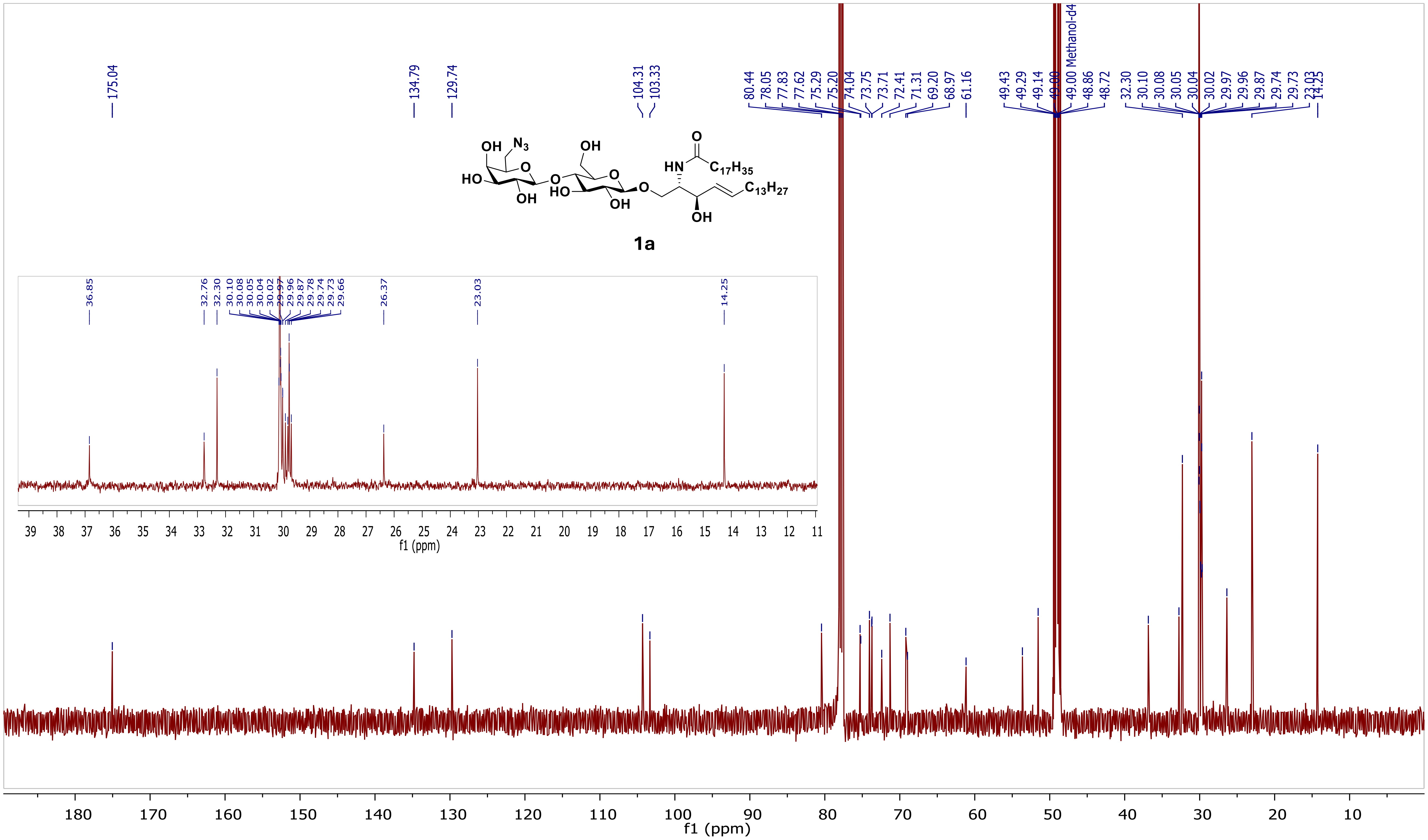

Figure S72. ^{1}H-^{1}H COSY NMR (600 MHz, $CDCl_3/CD_3OD$ 2:1) of compound **1a**

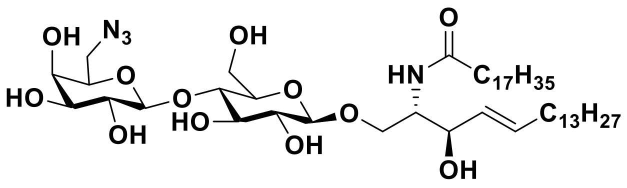

1a

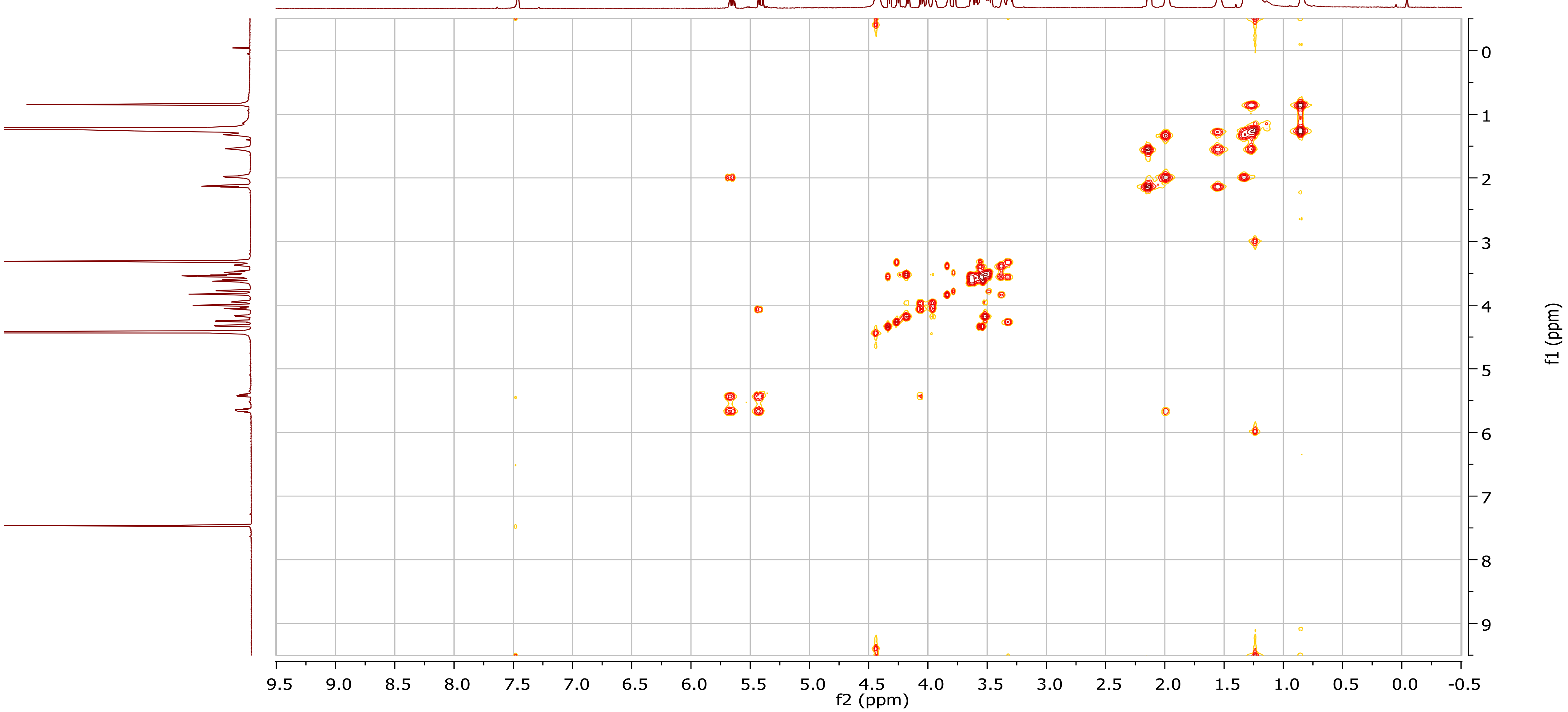

Figure S73. ^{1}H-^{13}C HSQC NMR (600/151 MHz, $CDCl_3/CD_3OD$ 2:1) of compound **1a**

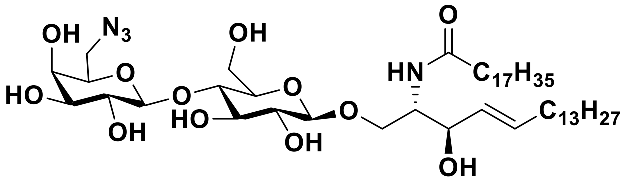

1a

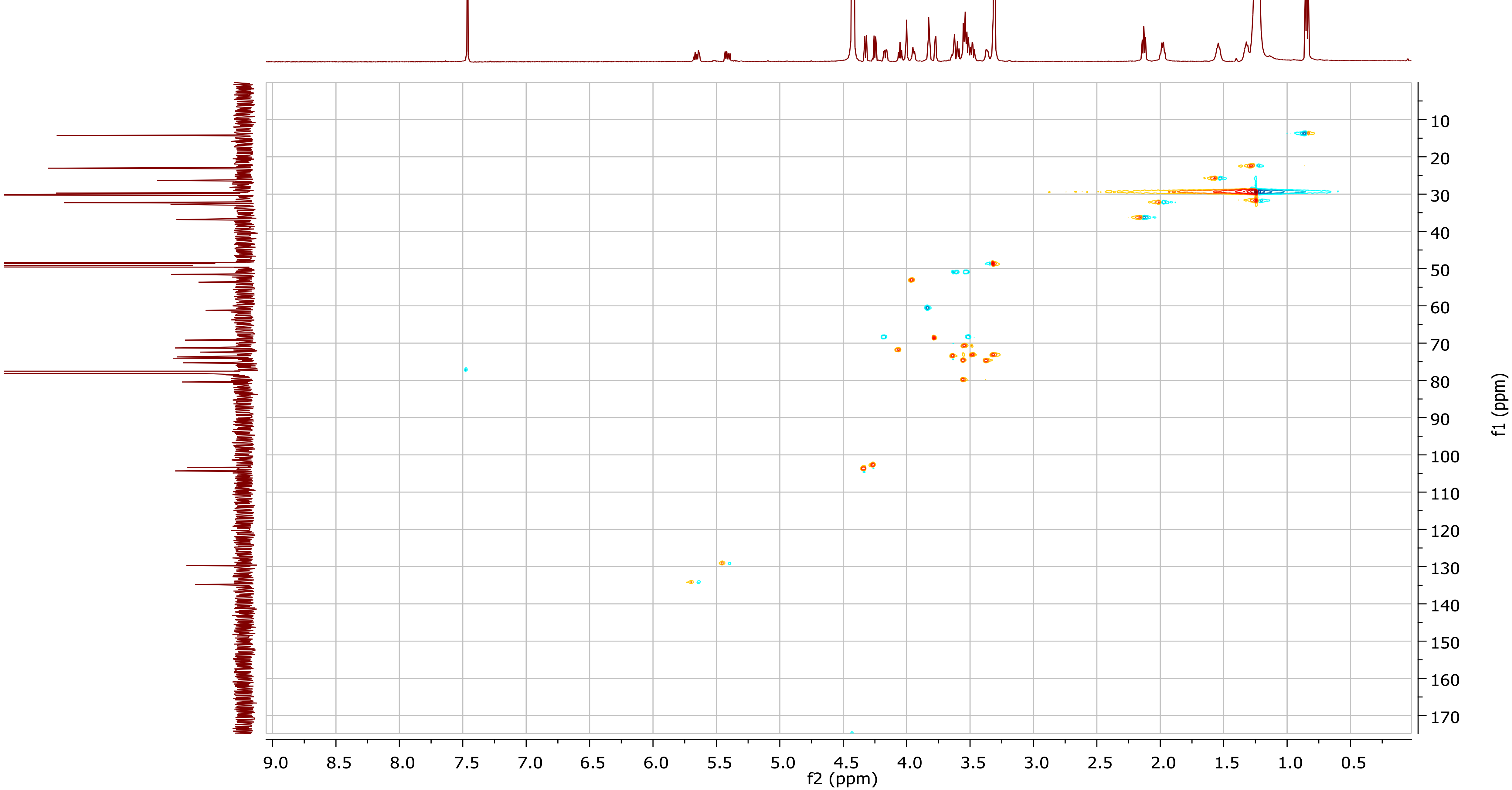

Figure S74. HR ESI-TOF-MS of compound **1a**

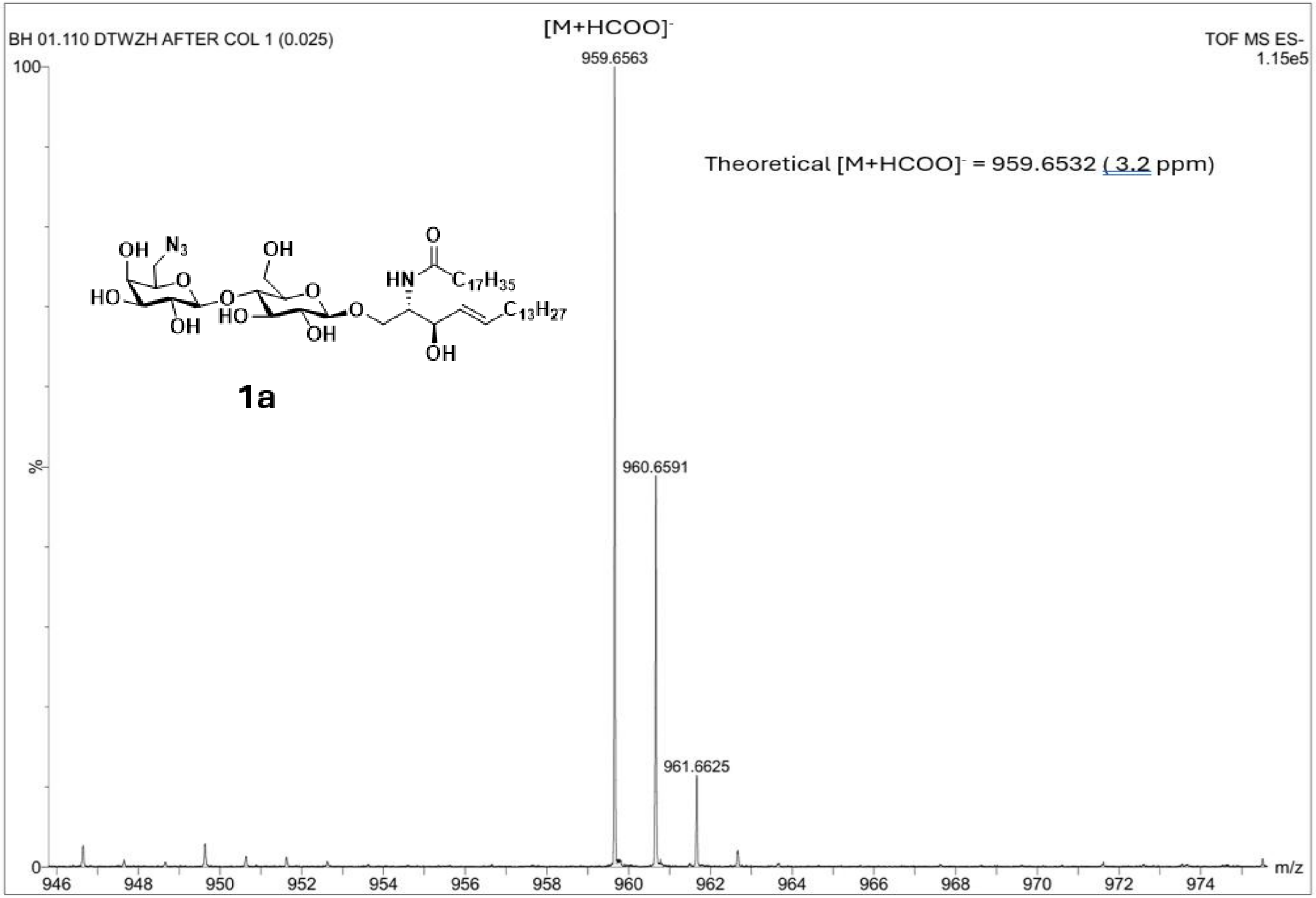

Figure S75. ^{1}H NMR of compound **1b** (600 MHz, $CDCl_3/CD_3OD$ 2:1)

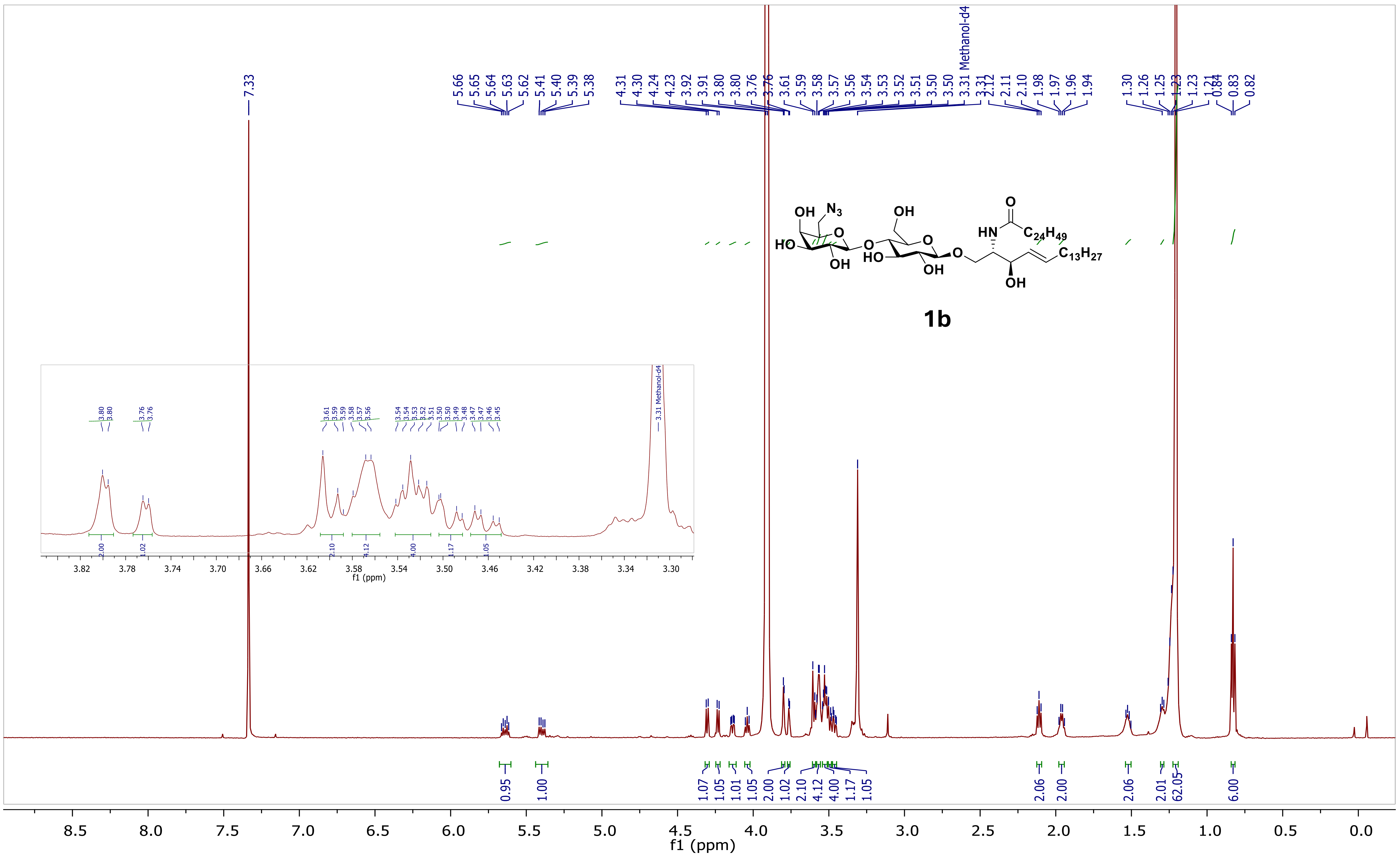

Figure S76. ^{13}C NMR of compound **1b** (151 MHz, $CDCl_3/CD_3OD$ 2:1)

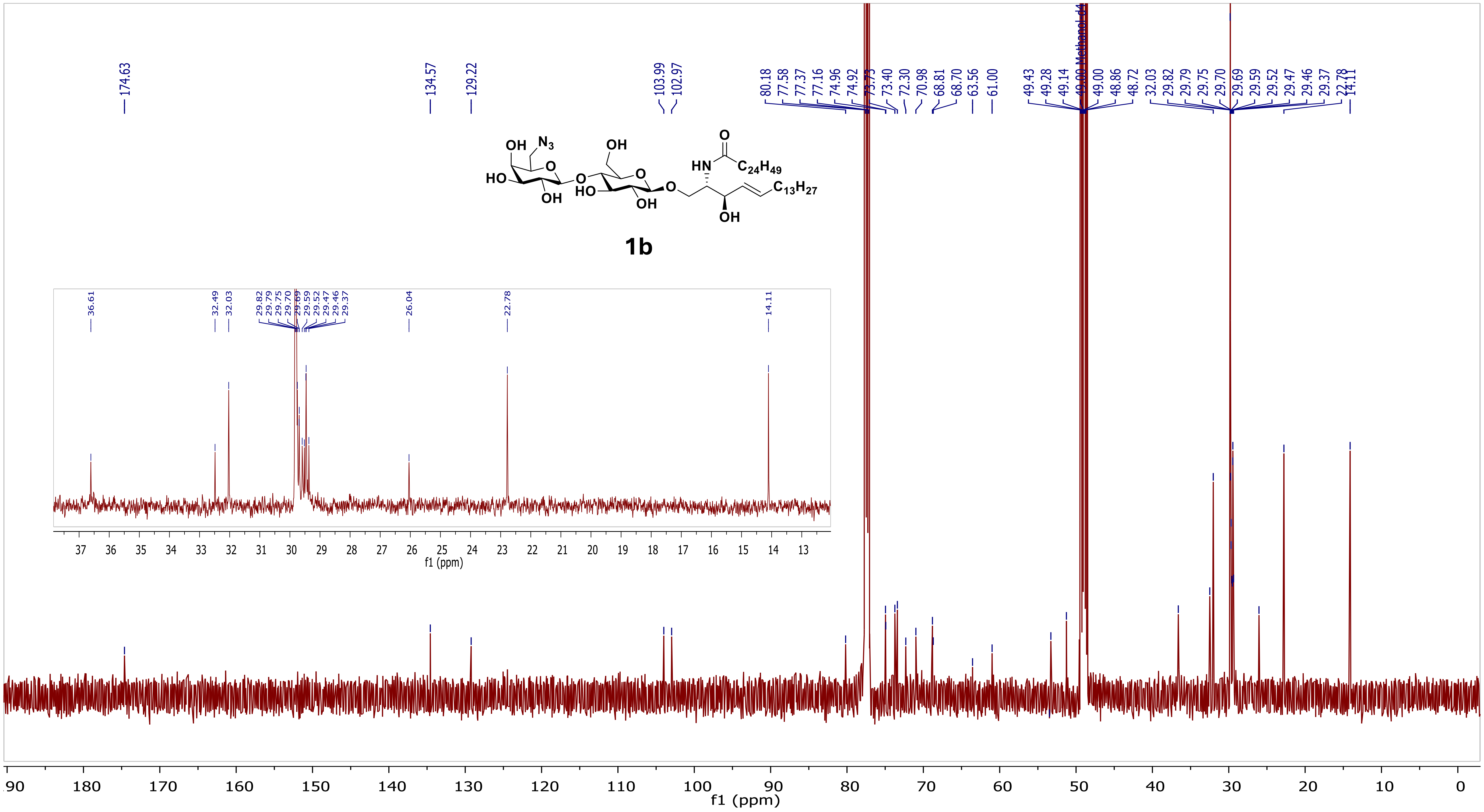

Figure S77. ^1H-^1H COSY NMR (600 MHz, $CDCl_3/CD_3OD$ 2:1) of compound **1b**

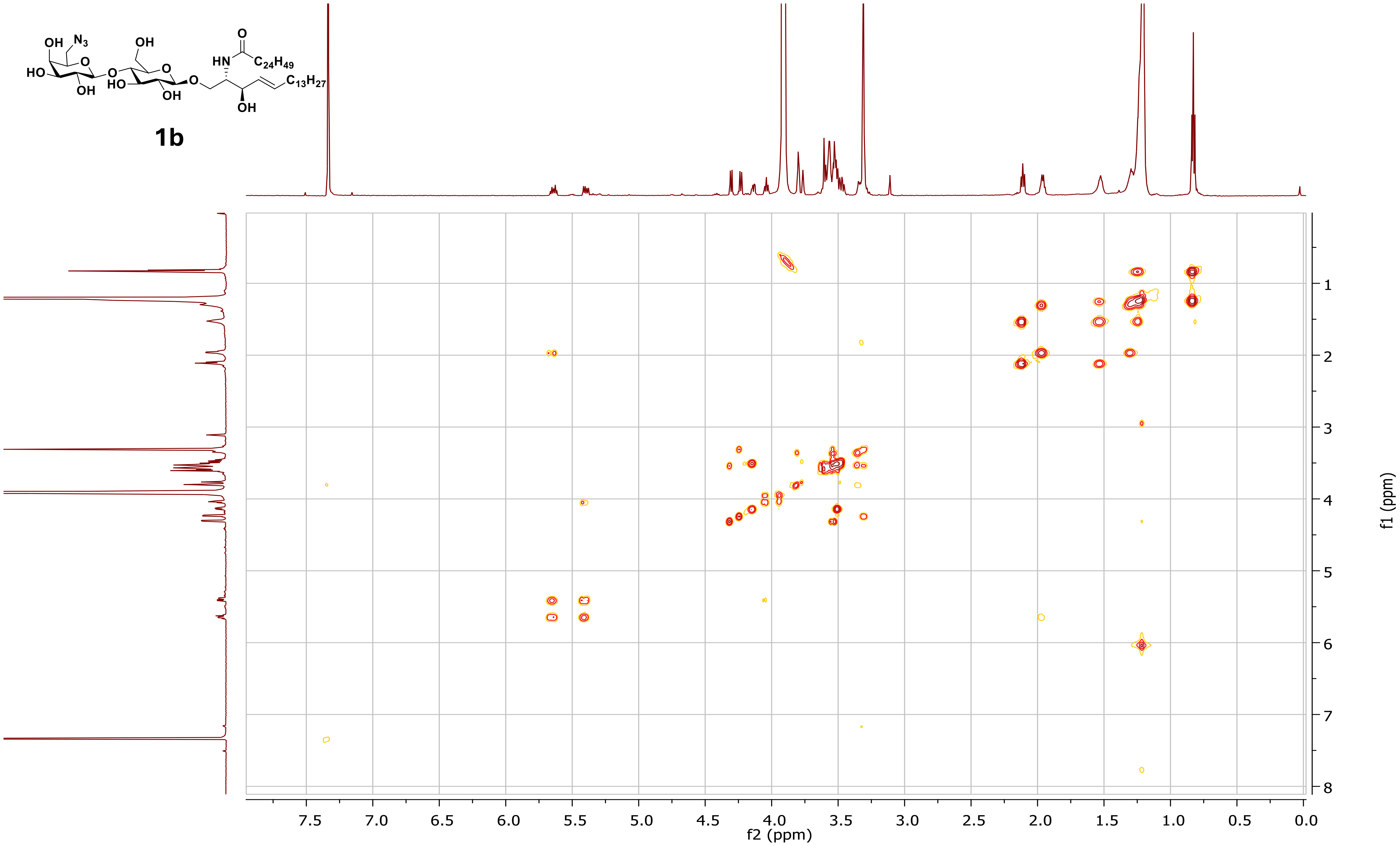

Figure S78. ^{1}H-^{13}C HSQC NMR (600/151 MHz, $CDCl_3/CD_3OD$ 2:1) of compound **1b**

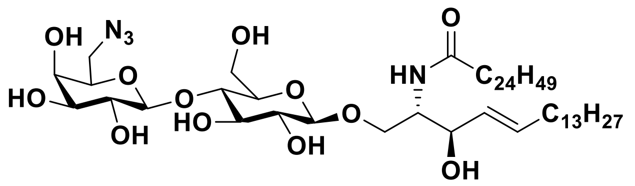

1b

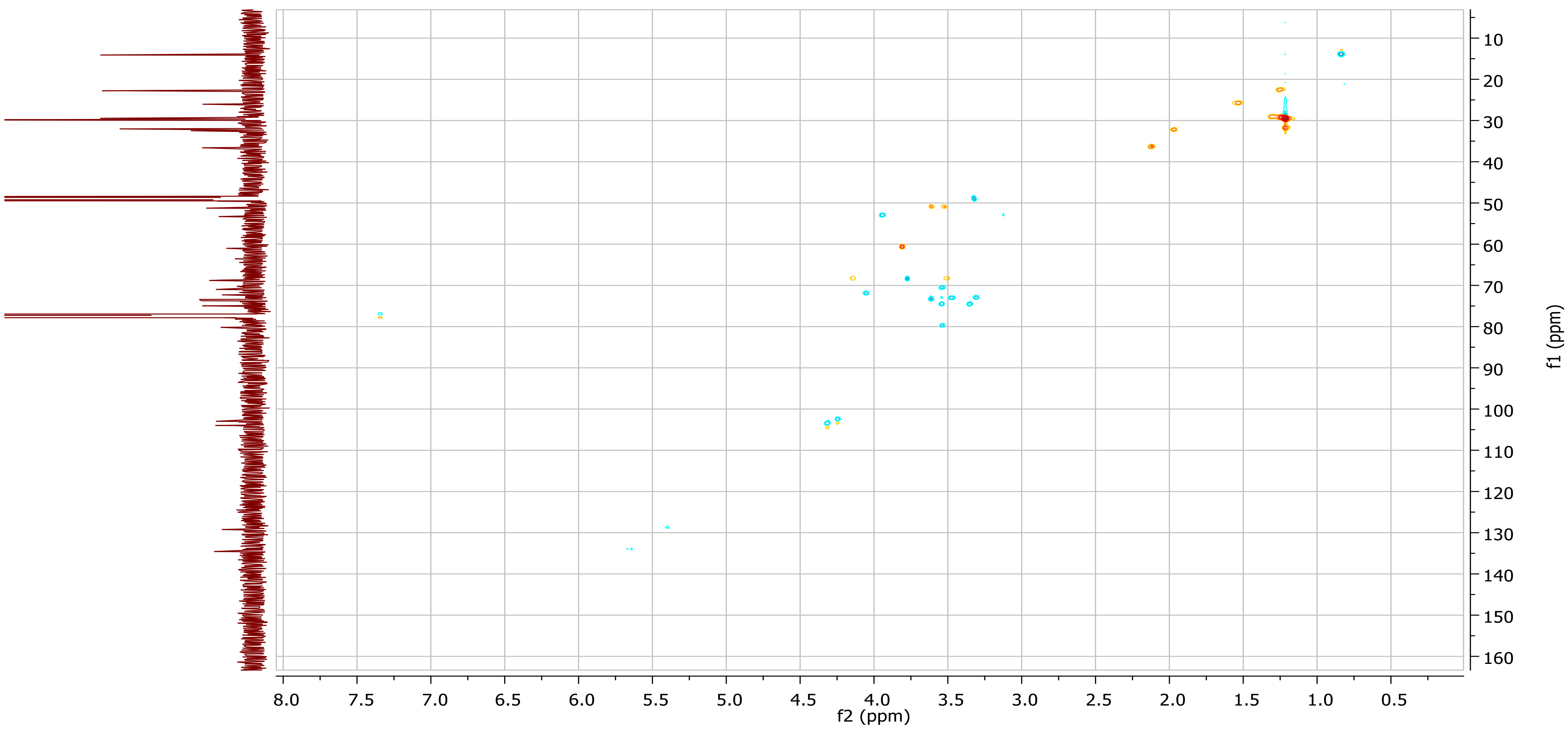

Figure S79. HR ESI-TOF-MS of compound **1b**

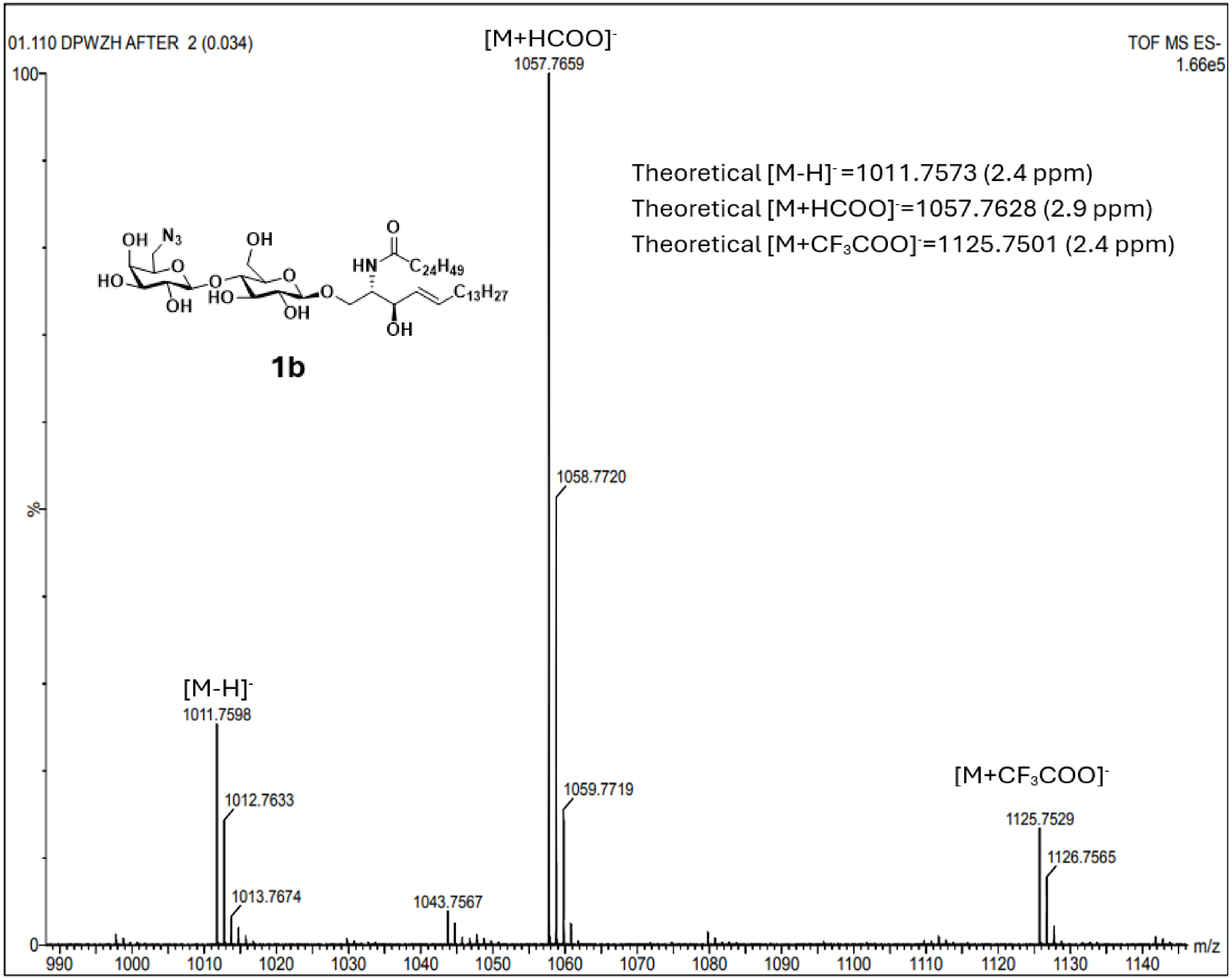

Figure S80. ^{1}H NMR of compound **1c** (600 MHz, $CDCl_3/CD_3OD$ 2:1)

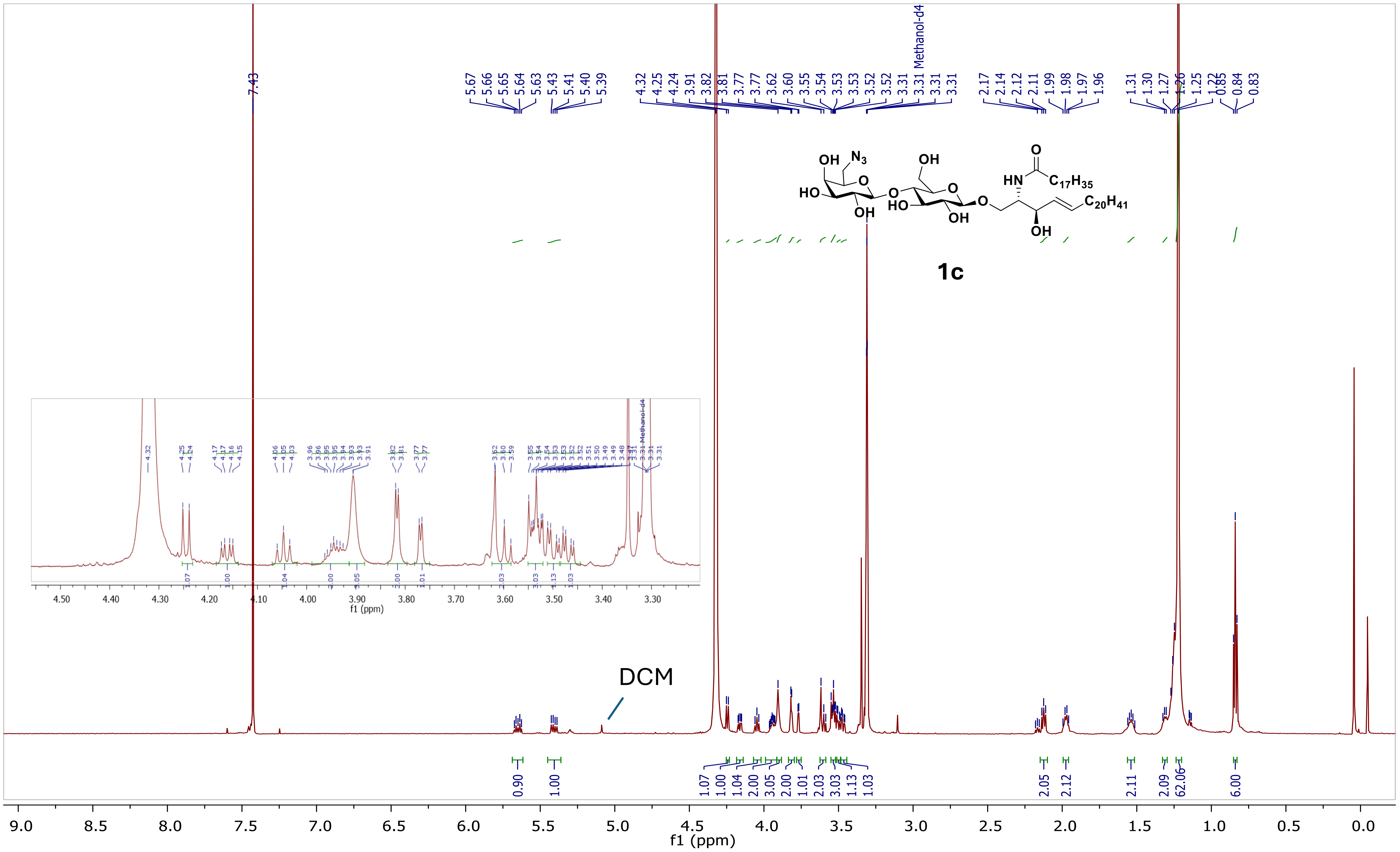

Figure S81. ^{13}C NMR of compound **1c** (151 MHz, $CDCl_3/CD_3OD$ 2:1)

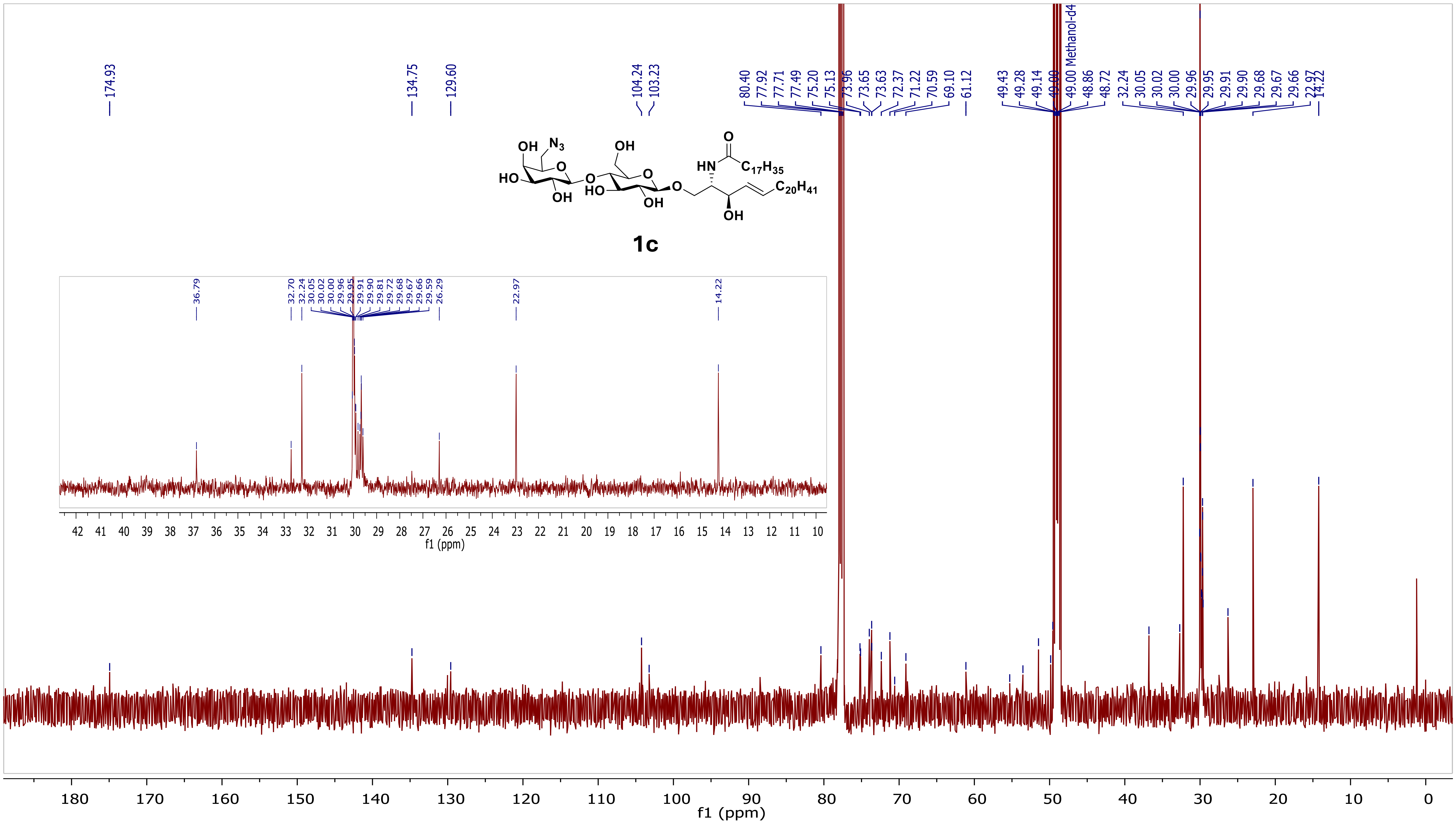

Figure S82. ^{1}H-^{1}H COSY NMR (600 MHz, $CDCl_3/CD_3OD$ 2:1) of compound **1c**

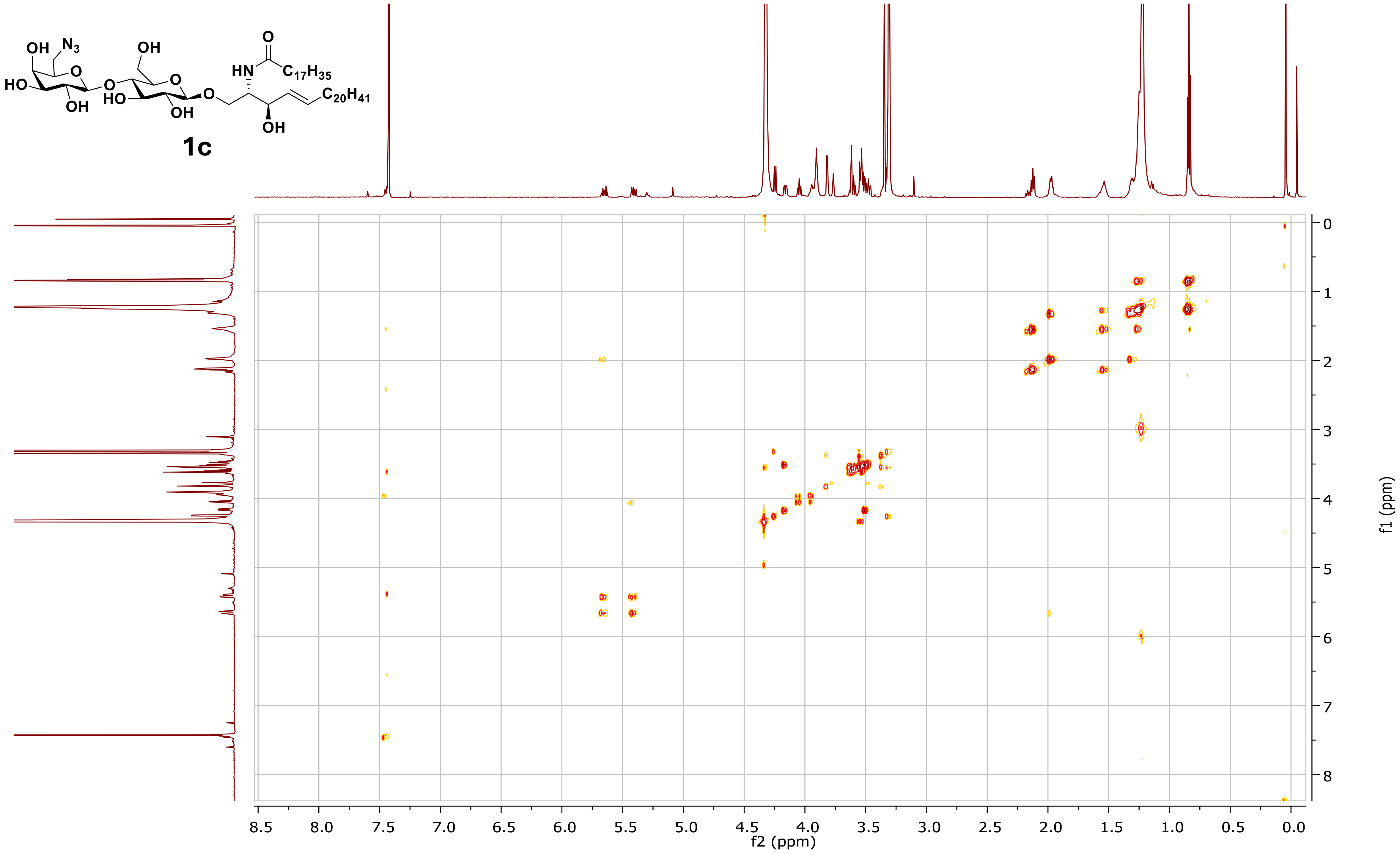

1c

Figure S83. ^{1}H-^{13}C HSQC NMR (600/151 MHz, $CDCl_3/CD_3OD$ 2:1) of compound **1c**

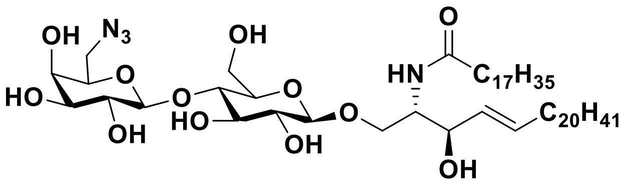

1c

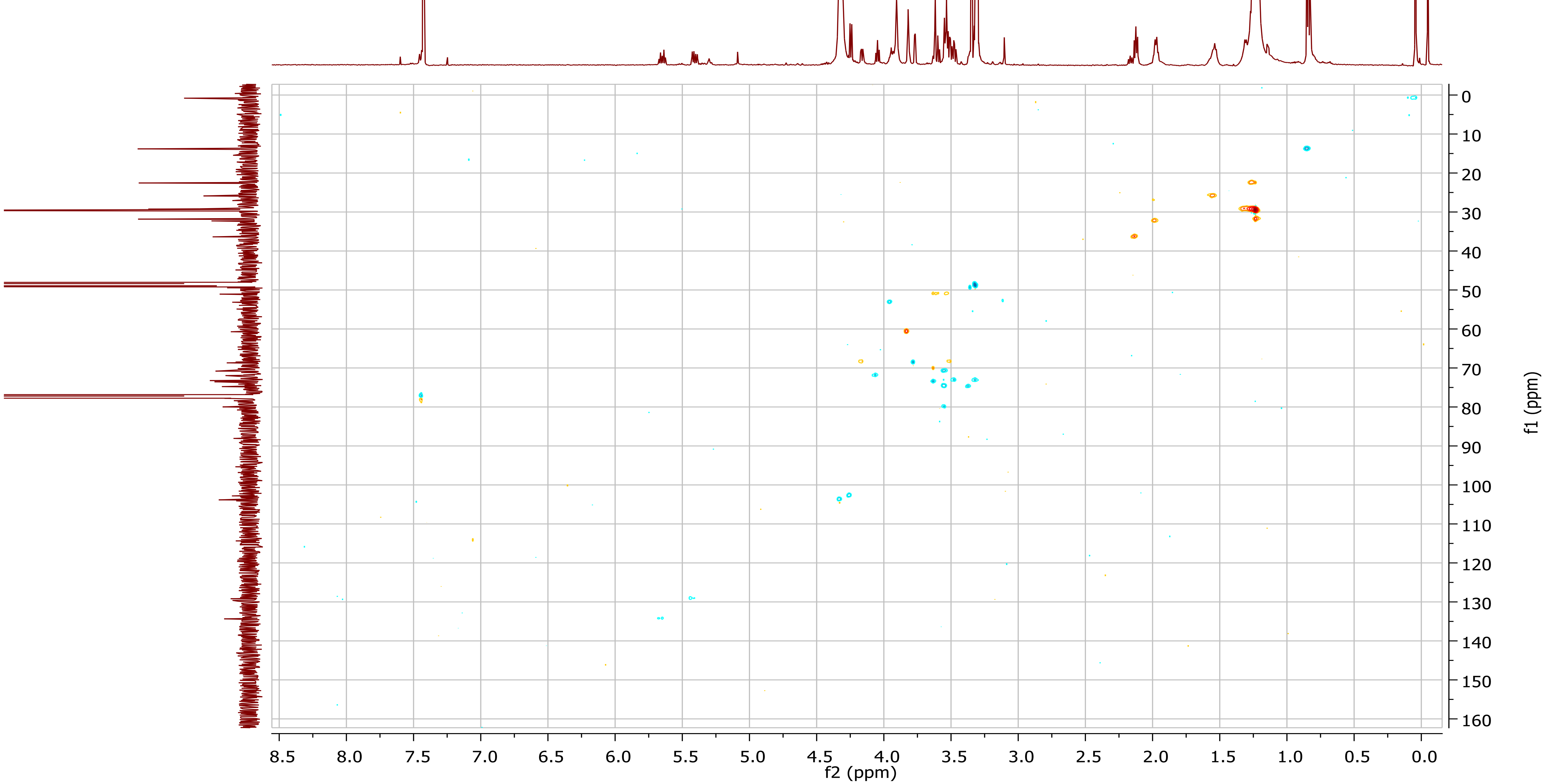

Figure S84. HR ESI-TOF-MS of compound **1c**

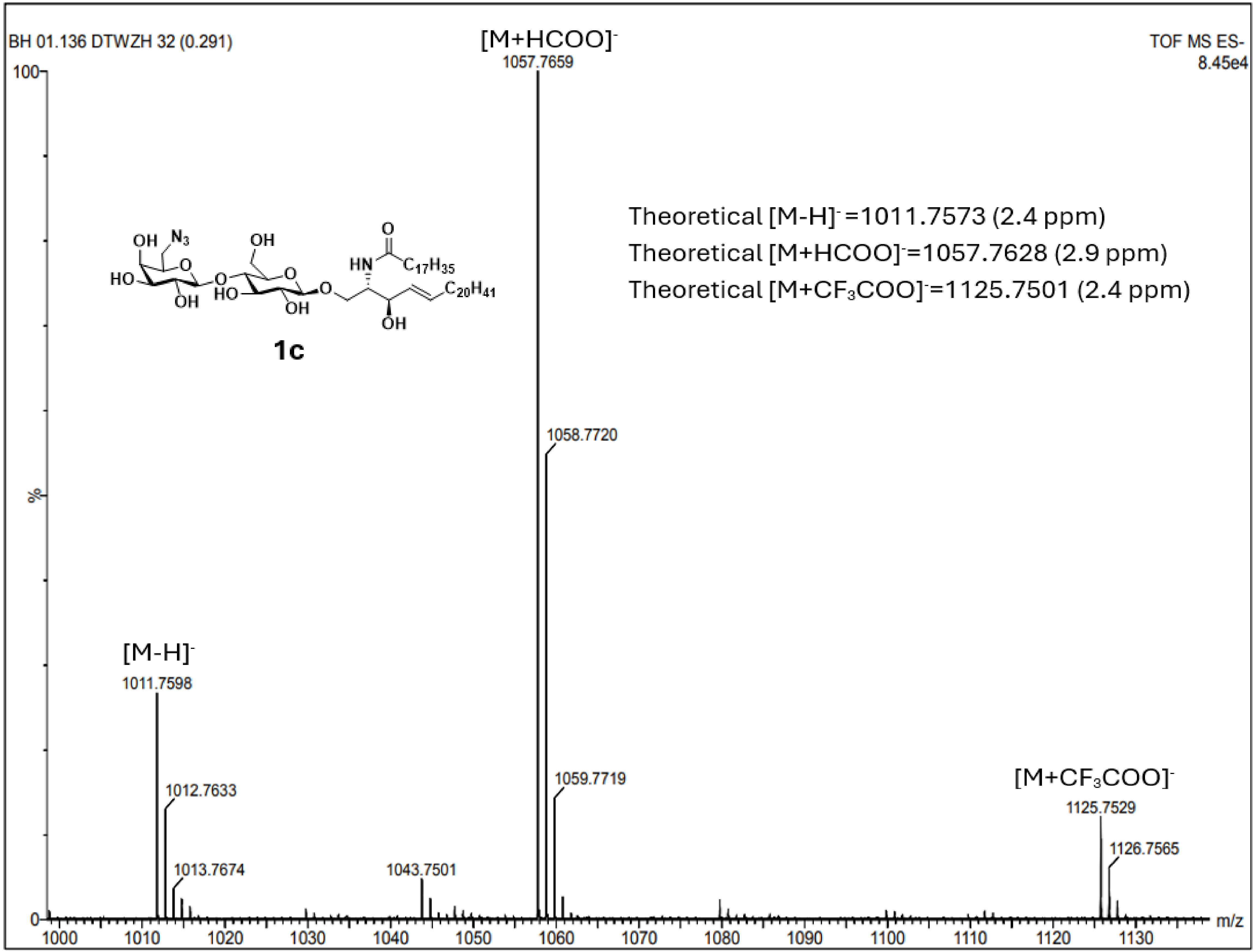

Figure S85. ^1H NMR of compound **1d** (600 MHz, $CDCl_3/CD_3OD$ 2:1)

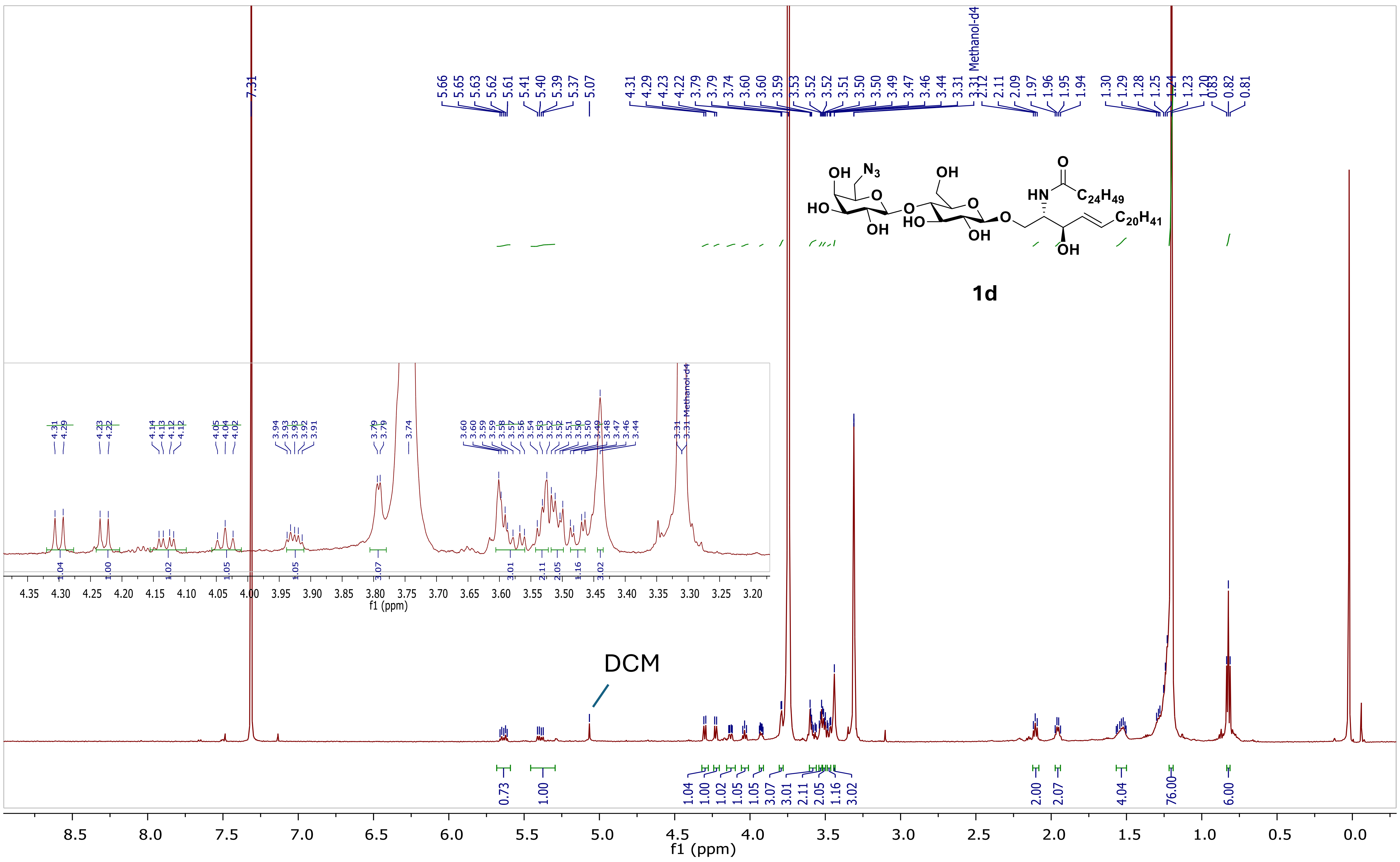

Figure S86. ^{13}C NMR of compound **1d** (151 MHz, $CDCl_3/CD_3OD$ 2:1)

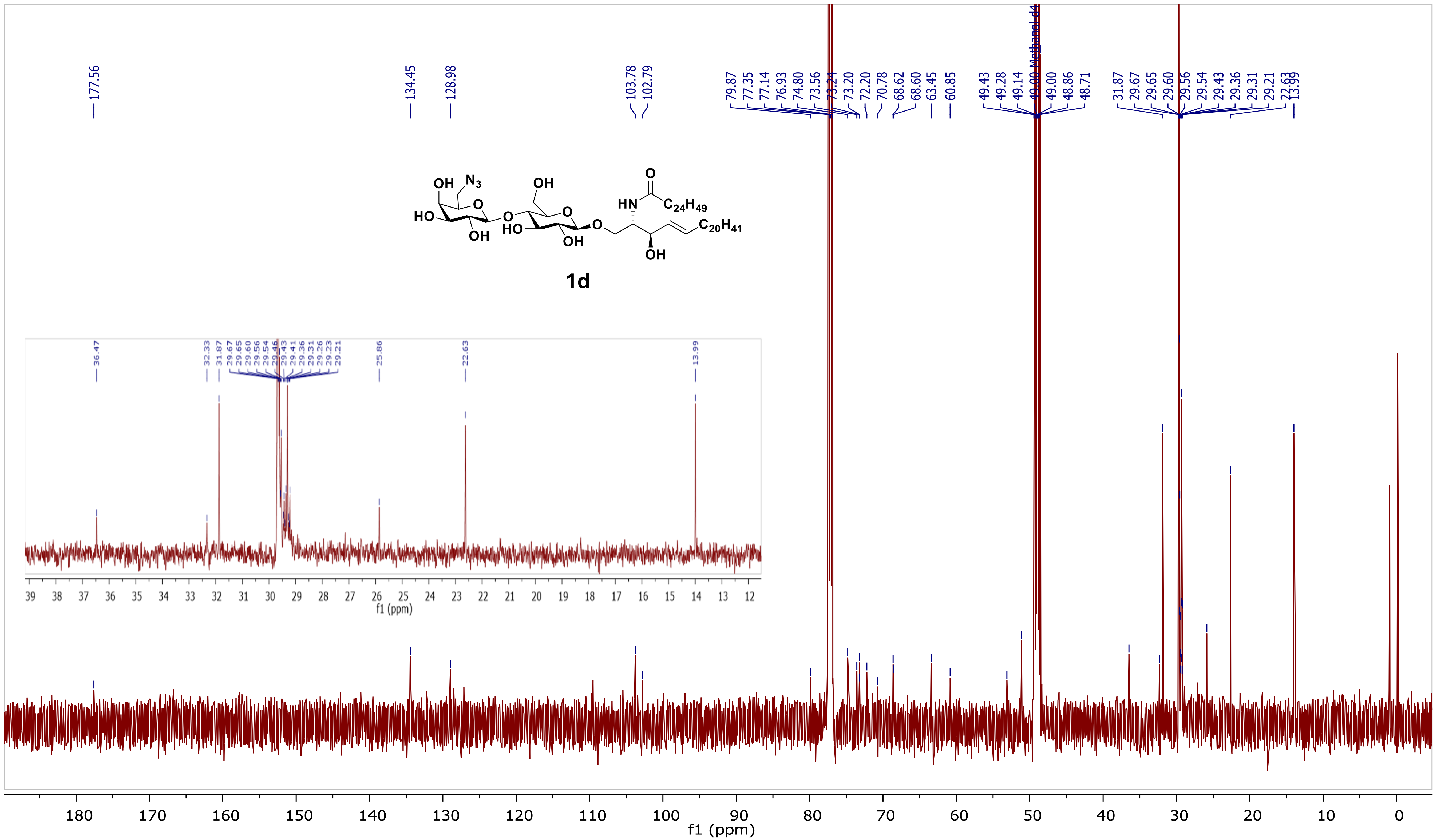

Figure S87. ^{1}H-^{1}H COSY NMR (600 MHz, $CDCl_3/CD_3OD$ 2:1) of compound **1d**

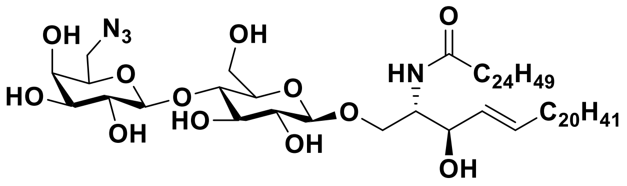

1d

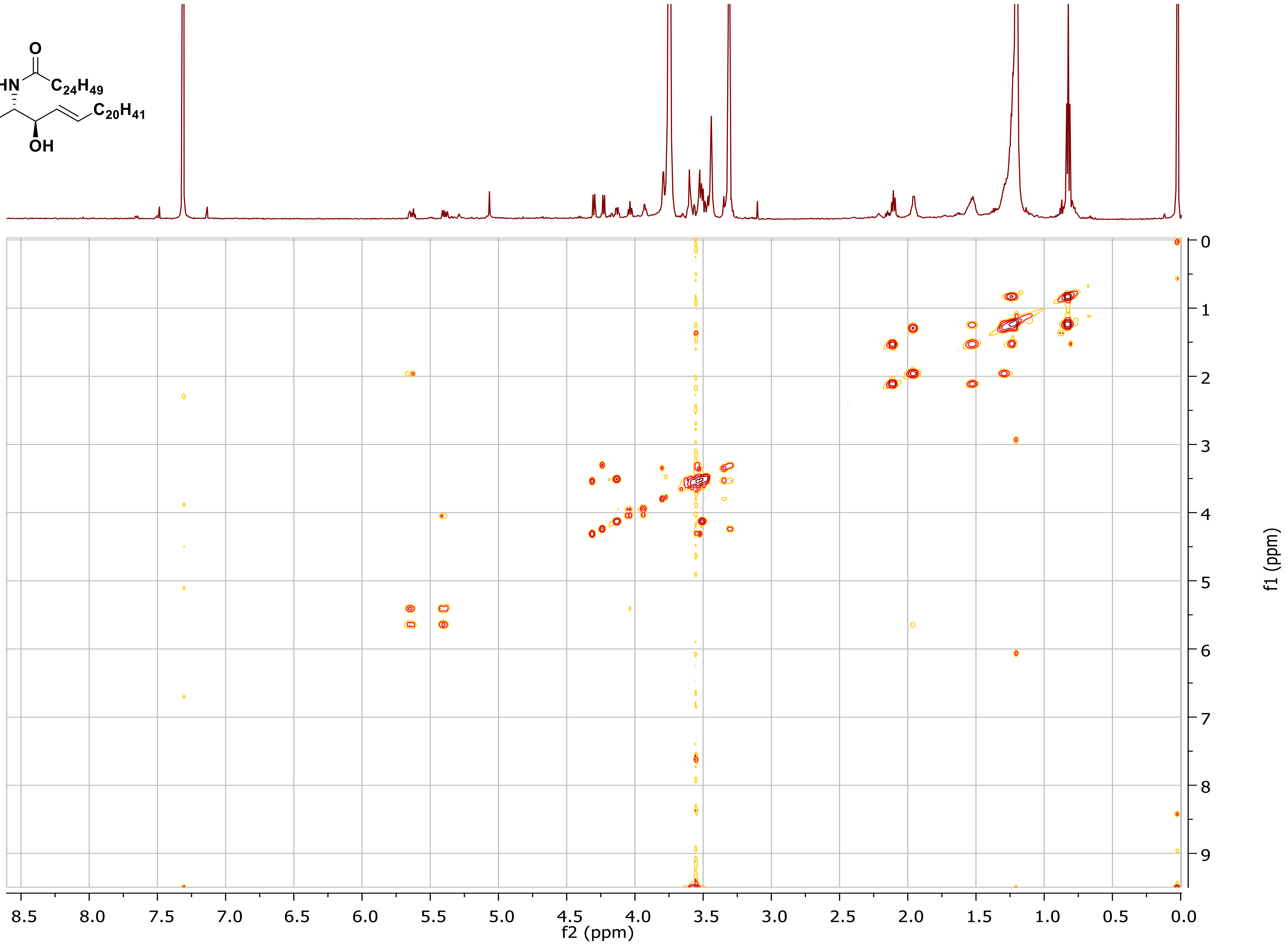

Figure S88. ^{1}H-^{13}C HSQC NMR (600/151 MHz, $CDCl_3/CD_3OD$ 2:1) of compound **1d**

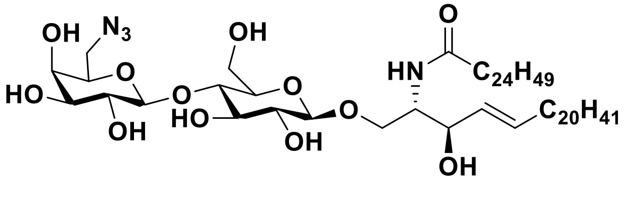

1d

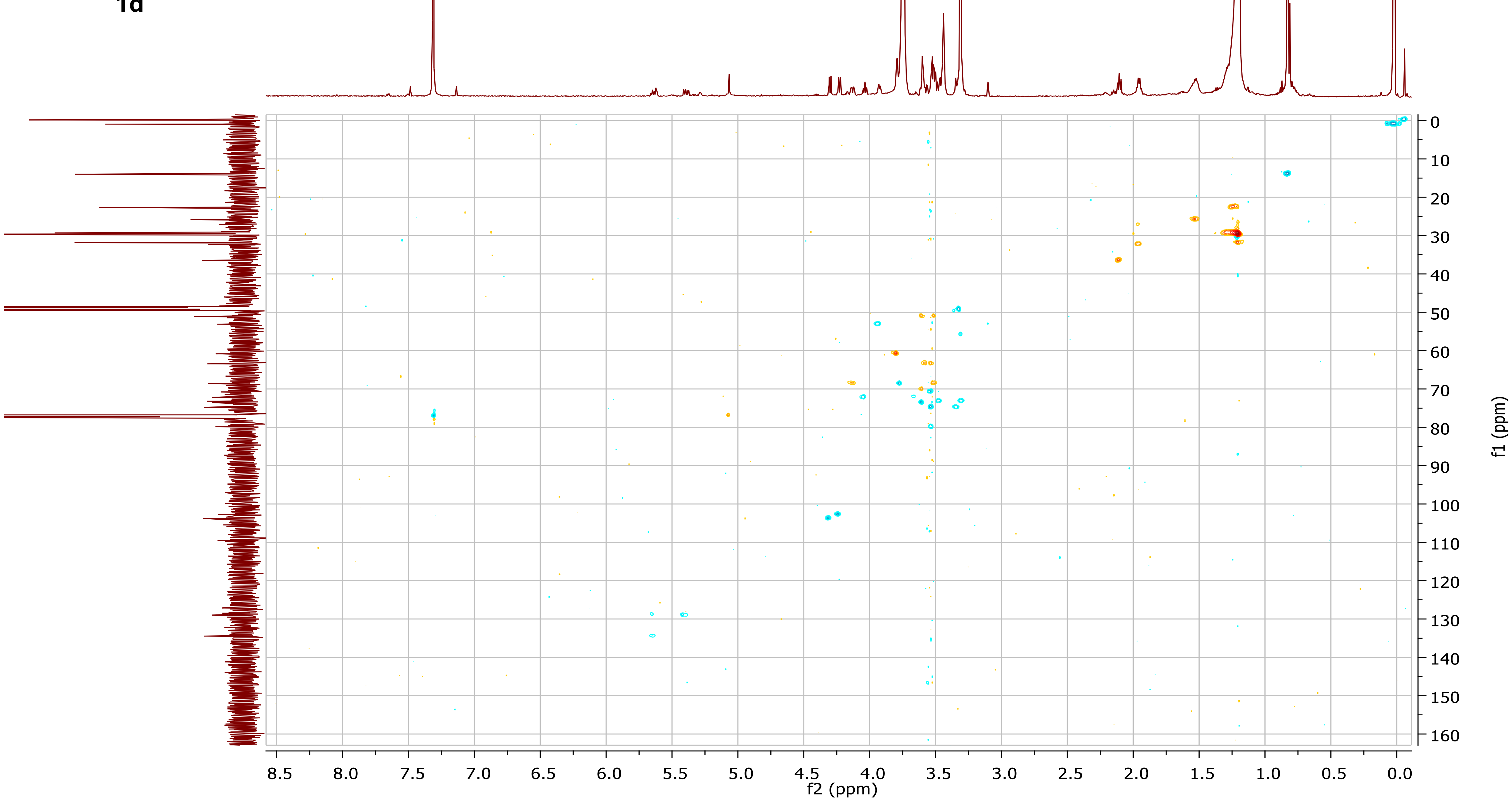

Figure S89. HR ESI-TOF-MS of compound **1d**

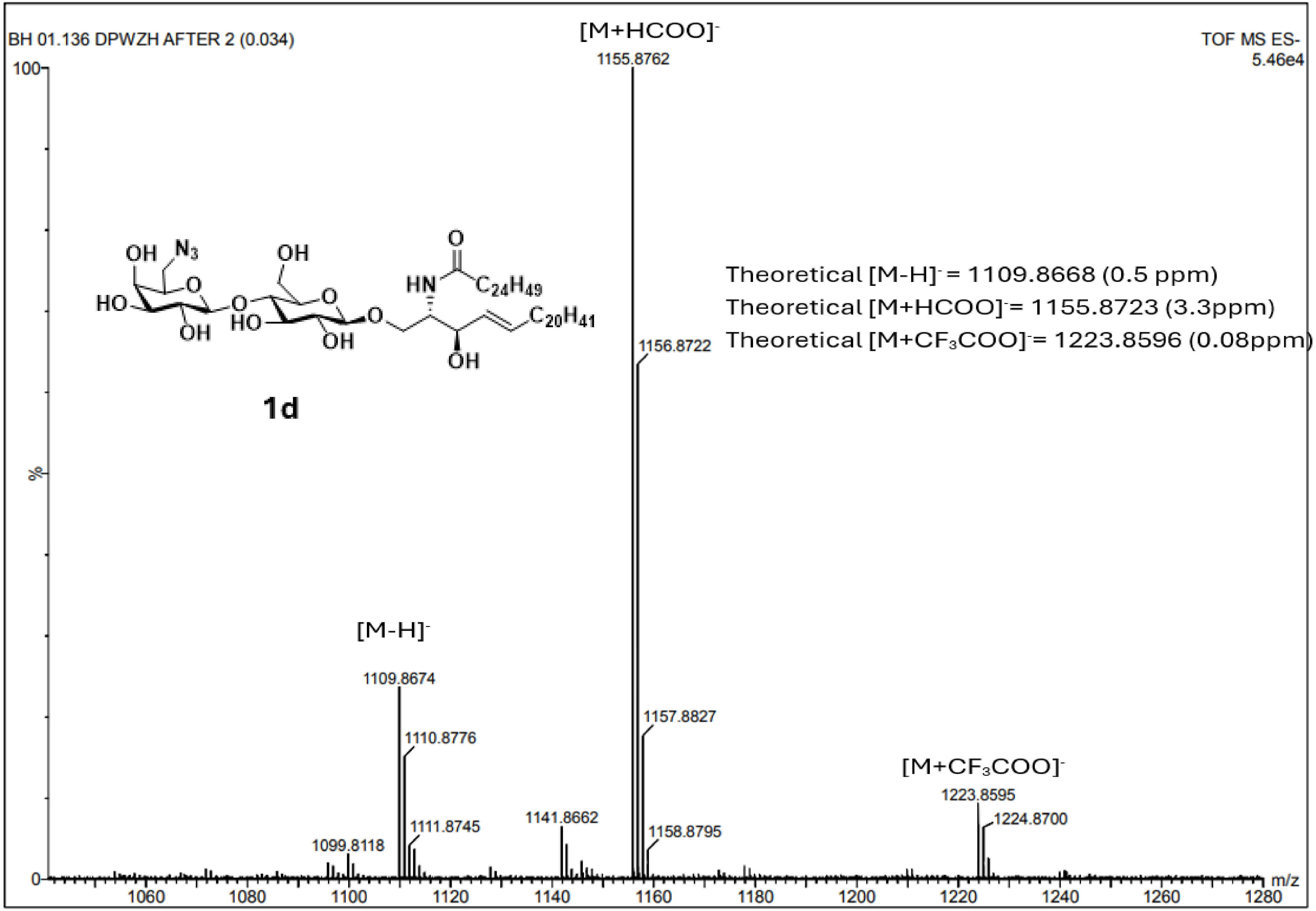

Supplement: Supplementary file 1 [file molecules-30-02667-s001.zip › molecules-3710138-supplementary.pdf]
